# Unraveling the associations and causalities between glucose metabolism and multiple sleep traits

**DOI:** 10.3389/fendo.2023.1227372

**Published:** 2023-11-07

**Authors:** Minhan Yi, Quanming Fei, Ziliang Chen, Wangcheng Zhao, Kun Liu, Shijie Jian, Bin Liu, Meng He, Xiaoli Su, Yuan Zhang

**Affiliations:** ^1^ Department of Respiratory Medicine, Xiangya Hospital, Central South University, Changsha, China; ^2^ School of Life Sciences, Central South University, Changsha, China; ^3^ National Clinical Research Center for Geriatric Disorders, Xiangya Hospital, Central South University, Changsha, China; ^4^ Xiangya Medical School, Central South University, Changsha, China

**Keywords:** EDS, insomnia, sleep duration, glycemic trait, Mendelian randomization

## Abstract

**Purpose:**

The aim of our study is to estimate the associations and causalities of glucose metabolism traits of fasting blood glucose (FBG), fasting insulin (FINS), glycosylated hemoglobin (HbA1c), and 2-h glucose post-challenge (2hGlu) with sleep traits consisting of excessive daytime sleepiness (EDS), insomnia, and sleep duration.

**Methods:**

We employed standard quantitative analysis procedures to assess the associations between sleep traits and glucose metabolism. Moreover, we acquired published genome-wide association studies (GWAS) summary statistics for these traits and conducted Mendelian randomization (MR) analyses to estimate their causal directions and effects. Inverse variance weighting (IVW) was employed as the primary approach, followed by sensitivity analyses.

**Results:**

A total of 116 studies with over 840,000 participants were included in the quantitative analysis. Our results revealed that participants with abnormal glucose metabolism had higher risks for EDS (OR [95% CI] = 1.37 [1.10,1.69]), insomnia (OR [95% CI] = 1.65 [1.24,2.20]), and both short and long sleep duration (OR [95% CI] = 1.35 [1.12,1.63]; OR [95% CI] = 1.38 [1.13,1.67] respectively). In addition, individuals with these sleep traits exhibited alterations in several glycemic traits compared with non-affected controls. In MR analysis, the primary analysis demonstrated causal effects of 2hGlu on risks of EDS (OR [95% CI] = 1.022 [1.002,1.042]) and insomnia (OR [95% CI] = 1.020[1.001,1.039]). Furthermore, FINS was associated with short sleep duration (OR [95% CI] = 1.043 [1.018,1.068]), which reversely presented a causal influence on HbA1c (β [95% CI] = 0.131 [0.022,0.239]). These results were confirmed by sensitivity analysis.

**Conclusion:**

Our results suggested mutual risk and causal associations between the sleep traits and glycemic traits, shedding new light on clinical strategies for preventing sleep disorders and regulating glucose metabolism. Future studies targeting these associations may hold a promising prospect for public health.

## Introduction

Diabetes mellitus (DM), affecting over 350 million people worldwide and bringing over 1,200 billion USD in economic burden, is one of the leading causes of death (1, 2). DM can also result in various outcomes that have a high degree of mortality and morbidity, such as cardiovascular diseases, neuropathy, nephropathy, retinopathy, diabetic foot ulcers, and many other diseases (3). Glucose metabolism can be well reflected by traits of fasting blood glucose (FBG), fasting insulin (FINS), glycosylated hemoglobin (HbA1c), and 2-h glucose post-challenge (2hGlu). FBG and FINS levels refer to glucose and insulin levels in the blood after an overnight fast, which were respectively used to assess baseline blood glucose level, and insulin production and insulin resistance (4, 5). 2hGlu levels, usually treated as part of an oral glucose tolerance test (OGTT), refer to the measurement of glucose levels in the blood 2 h after consuming a standardized glucose load (6). HbA1c levels measure the percentage of hemoglobin molecules that have glucose attached to them, reflecting the average blood sugar level over the previous 2 to 3 months (7). The relationship between these glycemic traits can vary depending on the scenario of abnormal glucose metabolism, such as diabetes, impaired fasting glucose (IFG), impaired glucose tolerance (IGT), hyperinsulinemia, and insulin resistance (IR). Since various glycemic traits signify distinct irregularities in glucose metabolism, approaching research from the standpoint of glycemic traits will yield more comprehensive insights into unraveling the interrelated pathogenic pathways involving glucose metabolism. Furthermore, identifying modifiable risk factors is beneficial in reducing the underdiagnosis and the occurrence of DM and related complications, ultimately improving quality of life and reducing healthcare costs.

Sleep constitutes approximately one-third of an individual’s life. Excessive daytime sleepiness (EDS), insomnia, and extreme sleep duration are highly prevalent sleep traits, demonstrating strong representativeness across different aspects of sleep (8, 9). Previous studies focused on associations between DM and sleep traits. Lin et al. conducted a cohort study with a large sample size of 28,390 insomnia patients and 57,413 controls, and they found that there were significant higher cumulative incidence of type 2 diabetes mellitus (T2DM) in the insomnia group than unaffected controls at 1, 5, and 10 years of follow-up (10). Shan Z and his colleague performed a meta-analysis using only prospective studies, and their results showed a U-shaped relationship between sleep duration and risk of T2DM, with both short and long sleep duration associated with a significantly increased risk of diabetes (11). Yusuf et al. performed a population-based cross-sectional study using data from the 2015–2018 National Health and Nutrition Examination Survey (NHANES). Their findings indicated a significant association between DM and a higher prevalence of EDS among American adults, which was unaffected by demographic or sleep-related factors (12). Moreover, several studies indicated a potential connection between sleep traits and cardiometabolic phenotypes, which represented adverse metabolic conditions (13, 14). While the understanding of the relationship between specific glycemic traits and multiple sleep traits is limited. In addition, there are disadvantages that cannot be avoided. First, the sample sizes of published studies were too small, which weakened the generalizability of the findings. Furthermore, multiple confounding factors, such as physical activity and nutritional status (15, 16), could interfere with interpreting results, creating bias in relevant research. More importantly, these studies often emphasized the presence or absence of association while failing to pay enough attention to the causal relationships because of the significant challenges presented in exploring causality solely through observational studies. Nevertheless, clarifying the causality between glycemic traits and sleep traits would be valuable for investigating the underlying mechanisms and would aid in implementing early preventive interventions for abnormal glucose metabolism and various sleep traits. Therefore, it would be of great value to apply powerful tools to clarify the relationship, causal direction, and effect sizes between sleep traits and glycemic traits.

Meta-analysis, by expanding the sample sizes from multiple centers, has the advantages of avoiding bias from a single study. In addition, Mendelian randomization (MR) analysis is a popular method to clarify the causal association between risk factors and health outcomes in observational epidemiological research by using available genetic variants as instrumental variables (IVs) (17). In contrast to traditional observational studies, MR analysis confronts less latent interference due to the naturalness and randomness of genetic variants in inheritance to offspring. At the same time, MR can avoid the disadvantages of randomized trials, such as their cost, long duration, and infeasibility (18). To date, MR analysis has been successfully applied to the causal research of sleep traits (19–24). Although the publication of the latest genome-wide association studies (GWAS) covering EDS (25), insomnia (26), sleep duration (27), and glycemic traits (28) has provided the basis for the achievability of MR analyses, comprehensive MR analyses on the causality of sleep traits and glycemic traits have not been performed previously.

**Figure 1:**
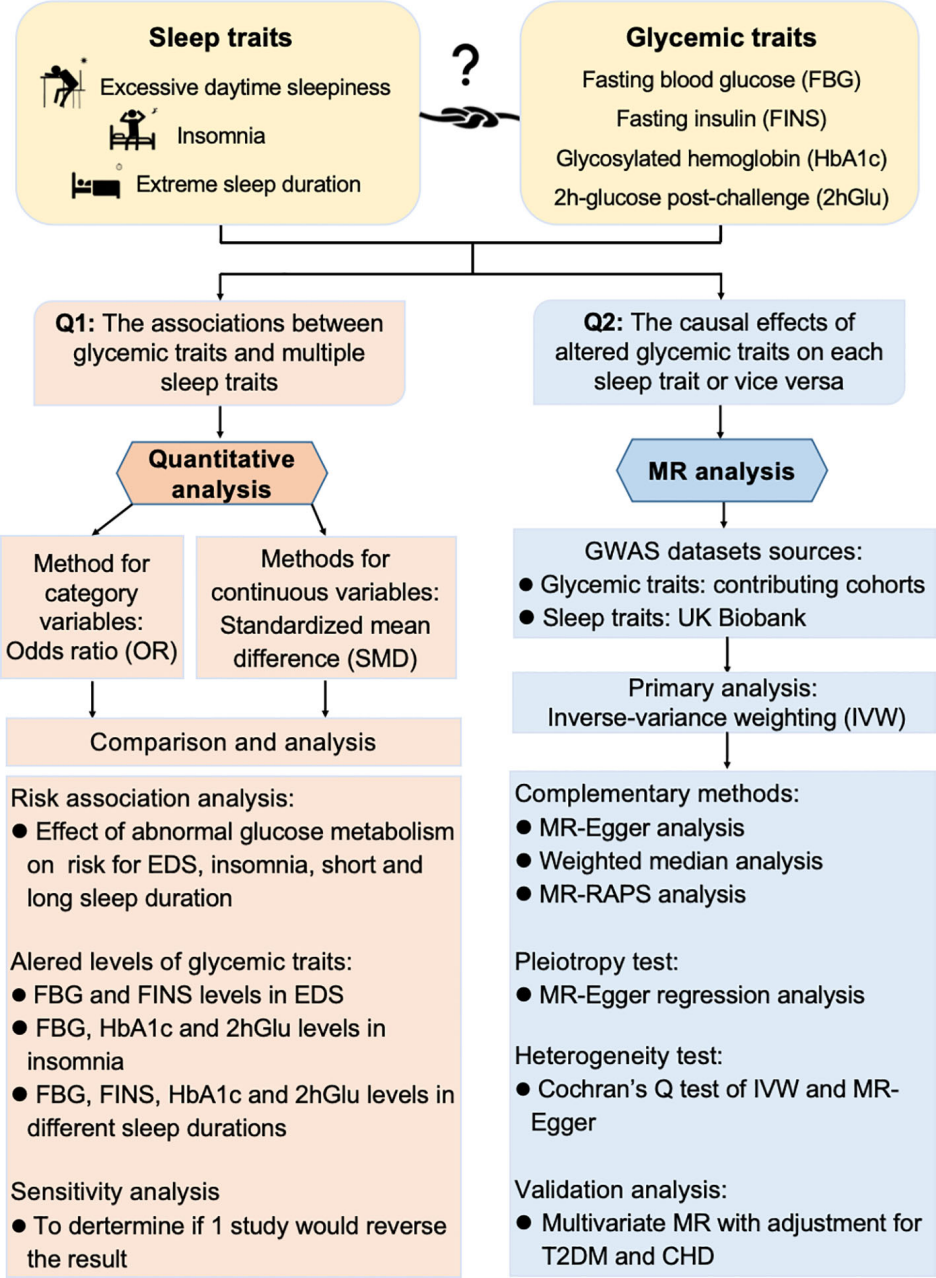
Workflow to study association and causality between glycemic metabolism and sleep traits. Quantitative analysis and Mendelian randomization (MR) analysis were combined to explore the association and causality between sleep traits and glycemic metabolism. OR, odds ratio; SMD, standardized mean difference; EDS, excessive daytime sleepiness; FBG, fasting blood glucose; HbA1c, glycosylated hemoglobin; FINS, fasting insulin; 2hGlu, 2-h glucose post-challenge; IV, instrumental variable; IVW, inverse variance weighting; RAPS, Robust Adjusted Profile Score; CHD, coronary heart disease; T2DM, type 2 diabetes mellitus.

The objective of our study is to assess the associations and causal relationships between sleep traits and glycemic traits. We have pictorially presented the article abstract in **Figure 1**. By combining meta-analysis and MR together, we found an interactive relationship and bidirectional causality between sleep traits and glycemic traits, suggesting potential intervention strategies to enhance the management of glucose metabolism and improve sleep quality.

## Materials and methods

### Quantitative analysis to evaluate the associations

#### Search strategy

PubMed, Web of Science (WOS), Embase, and Cochrane Library were searched on 7 March 2022, and updated on 20 September 2023 using the following terms: one sleep trait in turn (daytime sleepiness, insomnia, and sleep duration) and glycemic traits (glycated hemoglobin OR glycosylated hemoglobin OR HbA1c OR insulin OR glucose OR sugar OR glycemic), which were then connected by “and”.

#### Selection criteria

The listed inclusion and exclusion criteria for each topic strictly adhered to the PICOS principles, i.e., participants (P), intervention (I), control (C), outcome (O), and study design (S).

The criteria to evaluate the impact of altered glucose metabolism on the occurrence of sleep traits were as follows: (1) P: Participants were reported with diabetes, prediabetes, and other abnormal glucose metabolic conditions without restrictions of age, gender, and other demographic characteristics. Abnormal glucose metabolism was determined in accordance with any highly recognized diagnostic criteria, such as guidelines from the American Diabetes Association (ADA), World Health Organization (WHO), and so on. (2) I: There was no intervention involved. (3) C: People without abnormal glucose metabolic conditions served as controls. (4) O: There were recorded events of specific sleep trait in each group. (5) S: Studies provided available data in case–control or cross-sectional configurations.

To assess the level differences in glycemic traits between groups with and without a specific sleep trait, the PICOS were as follows: (1) P: Participants were confirmed with EDS or insomnia without restrictions of age, gender, and other demographic characteristics. EDS was diagnosed when participants obtained an Epworth sleepiness scale (ESS) score of 10 or above. Insomnia was diagnosed based on the Diagnostic and Statistical Manual of Mental Disorders (DSM), the International Classification of Sleep Disorders (ICSD), various standardized questionnaires, or other reliable approaches. (2) I: There was no intervention involved. (3) C: People without the corresponding sleep trait were selected as controls. (4) O: There was an absolute level with the clear unit form for each glycemic trait. (5) S: Studies provided available data in case–control designs or others.

To compute the absolute concentrations of glycemic traits in individuals with different sleep durations, the PICOS were as follows: (1) P: Participants were classified as short (<7 h), normal (7–9 h), or long sleep duration (>9 h) by self-reports or objective measures without restrictions of age, gender, and other demographic characteristics. (2) I and O: They are the same as described above. (3) C: No control group was accounted for the single-arm design. (5) S: Studies provided available data in prospective or cross-sectional configurations.

Additionally, we set the exclusion criteria as follows: unoriginal studies, duplicate publications, and studies focusing on non-human subjects or with missing data.

#### Data extraction and quality assessments

Data collection was carried out separately by two researchers, and controversies were settled by discussion when there were divergences. Accordingly, basic information (first author, country or region, and publication year), sample size and diagnosis detail of participants, events of specific sleep traits, levels of glycemic traits, etc. were extracted, and the Newcastle–Ottawa Scale (NOS) was adopted to perform the quality evaluation of the included literature (29).

#### Statistical analysis

Data analysis was operated in Review Manager 5.3 (The Nordic Cochrane Centre, The Cochrane Collaboration, London, UK). For dichotomous variables, pooled odds ratio (OR) and 95% confidence intervals (95% CI) were calculated to assess the strength of the association. For continuous variables, standardized mean differences (SMDs) and 95% CI were calculated to evaluate the differences in levels of glycemic traits between compared groups. In addition, single-arm meta-analysis was used to compute the absolute levels and 95% CI of glycemic traits in individuals with different sleep behaviors (short, normal, and long sleep duration) in Stata/SE 15.1 for Mac (64-bit Intel) Revision 21 Nov 2017. The data format of mean ± standard deviation (SD) was used for analysis, the mean ± standard error (SE) was transformed using the formula SE = SD/√N (N = number of individuals), while median and interquartile range (IQR) were transformed using Wan et al.’s and Luo et al.’s statistical methods in cooperation with the sample sizes, which have proven to be more adaptive and stable (30, 31). Next, heterogeneity for the articles included in the analysis was monitored by the *I*² statistic. If *I* ^2^ > 50%, the random effect model was implemented to calculate the pooled data; otherwise, the fixed effect model was used. Meanwhile, sensitivity analyses were performed, wherein each study was sequentially removed, and the analysis was repeated to determine if any individual study would reverse the statistical significance of the results. Lastly, publication bias was checked through funnel plots to enhance quality evaluation.

### Mendelian randomization analyses to estimate causality

#### Study design

A two-sample MR analysis was performed to assess the causal relationship between sleep traits and FBG, HbA1c, FINS, and 2hGlu levels, respectively. In detail, we first explored the causal effect of each sleep trait on glucose metabolism traits, and then reversely, the causal influences of glucose metabolism traits on sleep traits were investigated.

#### GWAS datasets

The GWAS summary statistics for glycemic traits of FBG (mmol/L), FINS (pmol/L), HbA1c (%), and 2hGlu (mmol/L) levels were studied from 281,416 non-diabetic participants with different ancestors, including European, Hispanic, East Asian, South Asian, African-American, and sub-Saharan African participants (28). After adjustment for body mass index (BMI), 99 novel loci and 143 previous loci were identified by single-ancestry and trans-ancestry GWAS meta-analyses.

The datasets for sleep traits were all from the UK Biobank, a prospective research program of over 500,000 residents in the UK (32). The majority of participants were of European ancestry and the sleep status of EDS (*N* = 452,071), insomnia (*N* = 453,379), and sleep duration (*N* = 446,118) was from the self-reported questionnaire. To determine EDS, participants were asked “How likely are you to doze off or fall asleep during the daytime when you don’t mean to” and then were divided into cases and controls according to their different answers (25). For insomnia, the question to identify was “Do you have trouble falling asleep at night or do you wake up in the middle of the night?” (26), while sleep duration was defined according to the question “About how many hours of sleep do you get in every 24 h? (including naps), with responses in hour increments” (27). The cutoff for sleep duration was <7 h for short sleepers, 7–9 h for normal sleepers, and ≥7 h for long sleepers. Finally, 42 loci, 57 loci, and 78 loci were identified respectively for EDS, insomnia, and sleep duration.

The GWAS datasets for glycemic traits and sleep traits we chose have larger sample sizes compared with others, and they were all obtained through rigorous methodology. However, the GWAS dataset we chose has two limitations. One is that most of the study population is of European ancestry, and the other is that sleep traits were obtained through self-reports rather than objective assessment.

#### Selection of genetic variants

In the selection of genetic variants, significant genome-wide single-nucleotide polymorphisms (SNPs) were set as *p* < 5×10^−8^. Moreover, criteria of distance = 10,000 kb and *r* ^2^ = 0.001 were enforced to guarantee that selected SNPs associated with sleep traits or glycemic traits were correspondingly independent without linkage disequilibrium. The included instrumental variables (IVs) are presented in **Supplementary eTables 1**, **2**, and their individual and mean *F*-statistics values >10 indicate strong associations between the IVs and each exposure (**Supplementary eTables 1**, **2**; **Supplementary Tables 1**, **2**). In order to estimate the possible effect of the genetic variants on the outcome through probable confounding factors, known as horizontal pleiotropy, we also performed PhenoScanner analysis to screen whether any selected SNP was strongly associated with other traits at a threshold of 5 × 10^−8^ (33, 34). The results of the related traits, effect size (β), standard error (SE), *p* value, and sample size (*n*) for each matched variant were extracted and shown in **Supplementary eTables 3**, **4**.

#### Statistical analysis

We used the inverse variance weighting (IVW) method (35), with all variants assumed to be effective instrumental variables (IV), as our main analysis of the bidirectional causal relationship between sleep traits and glycemic traits. We calculated ORs converted by the exponential β for binary outcomes. The threshold of statistical significance was established as *p*-value <0.05.

To confirm the reliability of the results, a series of sensitivity analyses was performed alongside. If the IVs violated the MR assumptions, MR-Egger (36), weighted median (37), and MR-Robust Adjusted Profile Score (RAPS) (38) were also performed to effectively complement the potential situations. Moreover, intercept values of MR-Egger analysis can reveal potential pleiotropic effects, while Cochrane’s *Q* test was evaluated for heterogeneity in our IVW and MR-Egger analyses. Furthermore, we applied the RadialMR package to identify outliers (39), then repeatedly conducted the above analytical procedure after removing outliers for validation of positive results. In addition, we conducted multivariable MR analysis (MVMR) (40) to estimate the causality of genetically predicted exposure on outcome with adjustment for potential confounders of T2DM (41) and coronary heart disease (CHD) (42).

## Results

### Association between sleep traits and glucose metabolism from quantitative analyses

We finally included a total of 116 publications with a sample size of over 840,000. The inclusive and exclusive procedures for retrieved publications are shown in **Supplementary Figure 1**. Out of these, 26 articles were analyzed to track the occurrence of sleep traits in people with abnormal glucose metabolism, and a further 90 publications were evaluated to highlight the level in differences of glycemic traits between compared groups (**Tables 1**, **2**; **Supplementary Tables 3**, **4**). Every included article was of high quality with an NOS score equal to or more than 6.

**Table 1:** Characteristics of included studies related to sleep traits risk in abnormal glucose metabolism and unaffected population.

Study ID	Country	Case group		Control group		Definition of case	NOS
		Events	Total	Events	Total		
Extensive daytime sleepiness, EDS as events							
Mokhlesi 2019 (43)	US	32	59	18	38	T2DM	7
Hein 2018 (44)	Belgium	134	277	464	1,034	T2DM	8
Mirghani 2016 (45)	KSA	12	178	0	100	T2DM	8
Bediwy 2016 (46)	Egypt	17	45	5	31	FBG ≥ 100 mg/dL	7
Raman 2012 (47)	India	102	1414	5	136	T2DM	8
Reutrakul 2011 (48)	US	11	26	50	116	GDM	7
Skomro 2001 (49)	Canada	32	58	18	48	T2DM	7
Insomnia as events							
Vézina-Im 2021 (50)	Canada	32	54	39	97	T1DM or T2DM	6
Zhang 2019 (51)	China	68	337	578	4,741	T2DM	7
Ramos 2015 (52)	US	60	612	42	401	T2DM	7
Nakanishi-Minami 2012 (53)	Japan	11	74	6	32	T2DM	6
Zheng 2012 (54)	China	113	225	239	773	IFG or IGT	8
Voinescu 2011 (55)	Romania	32	97	16	102	T2DM	7
Vgontzas 2009 (56)	US	41	414	93	1,327	T2DM	8
Short sleep duration (<7 h) as events							
Jang 2023 (57)	Korea	1,183	1,630	5,156	7,186	DM	8
Cui 2021 (58)	China	174	550	207	550	T2DM	7
Lu 2021 (59)	China	136	1,503	1,671	18,999	IFG	6
Liu 2020 (60)	China	128	2,023	1,393	40,781	T2DM	8
Joo 2020 (61)	Korea	150	525	394	1,639	IFG	8
Titova 2020 (62)	Sweden	518	1,523	5,547	17,246	DM	7
Wang 2017 (63)	China	22	919	228	11,587	GDM	8
Lou 2014 (64)	China	145	634	1,839	14,511	IFG	7
Lou 2012 (65)	China	234	954	3,009	15,939	T2DM	7
Chao 2011 (66)	China	47	180	399	2,495	T2DM	6
Wang 2021 (67)	China	17	196	3	304	GDM	6
Long sleep duration (>9 h) as events							
Titova 2020 (62)	Sweden	89	1,523	608	17,246	DM	7
Chojnacki 2018 (68)	Canada	93	996	605	8,433	HbA1c ≥ 6.5%	6
Wang 2017 (63)	China	542	919	6,339	11,587	GDM	8
Raman 2012 (47)	India	23	1,414	1	136	T2DM	6

DM, diabetes mellitus; T2DM, type 2 diabetes mellitus; FBG, fasting blood glucose; IFG, impaired fasting glucose; IGT, impaired glucose tolerance; GDM, gestational diabetes mellitus; HbA1c, glycated hemoglobin; NOS, Newcastle–Ottawa Scale. Events: suffering from the corresponding sleep disturbance.

**Table 2:** Characteristics of the included studies about glycemic traits levels in EDS and insomnia.

Study ID	Country	No. Cases/controls	Level (Cases)	Level (Controls)	NOS
Excessive daytime sleepiness, EDS as case group					
*Fasting blood glucose, FBG*					
Li 2019 (15)	China	33/24	5.28 ± 0.69	5.43 ± 0.72	6
Huang 2016# (69)	China	119/56	5.45 ± 0.66	5.28 ± 0.60	7
Andaku 2015 (70)	Brazil	27/308	96.6 ± 16.3	98.3 ± 23.7	7
Yu 2015 (71)	Korea	14/11	92.71 ± 1.18	93.09 ± 9.58	8
Pulixi 2014 (72)	Italy	13/39	99 ± 13	104 ± 29	6
Bonsignore 2012 (73)	Italy	25/25	102.9 ± 16.9	94.1 ± 13.1	7
Nena 2012 (74)	Greece	274/255	108.4 ± 15.2	105.8 ± 13.0	7
Barcelo 2008 (16)	Spain	22/22	115 ± 19	103 ± 20	8
*Fasting insulin, FINS*					
Li 2019 (15)	China	33/24	15.83 ± 11.05	10.50 ± 4.95	6
Huang 2016# (69)	China	119/56	97.36 ± 48.53	76.70 ± 29.95	7
Nena 2012 (74)	Greece	25/25	19.7 ± 14.3	11.5 ± 5.6	7
Barcelo 2008 (16)	Spain	22/22	15.2 ± 7.6	8.6 ± 4.8	8
Insomnia as case group					
*Fasting blood glucose, FBG*					
O 2023 (75)	China	90/896	7.93 ± 2.53	7.40 ± 2.23	8
Zhang 2021 (76)	China	94/178	4.73 ± 0.77	4.74 ± 1.19	8
Xu 2020 (77)	China	30/18	5.2 ± 1.1	5.1 ± 1.2	8
Leblanc 2018 (78)	US	16,714/33,729	102.4 ± 9.45	102.4 ± 9.70	6
Tschepp 2017 (79)	Germany	17/15	97.6 ± 8.2	96.3 ± 10.3	8
Ham 2017 (80)	Korea	106/307	97.07 ± 12.17	98.28 ± 18.66	7
Pyykkönen 2012 (81)	Finland	163/395	5.6 ± 0.5	5.5 ± 0.5	8
Keckeis 2010 (82)	Germany	21/33	98.8 ± 8.9	96.0 ± 8.0	7
*Glycosylated hemoglobin, HbA1c*					
O 2023 (75)	China	90/896	7.84 ± 1.66	7.40 ± 1.29	8
Leblanc 2018 (78)	US	12,485/32,776	5.8 ± 0.26	5.9 ± 0.25	6
Tschepp 2017 (79)	Germany	17/15	5.3 ± 0.3	5.3 ± 0.3	8
Keckeis 2010 (82)	Germany	21/33	5.3 ± 0.3	5.2 ± 0.3	7
*2-h glucose post-challenge, 2hGlu*					
Tschepp 2017 (79)	Germany	17/15	100.5 ± 14.8	105.1 ± 28.7	8
Pyykkönen 2012 (81)	Finland	163/395	5.7 ± 1.6	5.2 ± 1.4	8
Keckeis 2010 (82)	Germany	21/33	120.2 ± 17.8	109.5 ± 23.5	7

Data are expressed as mean ± SD unless specifically labeled. *Data are expressed as means ± SE; #Data are expressed as median (interquartile). NO: Number; NOS: Newcastle–Ottawa Scale.

#### The risks of sleep traits increase in abnormal glucose metabolism population

**Figure 2:**
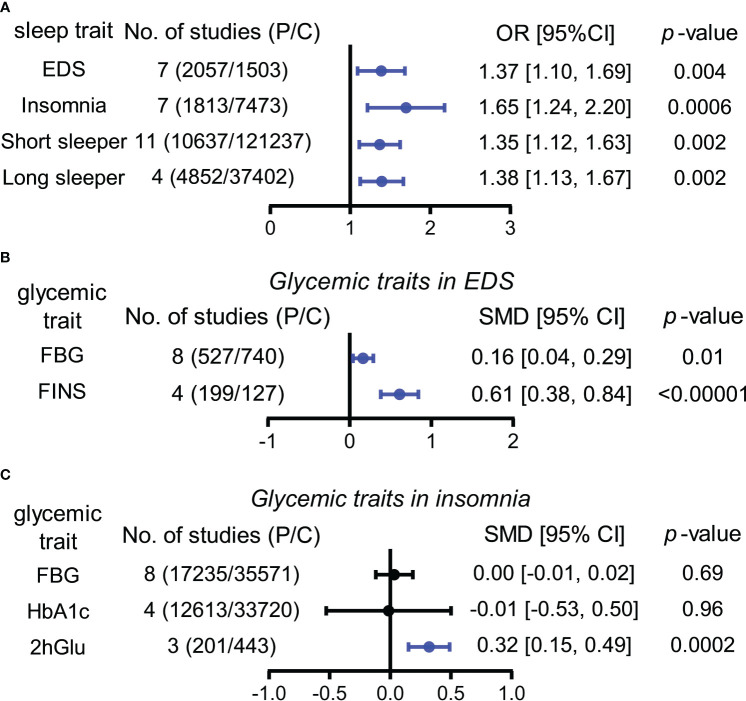
Associations between sleep traits and glucose metabolism by meta-analysis. **(A)** OR (odds ratio) and 95% confidence interval (95% CI) were used to assess the effects of abnormal glucose metabolism on the risk of EDS, insomnia, and short and long sleep duration. **(B)** Standardized mean difference (SMD) and 95% CI were used to compare the level differences of glycemic traits (FBG and FINS) between participants with and without EDS. **(C)** Standardized mean difference (SMD) and 95% CI were used to compare the level differences of glycemic traits (FBG, HbA1c, and 2hGlu) between participants with and without insomnia. FBG levels associated with insomnia in Panel **(C)** (bottom) show the result after multiplying the original value by 10. The statistically different results with *p* < 0.05 are shown in blue. EDS, excessive daytime sleepiness; dur, duration; NO, number; P/C, patients/controls; FBG, fasting blood glucose; HbA1c, glycosylated hemoglobin; FINS, fasting insulin; 2hGlu, 2-h glucose post-challenge.

For this section, the abnormal glucose metabolism for participants in the analyses encompassed diabetes and impaired fasting glucose. We observed that participants with abnormal glucose metabolism had higher increases in the risks for four sleep traits compared with the unaffected population, including EDS (37%), insomnia (65%), and short (35%) and long (38%) sleep duration (**Table 1**; **Figure 2A**; **Supplementary Figure 2**). Sensitivity analysis confirmed the robustness of the results, and funnel plots suggested no publication bias (**Supplementary Figure 3**).

#### EDS and insomnia influence levels of glycemic traits differently

According to our analyses, participants suffered from higher FBG (SMD = 0.16, 95% CI = [0.04,0.29]) and FINS (SMD = 0.61, 95% CI = [0.38,0.84]) levels in comparison with controls, while insomnia participants suffered from higher 2hGlu (SMD = 0.32, 95% CI = [0.15,0.49]) levels. There was a lack of association between FBG and HbA1c levels and insomnia (**Figures 2B, C**; **Supplementary Figure 4**). Sensitivity analysis demonstrated that there were limited articles that would contradict the above statistical findings. Funnel plots suggested no publication bias in meta-analyses related to EDS and insomnia (**Supplementary Figure 5**).

#### FINS and HbA1c show associations with sleep duration

**Figure 3:**
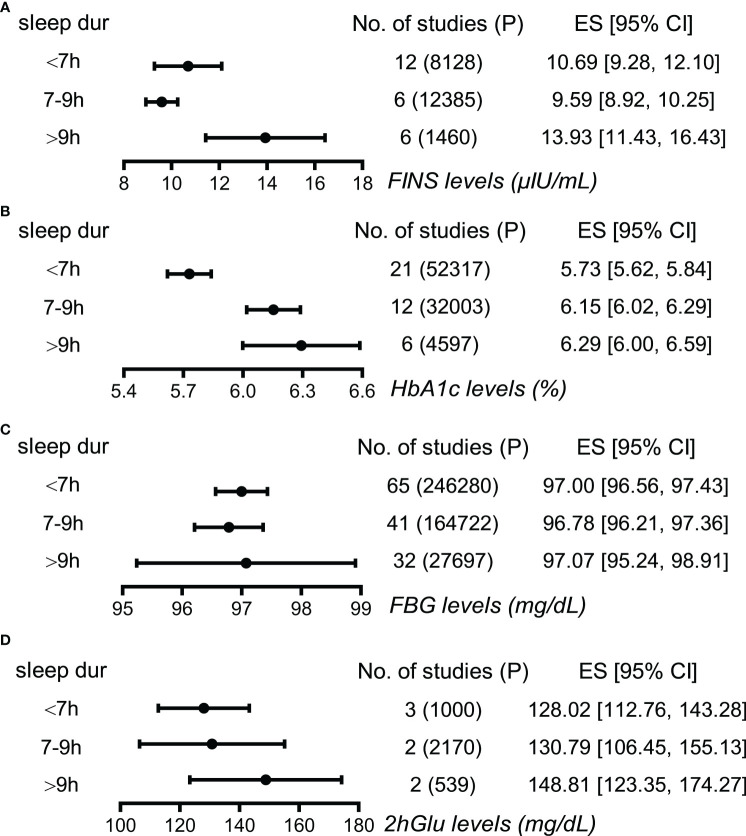
Associations between sleep duration and glucose metabolism by meta-analysis. **(A)** Effect size (ES) and 95% confidence interval (95% CI) were used to assess the levels of FINS in short (<7 h), normal (7–9 h), and long sleep duration (>9 h). **(B)** ES and 95% CI were used to assess the levels of HbA1c in short (<7 h), normal (7-9 h), and long sleep duration (>9 h). **(C)** ES and 95% CI were used to assess the levels of FBG in short (<7 h), normal (7–9 h), and long sleep duration (>9 h). **(D)** ES and 95% CI were used to assess the levels of 2hGlu in short (<7 h), normal (7–9 h), and long sleep duration (>9 h). P, participants; FBG, fasting blood glucose; HbA1c, glycosylated hemoglobin; FINS, fasting insulin; 2hGlu, 2-h glucose post-challenge; NO, number; sleep dur, sleep duration.

Next, long sleepers and short sleepers revealed significant and mild higher levels of FINS compared to normal sleepers, demonstrating a J-shape association between FINS levels and sleep duration (**Figure 3**; **Supplementary Figure 6**). Furthermore, HbA1c levels were higher in long sleepers than in normal sleepers, who in turn had higher HbA1c levels than short sleepers, exhibiting a tendency to increase roughly with prolonged sleep duration (**Figure 3**; **Supplementary Figure 7**). However, we failed to find apparent differences in FBG levels and 2hGlu levels among people with different sleep durations (**Figure 3**; **Supplementary Figures 8**, **9**). Funnel plots indicated no publication bias in all the single-arm meta-analyses (**Supplementary Figures 10–13**).

### Causalities exist between sleep traits and glycemic traits by Mendelian randomization

#### Elevated 2hGlu level has a probable causal effect on higher EDS risk

**Figure 4:**
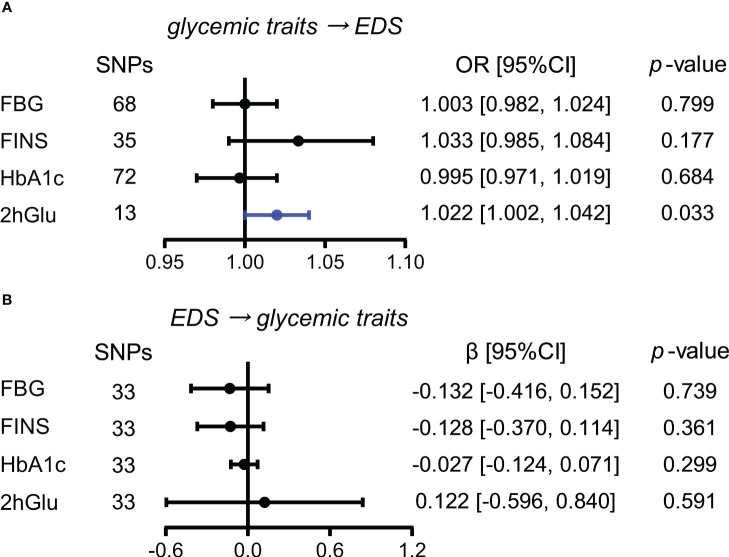
Assessment of causal associations between glucose metabolism and EDS. **(A)** Causal effects of glycemic traits (FBG, FINS, HbA1c, and 2hGlu levels) on EDS were estimated by the primary method of IVW. **(B)** Causal effects of EDS on FBG, FINS, HbA1c, and 2hGlu levels were estimated by the primary method of IVW. The statistically different results with *p* < 0.05 are shown in blue. EDS, excessive daytime sleepiness; SNP, single-nucleotide polymorphism; OR, odds ratios; β: regression coefficient; CI, confidence intervals; FBG, fasting blood glucose; FINS, fasting insulin; HbA1c, glycosylated hemoglobin; 2hGlu, 2-h glucose post-challenge.

When estimating the causality of altered levels of glycemic traits on EDS by the primary method of IVW, a slight causal effect of 2hGlu levels on EDS was observed (OR = 1.022, 95% CI = [1.002,1.042], *p* = 0.033), with each 1 unit change in 2hGlu levels increasing 2.2% in EDS (**Figure 4A**). No pleiotropy in MR-Egger analysis was found, but both IVW and MR-Egger showed the presence of heterogeneity (**Supplementary Table 5**). Next, after outliers were removed, the significant association of 2hGlu levels on EDS was further confirmed in more detailed methods such as IVW, weighted median, and MR RAPS (**Supplementary Figure 14**). While the causality from EDS to glycemic traits was null (**Figure 4B**).

#### Increased 2hGlu level has a probable causal effect on higher risk for insomnia

**Figure 5:**
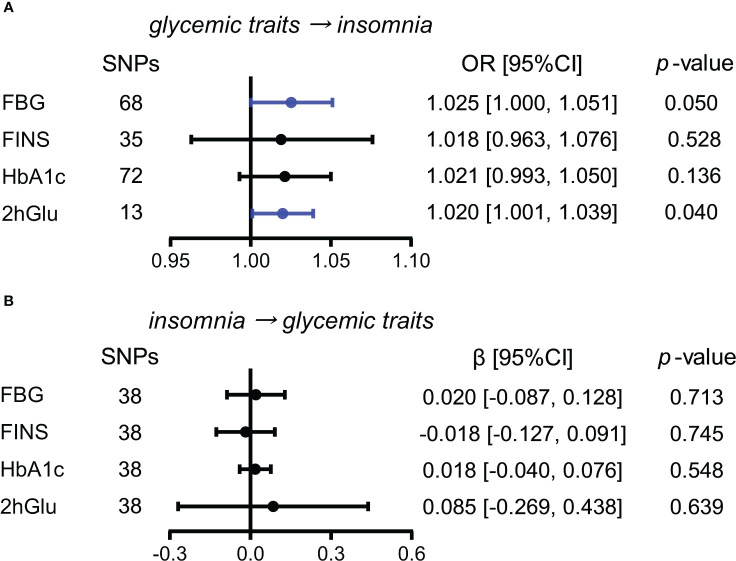
Assessment of causal association between glucose metabolism and insomnia. **(A)** Causal effects of glycemic traits (FBG, FINS, HbA1c, and 2hGlu levels) on insomnia were estimated by the primary method of IVW. **(B)** Causal effects of insomnia on FBG, FINS, HbA1c, and 2hGlu levels were estimated by the primary method of IVW. The statistically different results with *p* < 0.05 are shown in blue. SNP, single-nucleotide polymorphism; OR, odds ratios; β: regression coefficient; CI, confidence intervals; IVW, inverse variance weighting; FBG, fasting blood glucose; FINS, fasting insulin; HbA1c, glycosylated hemoglobin; 2hGlu, 2-h glucose post-challenge.

Next, we conducted similar analyses between four glycemic traits and insomnia. By the IVW method, we observed the almost causal effect of FBG levels (OR = 1.025, 95% CI = [1.000,1.051], *p* = 0.050) on insomnia and significant causal influence of 2hGlu level on insomnia (OR = 1.020, 95% CI = [1.001,1.039], *p* = 0.040) (**Figure 5A**). Moreover, the effects of FBG and 2hGlu levels on insomnia were replicated by different complemented methods after excluding outliers (**Supplementary Figures 15A, B**). No significant associations were detected from insomnia to analyzed glycemic traits (**Figure 5B**).

#### Higher FINS level is a probable causal factor for increase in short sleep duration

**Figure 6:**
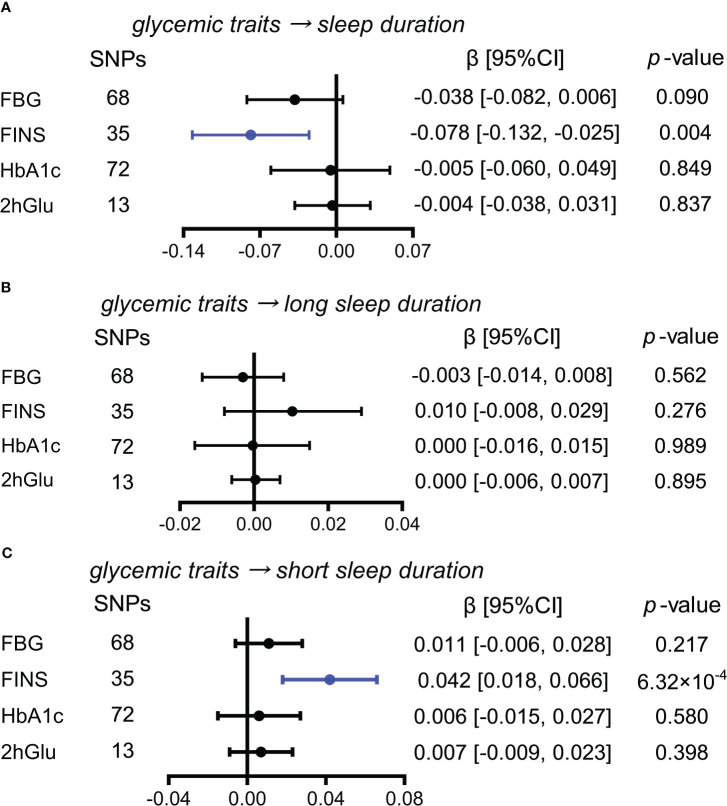
Estimated causal effects of glucose metabolism on sleep duration. **(A–C)** Causal effects of glycemic traits (FBG, FINS, HbA1c, and 2hGlu levels) on sleep duration **(A)**, long sleep duration **(B)**, and short sleep duration **(C)** were estimated by the primary method of IVW. SNP, single-nucleotide polymorphism; β: regression coefficient; CI, confidence intervals; IVW, inverse variance weighting; FBG, fasting blood glucose; FINS, fasting insulin; HbA1c, glycosylated hemoglobin; 2hGlu, 2-h glucose post-challenge.

While exploring the effects of glycemic traits on different sleep duration variables, we found that FINS levels presented causal associations with sleep duration and short sleep duration by IVW (**Figures 6A–C**). Furthermore, in sensitivity analysis, the effect of FINS on sleep duration was weak, as only the method of MR RAPS was significant (**Supplementary Figure 16A**), but probable causality of higher FINS on increases in short sleep duration was observed in other more detailed methods, including weighted median and MR RAPS (**Supplementary Figure 16B**).

#### More short sleep duration has a probable causal effect on HbA1c levels

**Figure 7:**
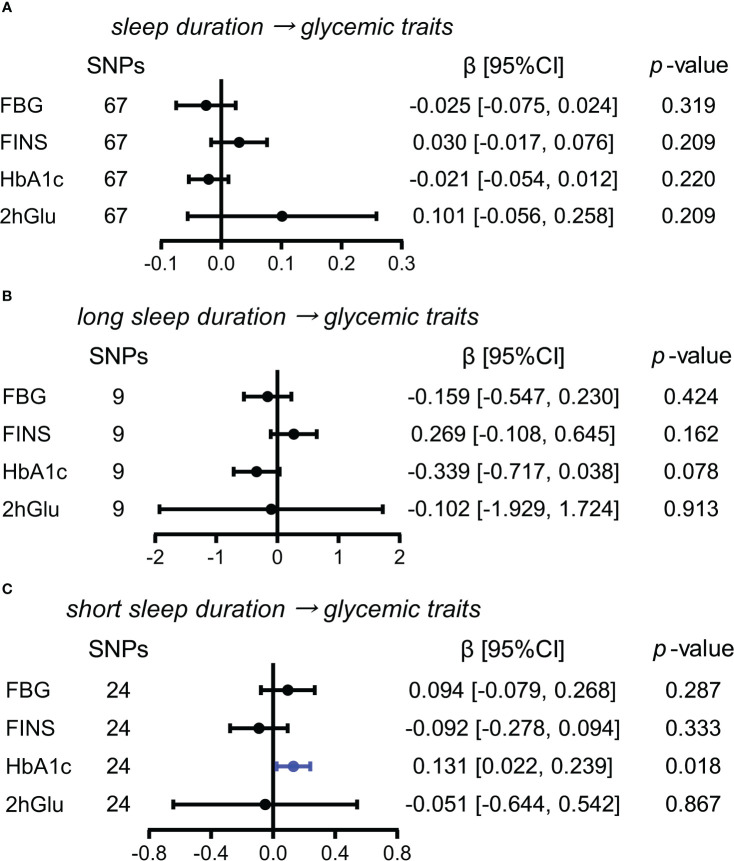
Estimated causal effects of sleep duration on glucose metabolism. **(A–C)** Causal effects of sleep duration **(A)**, long sleep duration **(B)**, and short sleep duration **(C)** on glycemic traits (FBG, FINS, HbA1c, and 2hGlu levels) were estimated by the primary method of IVW. SNP, single-nucleotide polymorphism; β: regression coefficient; CI, confidence intervals; IVW, inverse variance weighting; FBG, fasting blood glucose; FINS, fasting insulin; HbA1c, glycosylated hemoglobin; 2hGlu, 2-h glucose post-challenge.

In exploring the effects of sleep duration, and short and long sleep duration as causes for glycemic metabolism, we only observed the causal influence of an increase in short sleep duration on HbA1c levels using the IVW method (β = 0.131, 95% CI = [0.022,0.239], *p* = 0.018) (**Figures 7A–C**). The causal influence of short sleep duration on HbA1c levels was also supported by weighted median and MR RAPS except for MR-Egger (**Supplementary Figure 17**). Thus, the causal effect of short sleep duration on HbA1c levels was probable.

#### Results of MVMR to test the effect of potential confounding factors

Furthermore, MVMR results showed that the causal effects of 2hGlu levels on EDS and insomnia were robust after adjustment for CHD. In addition, the causality of 2hGlu levels on EDS instead of insomnia remained after adjustment for T2DM. Furthermore, the effect of FINS on short sleep duration was consistent when adjusting for CHD but was eliminated upon adjustment for T2DM, although the effect size showed the same direction as the original effect size (**Supplementary Table 6**). Lastly, the causal association from short sleep duration to HbA1c levels disappeared with CHD and T2DM adjusted. Taken together, our MR results above indicated that minimal pleiotropic SNPs existed and supported the observed causalities.

## Discussion

The relationship between glucose metabolism and sleep has been a widely debated and poorly understood topic. In this study, we conducted a comprehensive meta-analysis and MR analysis to explore the relationship between five sleep traits and four distinct glycemic traits. Our analyses demonstrated that there are mutual risk associations between glycemic traits and several sleep traits. We also observed that probable causalities exist mainly from disturbed glycemic traits to sleep disruption. These findings offer valuable perspectives in the early recognition and prevention of glucose disruption and the sleep problems, and emphasize the importance of further research to elucidate the underlying mechanisms.

EDS and insomnia are two common sleep disorders; however, their relationships with glucose metabolism remained unclear. In our analyses, we found that individuals with abnormal glucose metabolism are inclined to have sleepiness and insomnia, and identified that the EDS and insomnia population tended to have higher FBG and FINS levels, and higher 2hGlu levels, respectively. These results were supported by several observation studies (12, 43, 83). Furthermore, our MR analyses expanded these findings by demonstrating the probable causation of increasing 2hGlu levels on EDS and insomnia risk in the non-diabetic population. This is complementary to previous analyses, which reported that no significant causal effect of T2DM on insomnia was found (84), or that insulin resistance does not contribute to the development of insomnia (85). Some studies have improved our understanding of pathophysiologic pathways that underpin the probable causality. Hyperglycemia was found to enhance an increase in tryptophan, which plays an essential role in promoting sleep by influencing the serotonin synthesis in the central nervous system (86). This evidence appears to be more plausible and suggests that investigating the role of tryptophan as a rewarding aspect in the causal pathway from elevated 2hGlu levels to EDS is warranted. Moreover, several mechanistic studies have demonstrated that increasing glucose levels can activate the RAF-MEK-ERK-NF-κB pathway, resulting in inflammation and subsequently contributing to the development of insomnia (87, 88). Nocturnal polyuria due to osmotic diuresis and neuralgia due to peripheral neuropathy may also be potential contributors to insomnia (89). Additionally, our findings highlighted the potential health threat of extremely increasing glucose levels or even the pre-diabetic stage of the impairment of glucose tolerance to EDS and insomnia, underscoring the importance of eliminating overeating and managing postprandial glucose levels to alleviate sleep quality.

Furthermore, we found that there was a J-shaped relationship between sleep duration and FINS levels, and a rising trend in HbA1c levels from short to long sleep duration. The non-linear associations were similar to other J/U-shaped associations between sleep duration with blood pressure (90, 91), and between sleep duration with cognitive impairment (92, 93). Furthermore, our results from bidirectional MR analyses demonstrated potential causal effects of FINS levels on short sleep duration, and short sleep duration on HbA1c levels, suggesting a vicious cycle between short sleep duration and glucose metabolism disruption. However, no causation was revealed between long sleep duration and FINS levels by our MR analysis. The potential mechanisms that connect sleep duration and glucose metabolism may vary between short and long sleep duration. Central insulin signaling in glucose metabolism plays a crucial role in regulating sleep-related mechanisms and sleep architecture, which makes it possible to promote short sleep duration (94, 95). Additionally, short sleep duration may decrease melatonin secretion (96) and trigger systematic inflammation (97, 98), potentially disrupting glycemic control through a variety of pathways, including insulin resistance, reduced insulin sensitivity, and impaired glucose tolerance (99). Moreover, short sleep duration can lead to decreased leptin levels and increased growth hormone-releasing peptide levels, which can result in an increase in hunger and appetite, adding to the burden of glucose metabolic regulation (100). Currently, the potential mechanisms that explain the relationship between longer sleep duration and an increased risk of diabetes are still largely speculative. Long sleep duration is suggested to potentially be associated with several risk factors that can disrupt glucose metabolism, such as fatigue, depression, obstructive sleep apnea, undiagnosed medical disease, low socioeconomic status, low physical activity, and poor physical health (101).

As far as we know, our study is the first to involve both associations and bidirectional causations between sleep traits and glycemic traits, and shows the advantages of larger sample sizes, fewer potential confounding factors, higher cost-effective ratios, etc. We identified detailed potential causal pathways linking various sleep traits (EDS, insomnia, and sleep duration) and glycemic traits (FBG, FINS, 2hGlu, and HbA1c), addressing gaps in existing studies and expanding the scope of relevant research. Notably, our bidirectional analysis of glycemic traits and sleep traits revealed the presence of a potential feedback loop. This feedback loop establishes a dynamic and self-reinforcing relationship between altered levels of glycemic traits and short sleep duration, ultimately contributing to adverse outcomes. At the same time, our bidirectional approach overcomes the interpretation challenges associated with reverse causation in traditional observational studies, mainly the inability to determine which is the exposure and which is the outcome of two variables.

Moreover, our findings have significant clinical implications. Firstly, we have revealed that alterations in specific glycemic traits could cause adverse effects on sleep even in populations without diabetes. Our findings highlight the significance of monitoring alteration in glycemic traits before the development of diabetes and propose the possibility of developing potential schemes for the early recognition of DM based on altered levels of glycemic traits. For instance, considering the causal effect of short sleep duration on HbA1c levels in non-diabetic populations as well as the ease of measuring sleep duration, the development of appropriate clinical screening of sleep behavior could prove advantageous in identifying populations with or at risk of diabetes as early as possible. Secondly, our results suggest potential clinical strategies to mitigate outcomes by modifying exposures, as both glycemic traits and sleep traits are modifiable factors. For example, considering the causality from FINS levels to short sleep duration and the causality from short sleep duration to HbA1c levels, regulating FINS levels would help to ensure adequate sleep duration, and health education on avoiding short sleep duration could improve long-term regulation of glucose metabolism, thereby breaking the vicious cycle between altered levels of glycemic traits and short sleep duration. A randomized controlled trial demonstrated that educational intervention for patients with T2DM or impaired fasting blood glucose effectively enhanced sleep quantity and quality while lowering FBG levels and HbA1c levels within a 3-month period, confirming the clinical value of sleep education (102).

However, there are still several unavoidable limitations in our study. First, some variables included a relatively small number of publications in the meta-analysis, which may lead to an publication bias. Second, the definitions of sleep traits in GWAS summary statistics were based on self-reporting, which may be subject to classification bias. Third, participants in the GWAS were mainly recruited from groups of European ancestry, which limited the applicability of our results to those with non-European ancestry. Moreover, the objective monitoring methods of sleep traits and the inclusion of a wider range of genetic backgrounds within the study population should also be considered. Furthermore, conducting more mechanistic studies involving biochemical pathways could help elucidate the causal link between sleep traits and glucose metabolism.

In conclusion, our results suggest important and close associations between abnormal glucose metabolism and multiple sleep traits. Additionally, we found probable causal associations from higher 2hGlu levels to more EDS and insomnia, from elevated FINS levels to short sleep duration, and from increasing short sleep duration to higher HbA1c levels. In the future, studies are encouraged to elucidate the underlying mechanisms and develop strategies to improve sleep and prevent disruptions in glucose metabolism and adverse outcomes.

## Data availability statement

The original contributions presented in the study are included in the article/**Supplementary Material**. Further inquiries can be directed to the corresponding author.

## Author contributions

Study design: YZ and MY; data collection: YZ, QF, ZC, WZ, KL, SJ, BL, MH, XS, and MY; data analysis: YZ, QF, ZC, WZ, KL, SJ, BL, MH, XS, and MY; writing: YZ, QF, ZC, WZ, KL, SJ, BL, MH, XS, and MY; funding: YZ and MY; administration: YZ and MY. All authors contributed to the article and approved the context of the article.

## Supplementary material

Data Sheet 1 **Supplemental Table 1–6** and **Supplemental Figure 1–17**.

Data Sheet 2
**Supplemental eTable 1–4**.
