# Supplementary material for: Unraveling the associations and causalities between glucose metabolism and multiple sleep traits
Source: Front Endocrinol (Lausanne). 2023 Nov 7;14:1227372. doi: 10.3389/fendo.2023.1227372 (PMC10660979; doi:10.3389/fendo.2023.1227372)
Supplement: Data Sheet 1 — Supplemental Table 1–6 and Supplemental Figure 1–17. [file DataSheet_1.pdf]

Supplementary Material

1.1 Supplemental Tables

Supplemental Table 1. Summary of mean F-statistic for each glycemic trait and selected genetic variants.

Exposure	Outcome									
	Daytime sleepiness		Insomnia		Sleep duration		Short sleep duration		Long sleep duration	
	nSNPs	F-statistic(mean)	nSNPs	F-statistic(mean)	nSNPs	F-statistic(mean)	nSNPs	F-statistic(mean)	nSNPs	F-statistic(mean)
FBG	68	121.77	68	121.77	68	121.77	68	121.77	68	121.77
FINS	35	52.1	35	52.1	35	52.1	35	52.1	35	52.1
HbA1c	72	103.35	72	103.35	72	103.35	72	103.35	72	103.35
2hGlu	13	58.18	13	58.18	13	58.18	13	58.18	13	58.18

Abbreviation: SNP: Single nucleotide polymorphism; FBG: Fasting blood glucose; FINS: Fasting insulin; HbA1c: Glycosylated hemoglobin; 2hGlu: 2h-glucose post-challenge.

Supplemental Table 2. Summary of mean F-statistic for each sleep trait and selected genetic variants.

Exposure	Outcome							
	FBG		FINS		HbA1c		2hGlu	
	nSNPs	F-statistic(mean)	nSNPs	F-statistic(mean)	nSNPs	F-statistic(mean)	nSNPs	F-statistic(mean)
Daytime sleepiness	33	42.45	33	42.45	33	42.45	33	42.45
Insomnia	38	42.74	38	42.74	38	42.74	38	42.74
Sleep duration	67	40.99	67	40.99	67	40.99	67	40.99
Short sleep duration	24	37.58	24	37.58	24	37.58	24	37.58
Long sleep duration	9	39.76	9	39.76	9	39.76	9	39.76

Abbreviation: SNP: Single nucleotide polymorphism; FBG: Fasting blood glucose; FINS: Fasting insulin; HbA1c: Glycosylated hemoglobin; 2hGlu: 2h-glucose post-challenge.

Supplemental table 3. Characteristics of the included literature in the meta-analysis of EDS or insomnia associated with glycemic traits.

Study ID	PMID	No. total	Research domain	Definition of sleep trait
**Extensive daytime sleepiness, EDS**				
Li 2019[1]	31801522	57	FBG, FINS	ESS score ≥10
Huang 2016[2]	25628156	175	FBG, FINS	ESS score ≥10
Andaku 2015[3]	25586501	25	FBG	ESS score ≥10
Yu 2015[4]	26552460	335	FBG	ESS score ≥11
Pulixi 2014[5]	24763757	52	FBG	ESS score ≥10
Bonsignore 2012[6]	22075482	529	FBG	ESS score ≥10
Nena 2012[7]	21207173	50	FBG, FINS	ESS score ≥11
Barcelo 2008[8]	18535117	44	FBG, FINS	ESS >10 and MSLT <5 min
**Insomnia**				
O 2023[9]	37716256	986	FBG, HbA1c	ISI ≥14
Zhang 2021[10]	34022493	272	FBG	PSQI >7
Xu 2020[11]	32052947	48	FBG	DSM-5
Leblanc 2018[12]	30687505	81233	FBG, HbA1c	Physician-entered diagnosis
Tschepp 2017[13]	28701993	32	FBG, HbA1c, 2hGlu	DSM-4
Ham 2017[14]	28470969	413	FBG	ISI ≥10
Pyykkönen 2012[15]	22837368	558	FBG, 2hGlu	Basic Nordic Sleep Questionnaire
Keckeis 2010[16]	20209158	54	FBG, HbA1c, 2hGlu	International Classification of Sleep Disorders

Abbreviation: NO: Number; total: both cases and controls; FBG: Fasting blood glucose; FINS: Fasting insulin; HbA1c: Glycosylated hemoglobin; 2hGlu: 2h-glucose post-challenge; ESS: Epworth Sleepiness Scale; MSLT: Multiple Sleep Latency Test; PSQI: Pittsburgh Sleep Quality Index; DSM: Diagnostic and Statistical Manual of Mental Disorders; ISI: Insomnia Severity Index.

Supplemental table 4. Characteristics of the included literature related to sleep duration in the meta-analysis of sleep duration associated with glycemic traits.

Study ID	Country	PMID	No. participants	Levels of glycemic traits	Sleep duration (hours)	NOS
**Short sleep duration (<7h)**						
Fasting blood glucose (FBG), mg/dL						
Abe 2011[17]	Japan	21411100	687	99.9±15.12	6	7
			226	102.24±21.6	≤5	
Adachi 2023[18]	Japan	36792215	126	97.8±21.9	<6	8
			277	95.8±19.6	6-7	
Aziz 2017[19]	US	28026999	802	97.6±25.6	<6	8
Bain 2017*[20]	US	28856879	15	90±7.02	<7	8
Brady 2018[21]	UK	29526681	131	95.4±12.6	≤5.5	6
			476	95.4±14.4	6-6.5	
Brocato 2015[22]	US	26621514	292	127±43.1	<7	7
Chang 2015[23]	China	2629202	334	100.06±26.37	6-6.9	8
			60	100.55±27.38	<5	
			175	102.33±33.44	5-5.9	
Chang 2019[24]	China	30557878	4711	99.6±23.4	4-6	7
			179	101.9±25.2	<4	
Chaput 2013[25]	Canada	24099879	38	97.92±20.7	≤6	7
Chaudhry 2023 [26]	US	36839404	453	87.1±10.7	<7	6
Choi 2008[27]	Korea	18475274	481	96.3±0.9	≤5	6
			1084	96.3±0.54	6	
Choi 2011[28]	Korea	22001675	27	100.41±18.46	<6	6
Choi 2017[29]-female	Korea	28192891	174	96.2±20	6-7	6
			83	100.1±27	<6	
Choi 2017[29]-male	Korea	28192891	127	100.4±23.8	6-7	
			47	98.4±28.7	<6	
Chou 2020[30]	China	32941519	2486	90±20	<7	7
D'Aurea 2015[31]	Brazil	26083888	7	102.28±4.5	<5	8
Deng 2017[32]	China	28977563	30092	97.2±16.2	<6	6
Feng 2016*[33]	China	27966597	114	87.84±13.5	≤7	6
Fernandez-Mendoza 2017[34]	US	28515112	695	105.8±32.5	<6	7
Flint 2007[35]	US	17382111	14	84.9±5	≤6	8
Hayes 2011[36]	US	21286230	139	97.5±23.4	5-6	7
			205	98.4±28.8	6-7	
			82	101.4±25.1	<5	
Huang 2014[37]	China	25056969	346	93.6±11.34	<6	7
Im 2017[38]	Korea	28906359	3651	100±27	≤5	8
Javaheri 2011#[39]	US	21146189	61	87.9±7.1	≤6.5	7

Jee 2017*[40]	Korea	27473575	347	142±63.33	<5	7
Kadono 2007[41]	Japan	17969468	554	99±14.9	5-6	7
			1273	100±13.4	6-7	
			133	101.3±18.3	≤5	
Kanagasabai 2017[42]	Canada	28526258	934	108±36	5	8
			2020	108±36	6	
			141	111.6±43.2	≤3	
			389	113.4±39.6	4	
Kaneita 2008[43]	Japan	18517035	70	101.6±26.6	<5	8
			596	104.5±29.5	6-7	
			318	104.8±32.3	5-6	
Katano 2011[44]	Japan	24843515	1690	95±18.7	6-7	7
			642	96.2±19.1	<6	
Kim 2013[45]	Korea	23578884	16078	95.7±13	6-7	8
			5810	96.1±15.3	≤5	
			16155	96.2±14.1	5-6	
Kim 2015[46]	Korea	26359509	11819	98.8±16.5	6	7
			4564	99±17.2	≤5	
Li 2015[47]	China	26116460	1067	83.16±13.14	6-7	6
			1099	85.68±26.46	<6	
Li 2018(A)[48]	China	29415800	18	91.8±7.2	≤7	7
Li 2018(B)[49]	China	30408698	2665	91.62±31.5	<7	7
Lin 2016(A)[50]	China	26922430	444	107.9±32.7	5-7	7
Lin 2016(B)[51]	China	26536829	93	116.8±50.2	≤5	7
Lin 2017[52]	China	29084786	255	94.8±19.5	6-7	8
			100	98.2±28.2	<6	
Lin 2018[53]	China	28238100	334	104.4±25.2	<6	7
			920	104.4±27	6-7	
Lin 2021[54]	China	34065152	544	102.6±14.4	≤4	6
			897	102.6±12.6	4-6	
Liu 2016[55]	China	27327959	613	102.6±21.6	6-7	7
			96	104.4±27	<6	
Marshall 2010[56]	Australia	10.1111/j.1479-8425.2010.00431.x	297	88.74±25.74	6	8
			134	91.44±33.66	≤5	
Matsuo 2022[57]-female	Japan	35319079	1125	91±9	5-6	7
			1965	91±10	6-7	
			238	93±15	<5	
Matsuo 2022[57]-male	Japan	35319079	1277	99±18	5-6	
			2857	100±18	6-7	
			261	104±31	<5	
Min 2016[58]	Korea	27956898	2048	91.5±13.3	6	8
			1108	92.6±15	≤5	
Niijima 2016[59]	Japan	27151211	716	107±27	<6	6

Okamura 2019[60]-female	Japan	30851175	1039	90±7.2	≤5	8
			2733	90±9	5-6	
			2128	90±7.2	6-7	
Okamura 2019[60]-male	Japan	30851175	2377	95.4±10.8	5-6	
			2068	95.4±10.8	6-7	
			889	97.2±14.4	≤5	
Park 2018[61]	Korea	30092896	66786	96.1±15.4	<7	7
Patel 2012[62]-female	India	23734436	5	86.4±8.5	<7	7
Patel 2012[62]-male	India	23734436	33	84.4±7.4	<7	
Prakaschandra 2023[63]	South Africa	37092708	252	6.63±2.87	<6	7
Rafalson 2010[64]	US	20620078	25	91±5.3	<6	8
Ryu 2015*[65]	Korea	25573816	3325	96.12±31.14	≤6	7
Satoh 2013[66]	Japan	23086669	201	94±12	<6	8
			628	96±19	6-7	
Sayin 2016[67]	Turkey	26978730	37	82.8±4.16	3-4	6
			48	87.19±8.5	≤2	
Shi 2008*[68]	Australia	18982012	330	90.54±19.62	<7	7
Song 2016[69]	China	27828862	8209	94.5±11.34	6-6.5	7
			2955	95.22±11.16	≤5.5	
Sun 2016[70]-female	China	27491292	1955	95.4±25.2	6-7	7
			1006	97.2±27	<6	
Sun 2016[70]-male	China	27491292	464	95.4±25.2	<6	
			1246	99±30.6	6-7	
Sun 2021[71]	China	34455367	1786	109.8±34.2	≤4	8
			3857	109.8±36	4-6	
Suzuki 2018[72]	Japan	28747590	118	101.7±21.6	<5.5	8
			278	102.2±19.3	5.5-6.4	
Taveras 2011[73]	US	20489690	147	77.1±17.6	≤5	7
Toyama 2013[74]	Japan	23081691	134	101.3±12.8	6-7	8
			92	105.2±23	5-6	
			30	110.4±43.8	<5	
Tuomilehto 2009[75]	Finland	19651919	47	109.8±16.2	≤6.5	7
Weil 2010*[76]	US	20725134	30	91.8±9.9	<7	6
Wu 2012[77]	China	22846501	1748	91.8±23.4	<6	8
Xu 2020[78]	China	32546151	601	97.2±20.7	≤5	7
			1710	98.46±21.96	6-7	
			841	98.64±21.96	5-6	
Yan 2018*[79]	China	29573578	1275	100.26±12.85	≤6	6
Yoo 2013[80]	US	23207742	32	90.3±9.7	≤6	8
Zheng 2015[81]	China	24981368	1214	93.6±7.2	<6	8
Fasting insulin (FINS), μIU/mL						

Bain 2017*[20]	US	28846879	15	5.27±2.34	<7	8
Brady 2018[21]	UK	29526681	476	10.9±6.6	6-6.5	6
			131	11±6.6	≤5.5	
Chaput 2013[25]	Canada	24099879	38	12.45±9.78	≤6	7
D'Aurea 2015[31]	Brazil	26083888	7	9.07±6.3	<5	8
Flint 2007[35]	US	17382111	14	25.7±12.6	≤6	8
Huang 2014#[37]	China	25056969	346	6.04±2.53	<6	7
Kanagasabai 2017[42]	Canada	28526258	2020	11.74±10.22	6	8
			389	12.74±12.1	4	
			934	12.81±21.52	5	
			141	14.14±12.65	≤3	
Rae 2018[82]	South Africa	30442325	90	6.68±4.02	<7	7
Rafalson 2010[64]	US	20620078	25	15.9±8.1	<6	8
Ryu 2015*[65]	Korea	25573816	3325	9.84±6.92	≤6	7
Taveras 2011[73]	US	20489690	147	10.8±7.1	≤5	7
Weil 2010*[76]	US	20725134	30	6.39±3.7	<7	6
Glycosylated hemoglobin (HbA1c), %						
Abe 2011[17]	Japan	21411100	687	5.4±0.5	6	7
			226	5.5±0.6	≤5	
Akiyama 2023[83]	Japan	36747481	749	7.7±1.8	<6	6
			949	7.4±1.6	6-7	
Brady 2018[21]	UK	29526681	131	5.9±0.5	≤5.5	6
			476	5.9±0.5	6-6.5	
Huang 2014[37]	China	25056969	346	5.73±0.43	<6	7
Im 2017[38]	Korea	28906359	3651	6±1	≤5	8
Jee 2017*[40]	Korea	27473575	347	7.4±1.86	<5	7
Kadono 2007[41]	Japan	17969468	1273	5.33±0.48	6-7	7
			554	5.36±0.52	5-6	
			133	5.41±0.58	≤5	
Kaneita 2008[43]	Japan	18517035	318	5.33±0.78	5-6	8
			596	5.4±0.86	6-7	
			70	5.41±0.97	<5	
Lin 2021[54]	China	34065152	544	5.1±0.4	≤4	6
			897	5.1±0.4	4-6	
Matsuo 2022[57]-female	Japan	35319079	1965	5.6±0.38	6-7	7
			1125	5.63±0.38	5-6	
			238	5.7±0.59	<5	
Matsuo 2022[57]-male	Japan	35319079	2857	5.73±0.62	6-7	
			1277	5.76±0.69	5-6	
			261	5.91±1.04	<5	
Niijima 2016[59]	Japan	27151211	716	5.9±0.8	<6	6
Okamura 2019[60]-female	Japan	30851175	1039	5.2±0.3	≤5	8
			2733	5.2±0.4	5-6	
			2128	5.2±0.4	6-7	

Okamura 2019[60]-male	Japan	30851175	889	5.2±0.5	≤5	
			2377	5.2±0.5	5-6	
			2068	5.2±0.5	6-7	
Ryu 2015*[65]	Korea	25573816	3325	7.56±10.38	≤6	7
Sun 2021[71]	China	34455367	1786	5.2±0.7	≤4	8
			3857	5.3±0.8	4-6	
Suzuki 2018[72]	Japan	28747590	118	5.6±0.7	<5.5	8
			278	5.6±0.8	5.5-6.4	
Tan 2019[84]	Singapore	30692966	629	7.58±1.57	5-6.9	7
			117	7.68±1.59	<5	
Yamamoto 2012[85]	Japan	22019276	3155	4.9±0.4	6	8
			1543	5±0.4	5	
			266	5±0.4	≤4	
Yan 2018*[79]	China	29573578	1275	5.1±1.07	≤6	6
Ye 2019#[86]	China	31469836	2854	5.9±0.45	6-7	7
Ye 2020#[87]	China	32593309	280	5.9±0.6	<6	7
Zheng 2015[81]	China	24981368	1214	5.7±0.5	<6	8
2h-glucose post-challenge (2hGlu), mg/dL						
Brady 2018[21]	UK	29526681	476	117±41.4	6-6.5	6
			131	118.8±45	≤5.5	
Huang 2014[37]	China	25056969	346	117±28.62	<6	7
Tuomilehto 2009[75]	Finland	19651919	47	160.2±25.2	≤6.5	7
**Normal sleep duration (7-9h)**						
Fasting blood glucose (FBG), mg/dL						
Abe 2011[17]	Japan	21411100	699	100.98±16.02	7	7
Adachi 2023[18]	Japan	36792215	441	101.7±26.3	7-8	8
			291	105.0±28.0	8-9	
Bain 2017*[20]	US	28846879	15	90±1.8	7-9	8
Brady 2018[21]	UK	29526681	1048	95.4±12.6	8-8.5	6
			900	97.2±18	7-7.5	
Brocato 2015[22]	US	26621514	292	130.8±47.3	7	7
Chang 2015[23]	China	26294202	172	98.3±20.84	7-7.9	8
Chaput 2013[25]	Canada	24099879	215	93.78±21.78	7-8	7
Chaudhry 2023 [24]	US	36839404	894	85.1±10.0	7-8	6
			1230	85.7±11.2	8-9	
Choi 2008[27]	Korea	18475274	1175	94.86±0.54	7	6
			981	96.66±0.72	8	
Choi 2017[29]-female	Korea	28192891	200	94.3±12.2	8-9	6
			273	95.6±15.7	7-8	
Choi 2017[29]-male	Korea	28192891	119	101.7±22.4	8-9	
			185	102.5±23.5	7-8	
Chou 2020[30]	China	32941519	2087	90±18	7-9	7

Feng 2016*[33]	China	27966597	349	88.74±13.5	7-9	6
Han 2016[88]	China	26969344	6864<br>4628	102.06±17.64<br>102.42±19.44	8-9<br>7-8	6
Hayes 2011[36]	US	21286230	96	97±24.5	7-8	7
Kadono 2007[41]	Japan	17969468	783	100.3±14.3	7-8	7
Kanagasabai 2017[42]	Canada	28526258	2393<br>406<br>2331	106.2±28.8<br>108±27<br>109.8±37.8	7<br>9<br>8	8
Kaneita 2008[43]	Japan	18517035	455	108.8±43.9	7-8	8
Katano 2011[44]	Japan	24843515	1415	96.3±23.7	7-8	7
Kim 2015[46]	Korea	26359509	2869<br>9416	97.8±15<br>98.3±15.8	8<br>7	7
Li 2015[47]	China	26116460	936<br>1466	82.8±12.24<br>85.14±17.64	8-9<br>7-8	6
Li 2018(A)[48]	China	29415800	35<br>50	91.8±5.4<br>93.6±7.2	7-8<br>8-9	7
Li 2018(B)[49]	China	30408698	2407<br>2381	90.9±40.86<br>91.26±32.04	8-9<br>7-8	7
Lin 2016(A)[50]	China	26922430	841	104.4±29.5	7-9	7
Lin 2018[53]	China	28238100	3031<br>5443	106.2±27<br>108±27	7-8<br>8-9	7
Liu 2016[55]	China	27327959	2549<br>3743	104.4±23.4<br>104.4±23.4	7-8<br>8-9	7
Marshall 2010[56]	Australia	10.1111/j.1479-8425.2010.00431.x	352	92.16±34.92	7	8
Matsuo 2022[57]-female<br>Matsuo 2022[57]-male	Japan	35319079	1023<br>1724	90±10<br>100±18	7-8	7
Min 2016[58]	Korea	27956898	2597<br>2082	91.1±11.2<br>91.6±12.6	7<br>8	8
Park 2018[61]	Korea	30092896	39261	94.4±13.5	7-9	7
Rae 2018[82]	South Africa	30442325	235	80.82±7.74	7-9	7
Ryu 2015*[65]	Korea	25573816	2212<br>2815	94.68±25.4<br>95.22±19.1	8<br>7	7
Satoh 2013[66]	Japan	23086669	638	97±20	7-8	8
Sayin 2016[67]	Turkey	26978730	17	85.31±6.9	8-9	6
Shi 2008*[68]	Australia	18982012	1954	90.18±23.94	7-9	7
Song 2016[69]	China	27828862	27052<br>2272	93.6±11.16<br>94.14±11.16	7.5-8<br>7	7
Sun 2016[70]-female<br>Sun 2016[70]-male	China	27491292	3119<br>4133<br>1908	95.4±21.6<br>95.4±28.8<br>97.2±28.8	7-8<br>8-9<br>7-8	7

			2636	99±36	8-9	
Suzuki 2018[72]	Japan	28747590	223	104.8±24	7.5-8.4	8
Tuomilehto 2009[75]	Finland	19651919	222	109.8±12.6	7-8.5	7
Weil 2010*[76]	US	20725134	50	91.8±1.8	7-9	6
Xu 2020[78]	China	32546151	1757	100.26±23.4	7-8	7
Zeng 2021[89]	China	33316114	336	111.6±27.54	8-8.5	6
Fasting insulin (FINS), µIU/mL						
Bain 2017*[20]	US	28846879	15	5.21±4.05	7-9	8
Brady 2018[21]	UK	29526681	900<br>1048	9.5±6.2<br>10.8±7.5	7-7.5<br>8-8.5	6
Chaput 2013[25]	Canada	24099879	215	8.73±7.01	7-8	7
Kanagasabai 2017[42]	Canada	28526258	406<br>2393<br>2331	10.93±8.66<br>10.94±10.85<br>11.11±9.53	9<br>7<br>8	8
Ryu 2015*[65]	Korea	25573816	2212<br>2815	9.95±6.58<br>10.27±8.49	8<br>7	7
Weil 2010*[76]	US	20725134	50	5.46±3.76	7-9	6
Glycosylated hemoglobin (HbA1c), %						
Abe 2011[17]	Japan	21411100	699	5.4±0.5	7	7
Akiyama 2023[83]	Japan	36747481	750	7.5±1.7	7-8	6
Brady 2018[21]	UK	29526681	1048<br>900	5.9±0.5<br>6±0.6	8-8.5<br>7-7.5	6
Kadono 2007[41]	Japan	17969468	783	5.36±0.59	7-8	7
Kaneita 2008[43]	Japan	18517035	455	5.47±1.07	7-8	8
Matsuo 2022[57]-female<br>Matsuo 2022[57]-male	Japan	35319079	1023<br>1724	5.58±0.38<br>5.75±0.61	7-8	7
Ryu 2015*[65]	Korea	25573816	2815<br>2212	7.2±7.43<br>7.42±8	7<br>8	7
Suzuki 2018[72]	Japan	28747590	223	5.8±0.9	7.5-8.4	8
Tan 2019[84]	Singapore	30692966	429	7.62±1.49	7-8	7
Ye 2019#[86]	China	31469836	16492	5.9±0.44	7-8	7
Ye 2020#[87]	China	32593309	2114	5.9±0.6	8-9	7
Zeng 2021[81]	China	33316114	336	6.23±0.87	8-8.5	6
2h-glucose post-challenge (2hGlu), mg/dL						
Brady 2018[21]	UK	29526681	900<br>1048	118.8±50.4<br>117±41.4	7-7.5<br>8-8.5	6
Tuomilehto 2009[75]	Finland	19651919	222	156.6±25.2	7-8.5	7
**Long sleep duration (>9h)**						
Fasting blood glucose (FBG), mg/dL						
Adachi 2023[18]	Japan	36792215	209	112.9±39.5	≥9	8

Brady 2018[21]	UK	29526681	293	95.4±16.2	≥9	6
Chaput 2013[25]	Canada	24099879	40	88.74±8.82	≥9	7
Chaudhry 2023 [26]	US	36839404	1292	85.8±10.2	≥9	6
Choi 2008[27]	Korea	18475274	284	96.12±0.9	≥9	6
Choi 2011[28]	Korea	22001675	18	101.39±21.12	≥10	6
Choi 2017[29]-female<br>Choi 2017[29]-male	Korea	28192891	86<br>66	97.3±16.4<br>105.5±28.5	≥9	6
Chou 2020[30]	China	32941519	19	92.6±30.5	≥9	7
Feng 2016*[33]	China	27966597	96	88.74±12.42	≥9	6
Han 2016[88]	China	26969344	830<br>2775	102.24±19.44<br>102.24±17.82	≥10<br>9-10	6
Im 2017[38]	Korea	28906359	2220	98±29	≥9	8
Jee 2017*[40]	Korea	27473575	154	152.1±55.8	>9	7
Kanagasabai 2017[42]	Canada	28526258	213	113.4±43.2	≥10	8
Kim 2015[46]	Korea	26359509	535	97.8±19.1	≥9	7
Li 2015[47]	China	26116460	206	81.9±9	≥9	6
Li 2018(A)[48]	China	29415800	13<br>26	88.2±5.4<br>91.8±7.2	>10<br>9-10	7
Li 2018(B)[49]	China	30408698	434	93.96±34.38	≥9	7
Lin 2016(A)[50]	China	26922430	155	106±32.1	≥9	7
Lin 2018[53]	China	28238100	2414<br>3967	106.2±27<br>106.2±27	≥10<br>9-10	7
Liu 2016[55]	China	27327959	1964	106.2±28.8	≥9	7
Min 2016[58]	Korea	27956898	670	90.8±11.2	≥9	8
Park 2018[61]	Korea	30092896	1671	91.8±123	≥9	7
Prakaschandra 2023[63]	South Africa	37092708	56	5.46±2.87	>10	7
Rae 2018[82]	South Africa	30442325	77	82.44±10.44	≥9	7
Ryu 2015*[65]	Korea	25573816	725	95.04±24.3	≥9	7
Sayin 2016[67]	Turkey	26978730	19<br>15<br>10	88.54±7.6<br>89.24±7.9<br>90.11±12.3	9-10<br>≥11<br>≥10	6
Shi 2008*[68]	Australia	18982012	544	89.64±21.06	≥9	7
Sun 2016[70]-female<br>Sun 2016[70]-male	China	27491292	2435<br>1603	97.2±34.2<br>97.2±30.6	≥9	7
Sun 2021[71]	China	34455367	81	104.4±30.6	>10	8
Tuomilehto 2009[75]	Finland	19651919	115<br>131	109.8±12.6<br>111.6±14.4	9-9.5<br>≥10	7
Zheng 2015[81]	China	24981368	1124	93.6±7.2	>9	8
Zhou 2010#[90]	China	20513296	28<br>84	79.2±11.88<br>84.6±8.1	≥10<br>9-10	6

Fasting insulin (FINS), μIU/mL

Brady 2018[21]	UK	29526681	293	14.3±9.6	≥9	6
Chaput 2013[25]	Canada	24099879	40	11.67±9.89	≥9	7
Kanagasabai 2017[42]	Canada	28526258	213	14.39±23.3	≥10	8
Rae 2018[82]	South Africa	30442325	77	13.81±9.87	>9	7
Ryu 2015*[65]	Korea	25573816	725	10.18±5.39	≥9	7
Zhou 2010#[90]	China	24981368	28	16.65±7.12	≥10	6
			84	16.75±8.11	9-10	
Glycosylated hemoglobin (HbA1c), %						
Brady 2018[21]	UK	29526681	293	5.9±0.5	≥9	6
Im 2017[38]	Korea	28906359	2220	6.1±1.1	≥9	8
Jee 2017*[40]	Korea	27473575	154	7.5±1.24	>9	7
Ryu 2015*[65]	Korea	25573816	725	7.83±7.54	≥9	7
Sun 2021[71]	China	34455367	81	5.4±0.6	>10	8
Zheng 2015[81]	China	24981368	1124	5.7±0.4	>9	8
2h-glucose post-challenge (2hGlu), mg/dL						
Brady 2018[21]	UK	29526681	293	122.4±45	≥9	6
Tuomilehto 2009[75]	Finland	19651919	131	160.2±27	≥10	7
			115	163.8±28.8	9-9.5	

Data are expressed as mean±SD unless specifically labeled. *: Data are expressed as means±SE; #: Data are expressed as median (interquartile). NO: Number; NOS: Newcastle-Ottawa Scale.

Supplemental table 5. Assessment of heterogeneity and pleiotropy in the causal relationship between sleep disturbances and glycemic traits in bidirectional Mendelian Randomization (MR) analysis.

Method	No. SNPs	Heterogeneity			Pleiotropy		
		Cochrane's Q	df	*p*-value	Intercept	se	*p*-value
**Before excluding outliers**							
2hGlu→EDS							
IVW	13	52.33	12	5.43×10^{-7}	NA	NA	NA
MR Egger	13	47.59	11	1.69×10^{-6}	0.00201	0.00192	0.318
FBG→insomnia							
IVW	68	131.28	67	4.55×10^{-6}	NA	NA	NA
MR Egger	68	131.18	66	3.26×10^{-6}	0.000122	0.000534	0.820
2hGlu→insomnia							
IVW	13	26.11	12	0.0103	NA	NA	NA
MR Egger	13	23.41	11	0.0155	-0.00205	0.00182	0.284
FINS→sleep duration							
IVW	35	32.17	34	0.558	NA	NA	NA
MR Egger	35	30.96	33	0.569	-0.00153	0.00139	0.280
FINS→short sleep duration							
IVW	35	38.01	34	0.292	NA	NA	NA
MR Egger	35	37.03	33	0.288	0.000583	0.000624	0.357
short sleep duration→HbA1c							
IVW	24	31.70	23	0.106	NA	NA	NA
MR Egger	24	31.43	22	0.0877	-0.000791	0.00181	0.666
**After excluding outliers**							
2hGlu→EDS							
IVW	10	7.79	9	0.555	NA	NA	NA
MR Egger	10	7.79	8	0.454	7.11×10^{-6}	0.00113	0.995
FBG→insomnia							
IVW	59	53.84	58	0.631	NA	NA	NA
MR Egger	59	53.81	57	0.595	7.05×10^{-5}	0.000405	0.863
2hGlu→insomnia							
IVW	11	15.36	10	0.120	NA	NA	NA
MR Egger	11	14.34	9	0.111	-0.00140	0.00175	0.444

Every causal analysis possesses a corresponding assessment of heterogeneity and pleiotropy. $p > 0.05$ is considered as the threshold for the existence of no heterogeneity/pleiotropy.

Abbreviation: NO: Number; SNP: Single nucleotide polymorphism; EDS: excessive daytime sleepiness; IVW: Inverse variance weighting; FBG: Fasting blood glucose; FINS: Fasting insulin; HbA1c: Glycosylated hemoglobin; 2hGlu: 2h-glucose post-challenge; NA: Not available.

Supplemental Table 6. Results of multivariable Mendelian randomization.

Exposure	Outcome	nSNPs	Beta[95%CI]	OR [95%CI]	*p value*
2hGlu	EDS	12	0.020[0.007,0.034]	1.020[1.007,1.035]	**0.004**
CHD	EDS	35	0.002[-0.004,0.009]	1.002[0.996,1.009]	0.452
2hGlu	EDS	7	0.026[0.010,0.042]	1.026[1.010,1.043]	**0.001**
T2D	EDS	113	-0.003[-0.009,0.002]	0.997[0.991,1.002]	0.215
2hGlu	insomnia	12	0.022[0.001,0.042]	1.022[1.001,1.043]	**0.036**
CHD	insomnia	35	-0.002[-0.012,0.007]	0.998[0.989,1.007]	0.663
2hGlu	insomnia	7	-0.003[-0.026,0.020]	0.997[0.974,1.020]	0.787
T2D	insomnia	113	0.006[-0.001,0.014]	1.006[0.999,1.014]	0.107
FINS	short sleep duration	32	0.029[0.003,0.054]	1.029[1.003,1.056]	**0.027**
CHD	short sleep duration	33	0.005[0.001,0.010]	1.005[1.001,1.010]	**0.026**
FINS	short sleep duration	22	0.030[-0.005,0.065]	1.031[0.995,1.067]	0.089
T2D	short sleep duration	106	0.004[-0.001,0.008]	1.004[0.999,1.008]	0.088
short sleep duration	HbA1c	22	0.130[-0.046,0.306]	-	0.147
CHD	HbA1c	37	-0.003[-0.013,0.008]	-	0.630
short sleep duration	HbA1c	11	0.075[-0.188,0.339]	-	0.575
T2D	HbA1c	113	0.061[0.051,0.071]	-	**2.857×10^{-33}**

Abbreviations: SNP: Single nucleotide polymorphism; OR: Odds ratios; EDS: excessive daytime sleepiness; FINS: Fasting insulin; 2hGlu: 2h-glucose post-challenge; CHD: Coronary heart disease; T2D: Type II diabetes.

1.2 Supplemental Figures

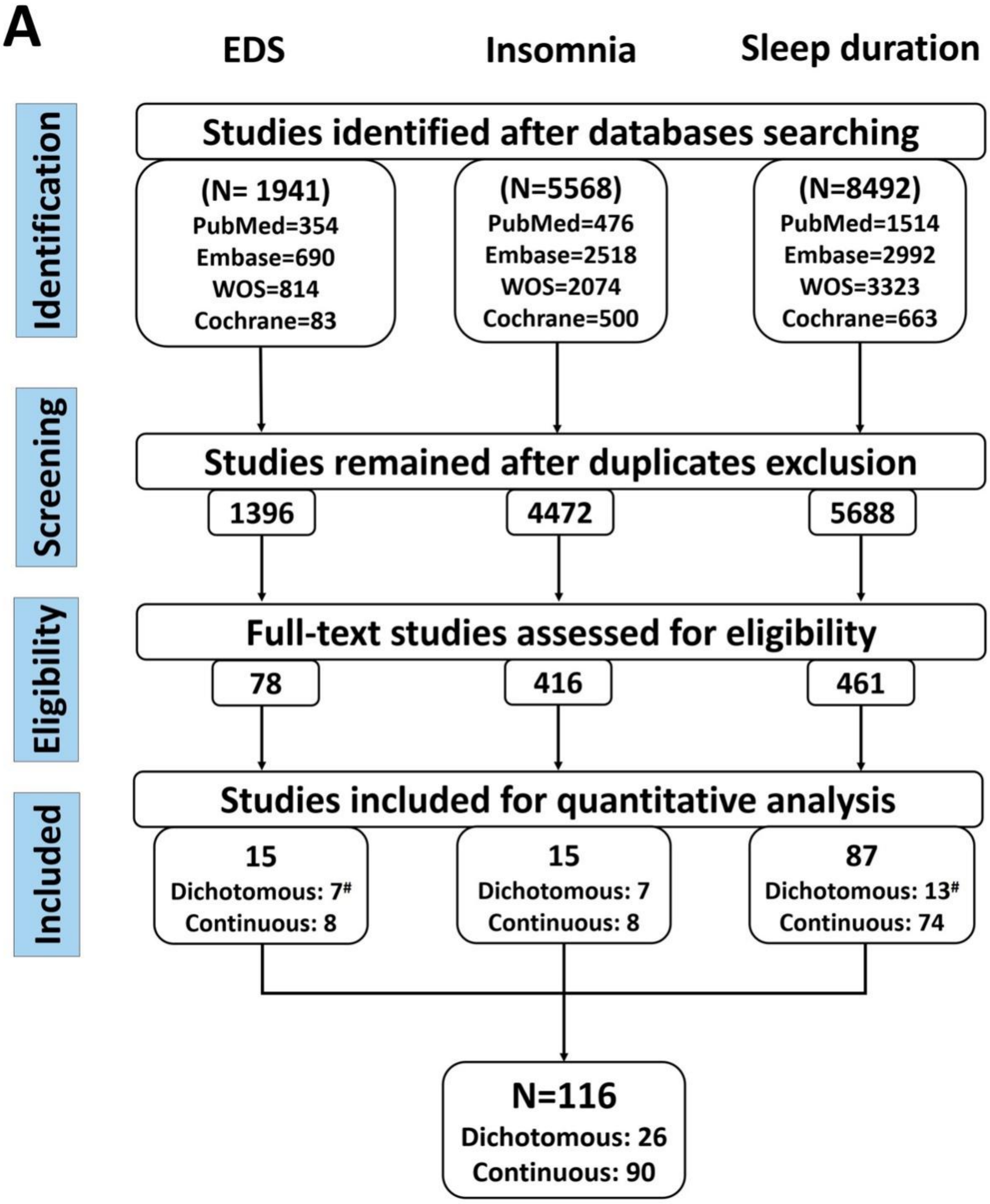

B

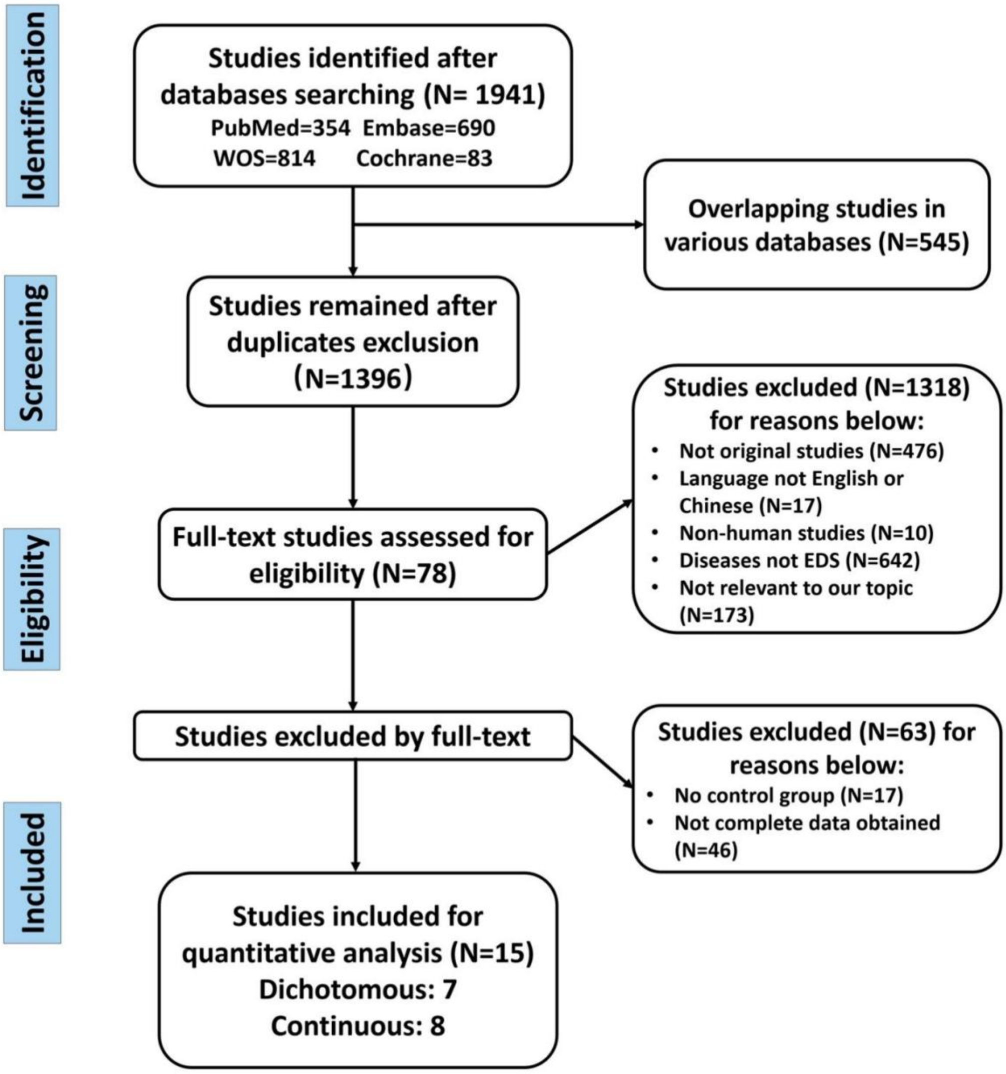
Identification
Screening
Eligibility
Included
Studies identified after databases searching (N= 1941)
PubMed=354 Embase=690
WOS=814 Cochrane=83
Overlapping studies in various databases (N=545)
Studies remained after duplicates exclusion (N=1396)
Studies excluded (N=1318) for reasons below:
Not original studies (N=476)
Language not English or Chinese (N=17)
Non-human studies (N=10)
Diseases not EDS (N=642)
Not relevant to our topic (N=173)
Full-text studies assessed for eligibility (N=78)
Studies excluded by full-text
Studies excluded (N=63) for reasons below:
No control group (N=17)
Not complete data obtained (N=46)
Studies included for quantitative analysis (N=15)
Dichotomous: 7
Continuous: 8

C

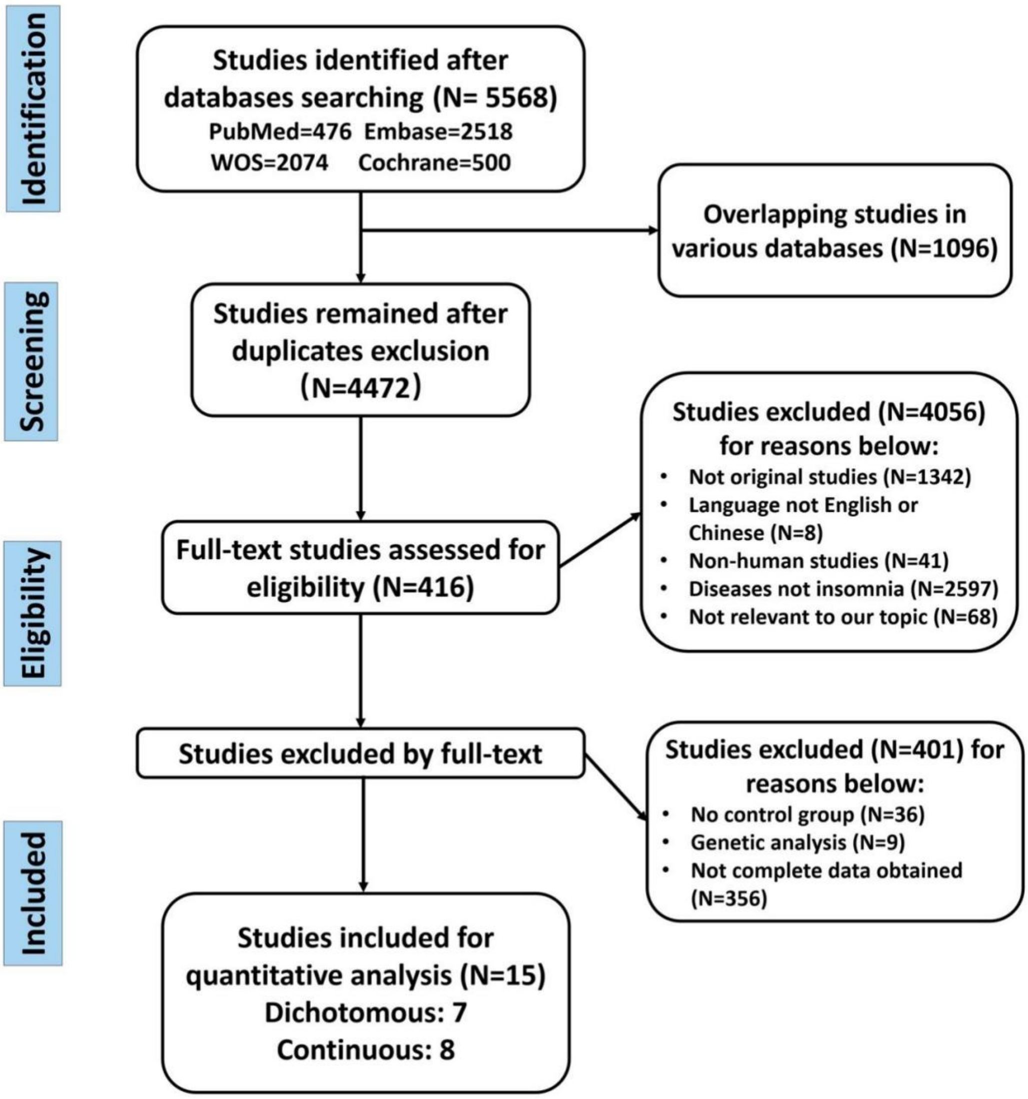
Identification
Screening
Eligibility
Included
Studies identified after databases searching (N= 5568)
PubMed=476 Embase=2518
WOS=2074 Cochrane=500
Overlapping studies in various databases (N=1096)
Studies remained after duplicates exclusion (N=4472)
Studies excluded (N=4056) for reasons below:
Not original studies (N=1342)
Language not English or Chinese (N=8)
Non-human studies (N=41)
Diseases not insomnia (N=2597)
Not relevant to our topic (N=68)
Full-text studies assessed for eligibility (N=416)
Studies excluded by full-text
Studies excluded (N=401) for reasons below:
No control group (N=36)
Genetic analysis (N=9)
Not complete data obtained (N=356)
Studies included for quantitative analysis (N=15)
Dichotomous: 7
Continuous: 8

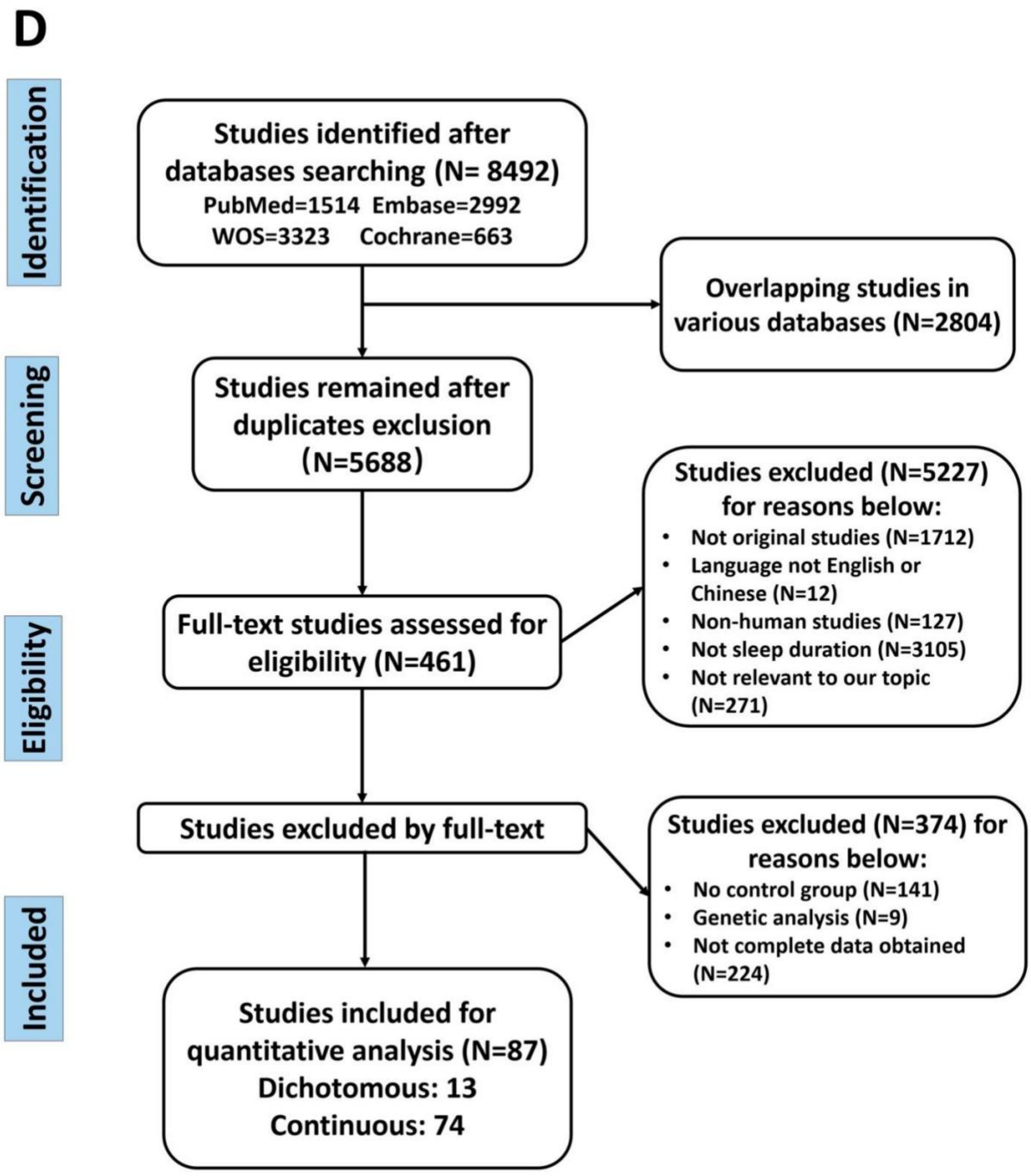

Supplemental Figure 1. Flow diagram of study selection process based on inclusion and exclusion criteria. (A) Total; (B) EDS; (C) insomnia; (D) sleep duration. EDS: excessive daytime sleepiness. #There is one overlapping publication.

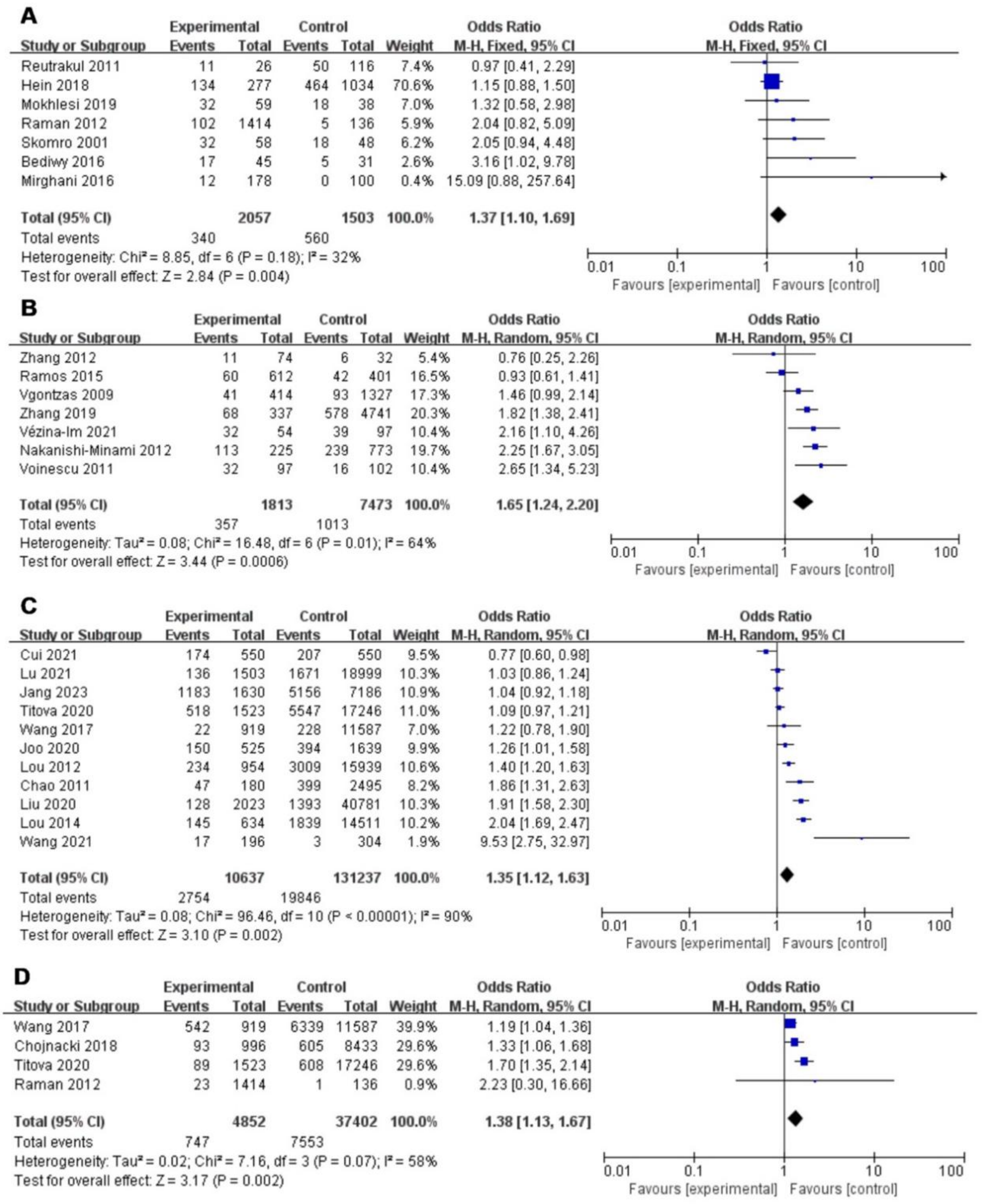

Supplemental Figure 2. Forest plots of ORs of sleep disturbances associated with abnormal glucose metabolism in the meta-analysis. (A) excessive daytime sleepiness; (B) insomnia; (C) short sleep duration; (D) long sleep duration.

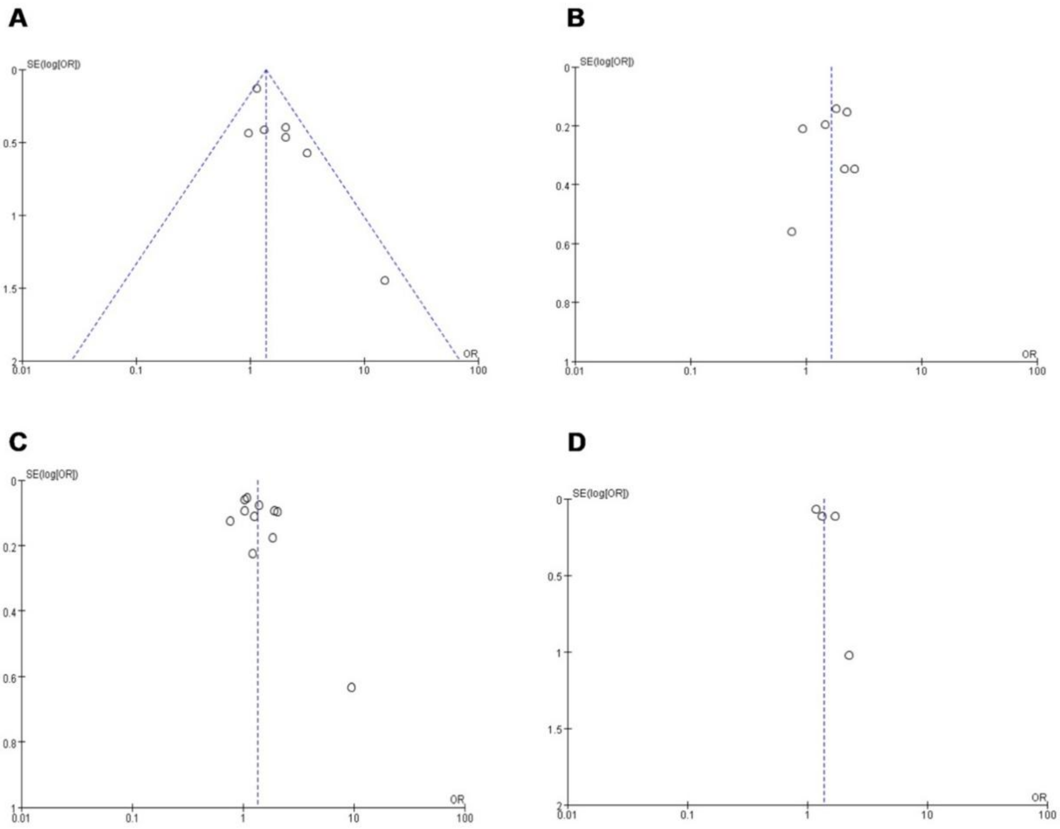

Supplemental Figure 3. Funnel plots of ORs of sleep disturbances associated with abnormal glucose metabolism in the meta-analysis. (A) excessive daytime sleepiness; (B) insomnia; (C) short sleep duration; (D) long sleep duration.

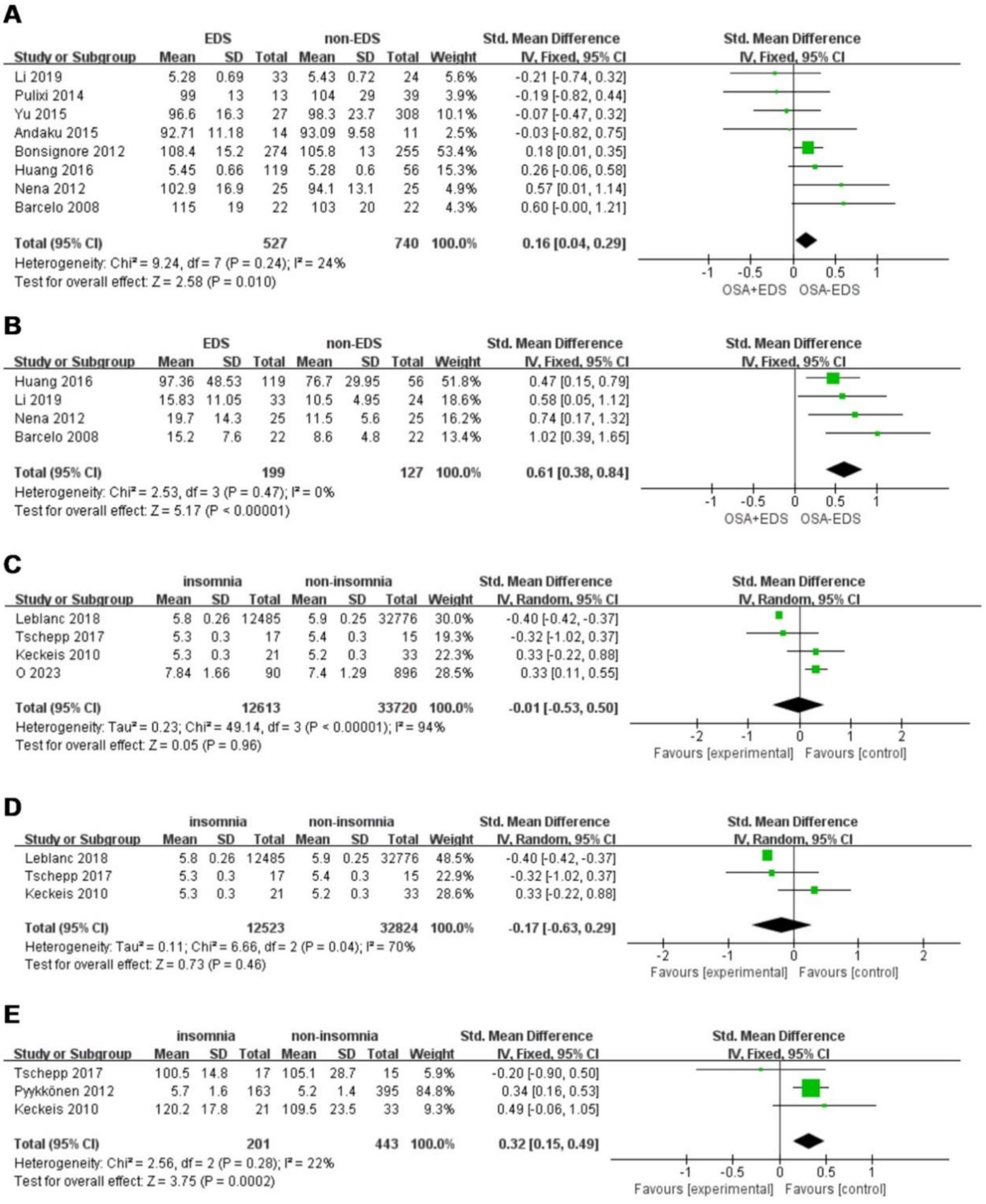

Supplemental Figure 4. Forest plots of glycemic traits levels compared in patients with and without EDS (A+B) as well as patients with and without insomnia (C+D+E) in the meta-analysis. (A) FBG; (B) FINS; (C) FBG; (D)HbA1c; (E) 2hGlu. Abbreviation: EDS: excessive daytime sleepiness; FBG: Fasting blood glucose; HbA1c: Glycosylated hemoglobin; FINS: Fasting insulin; 2hGlu: 2h-glucose post-challenge.

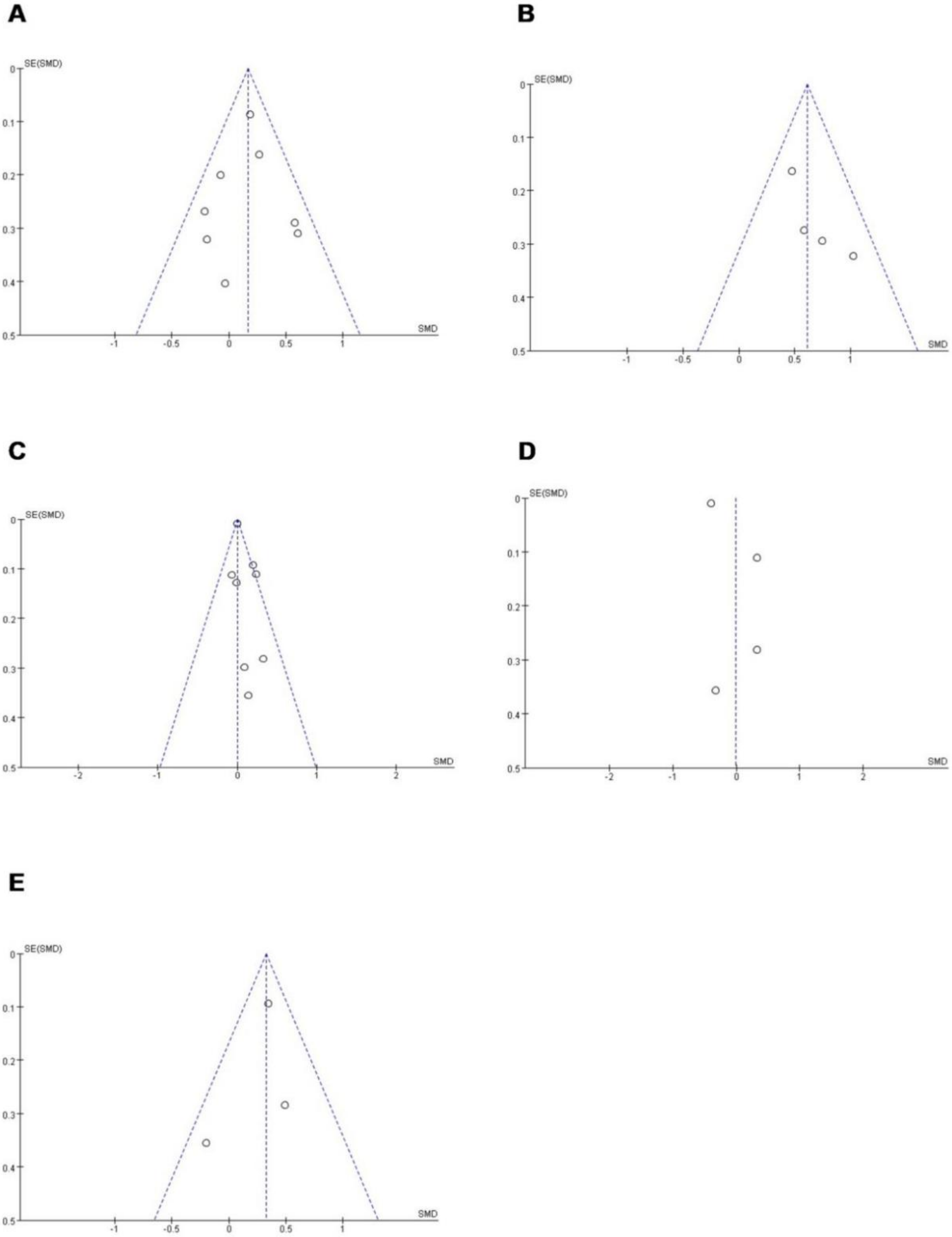

Supplemental Figure 5. Funnel plots of glycemic traits levels compared in patients with and without EDS (A+B) as well as patients with and without insomnia (C+D+E) in the meta-analysis. (A) FBG; (B) FINS; (C) FBG; (D) HbA1c; (E) 2hGlu.Abbreviation: EDS: excessive daytime sleepiness; FBG: Fasting blood glucose; HbA1c: Glycosylated hemoglobin; FINS: Fasting insulin; 2hGlu: 2h-glucose post-challenge.

A

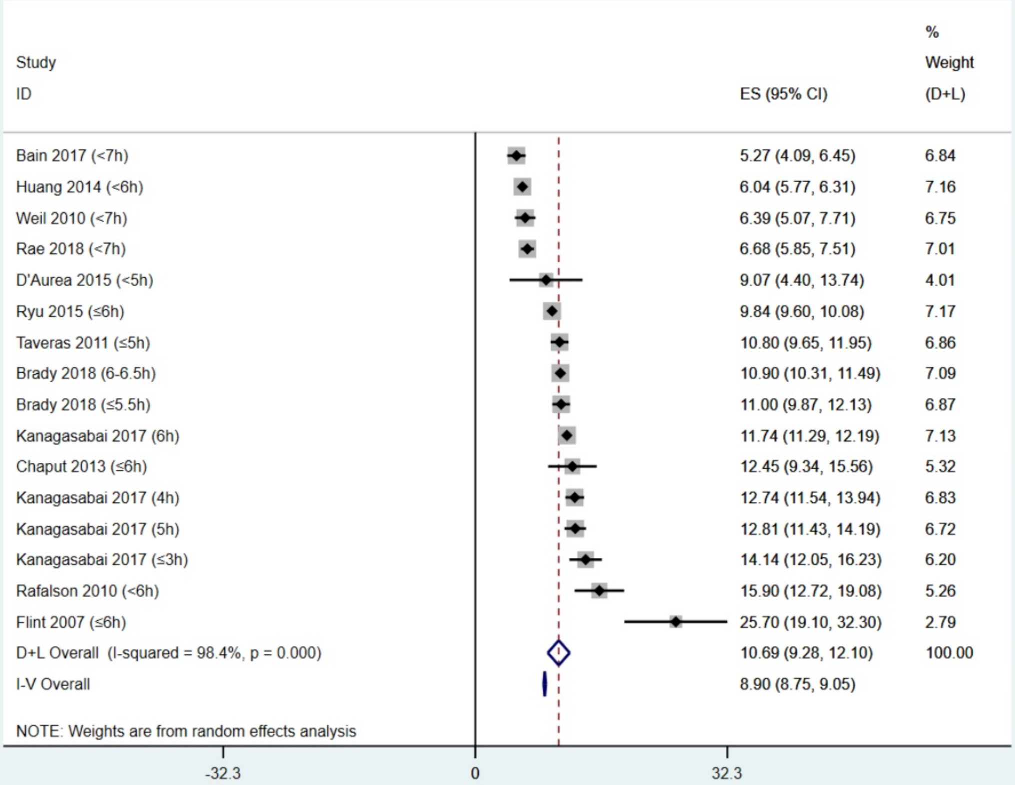
Study
ID
ES (95% CI)
%
Weight
(D+L)
Bain 2017 (<7h) 5.27 (4.09, 6.45) 6.84
Huang 2014 (<6h) 6.04 (5.77, 6.31) 7.16
Weil 2010 (<7h) 6.39 (5.07, 7.71) 6.75
Rae 2018 (<7h) 6.68 (5.85, 7.51) 7.01
D'Aurea 2015 (<5h) 9.07 (4.40, 13.74) 4.01
Ryu 2015 (≤6h) 9.84 (9.60, 10.08) 7.17
Taveras 2011 (≤5h) 10.80 (9.65, 11.95) 6.86
Brady 2018 (6-6.5h) 10.90 (10.31, 11.49) 7.09
Brady 2018 (≤5.5h) 11.00 (9.87, 12.13) 6.87
Kanagasabai 2017 (6h) 11.74 (11.29, 12.19) 7.13
Chaput 2013 (≤6h) 12.45 (9.34, 15.56) 5.32
Kanagasabai 2017 (4h) 12.74 (11.54, 13.94) 6.83
Kanagasabai 2017 (5h) 12.81 (11.43, 14.19) 6.72
Kanagasabai 2017 (≤3h) 14.14 (12.05, 16.23) 6.20
Rafalson 2010 (<6h) 15.90 (12.72, 19.08) 5.26
Flint 2007 (≤6h) 25.70 (19.10, 32.30) 2.79
D+L Overall (I-squared = 98.4%, p = 0.000) 10.69 (9.28, 12.10) 100.00
I-V Overall 8.90 (8.75, 9.05)
NOTE: Weights are from random effects analysis
-32.3
0
32.3

B

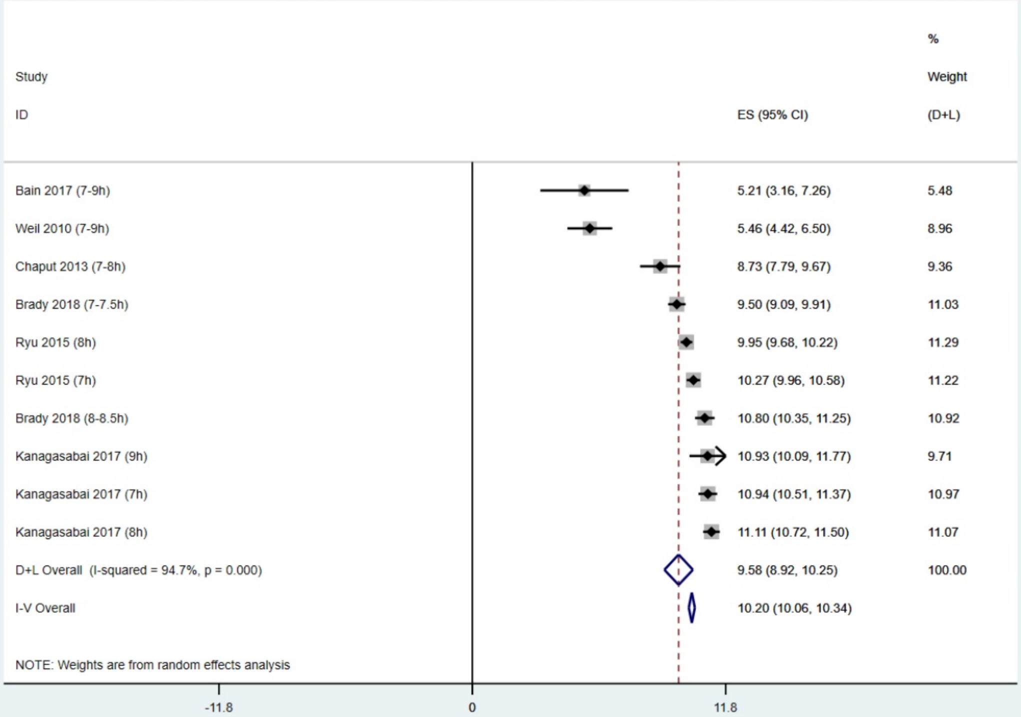
Study
ID
ES (95% CI)
%
Weight
(D+L)
Bain 2017 (7-9h) 5.21 (3.16, 7.26) 5.48
Weil 2010 (7-9h) 5.46 (4.42, 6.50) 8.96
Chaput 2013 (7-8h) 8.73 (7.79, 9.67) 9.36
Brady 2018 (7-7.5h) 9.50 (9.09, 9.91) 11.03
Ryu 2015 (8h) 9.95 (9.68, 10.22) 11.29
Ryu 2015 (7h) 10.27 (9.96, 10.58) 11.22
Brady 2018 (8-8.5h) 10.80 (10.35, 11.25) 10.92
Kanagasabai 2017 (9h) 10.93 (10.09, 11.77) 9.71
Kanagasabai 2017 (7h) 10.94 (10.51, 11.37) 10.97
Kanagasabai 2017 (8h) 11.11 (10.72, 11.50) 11.07
D+L Overall (I-squared = 94.7%, p = 0.000) 9.58 (8.92, 10.25) 100.00
I-V Overall 10.20 (10.06, 10.34)
NOTE: Weights are from random effects analysis
-11.8
0
11.8

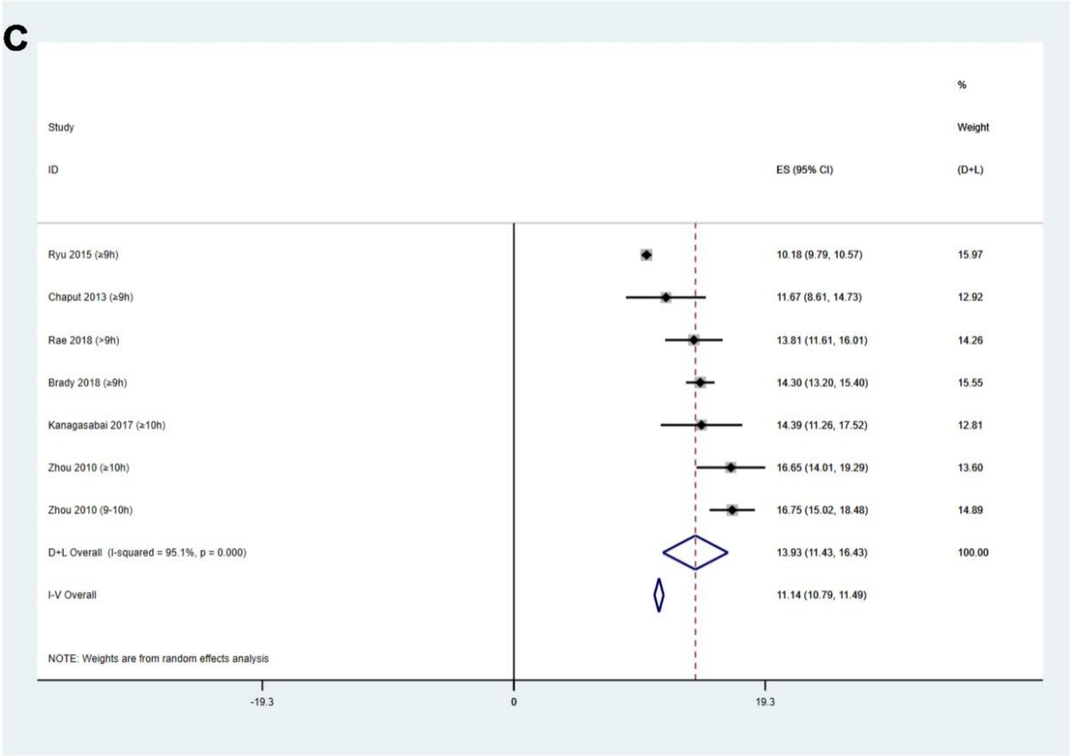

Supplemental Figure 6. Forest plots of fasting insulin (FINS) of different sleep duration in the single-arm meta-analysis. (A) short, (B) normal, (C) long sleep duration.

A

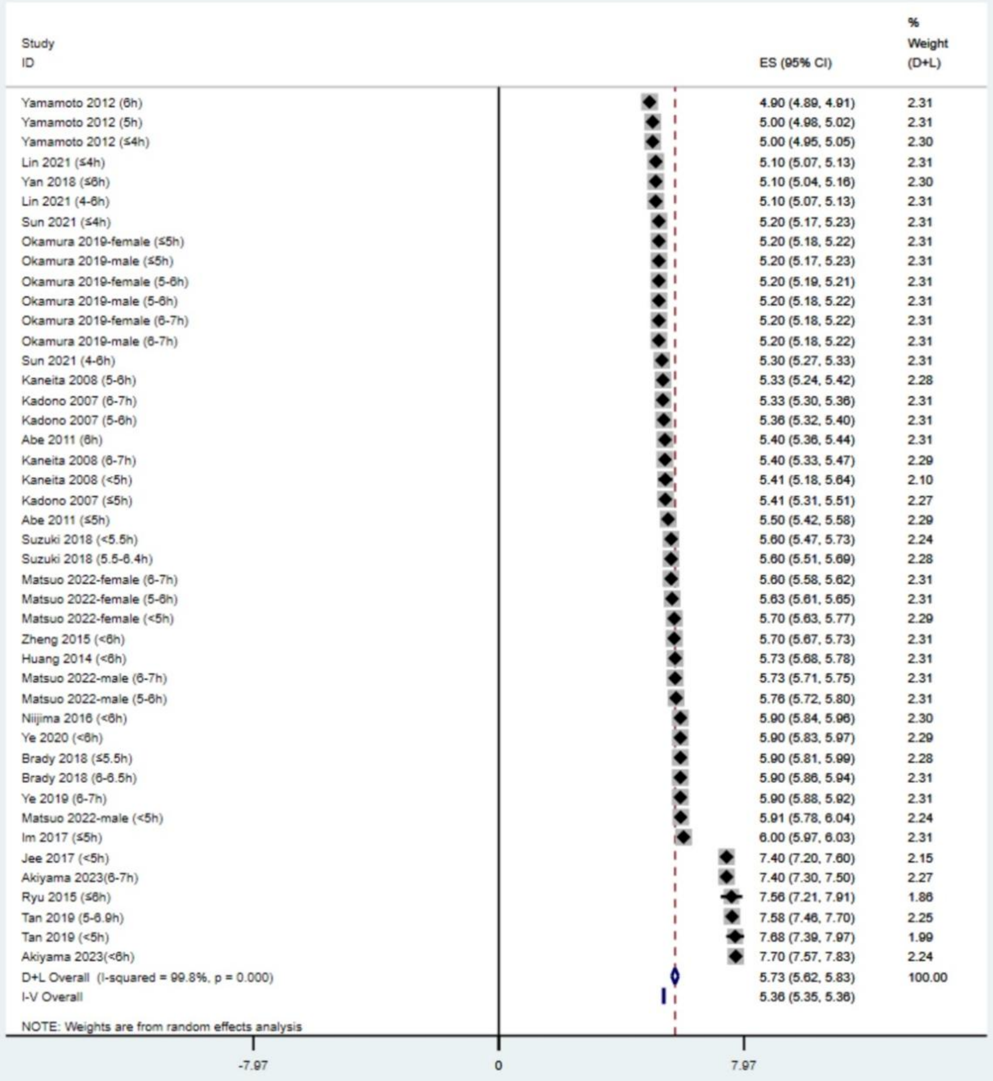

Study
ID
ES (95% CI)
% Weight (D+L)
Yamamoto 2012 (6h) 4.90 (4.89, 4.91) 2.31
Yamamoto 2012 (5h) 5.00 (4.98, 5.02) 2.31
Yamamoto 2012 (≤4h) 5.00 (4.95, 5.05) 2.30
Lin 2021 (≤4h) 5.10 (5.07, 5.13) 2.31
Yan 2018 (≤6h) 5.10 (5.04, 5.16) 2.30
Lin 2021 (4-6h) 5.10 (5.07, 5.13) 2.31
Sun 2021 (≤4h) 5.20 (5.17, 5.23) 2.31
Okamura 2019-female (≤5h) 5.20 (5.18, 5.22) 2.31
Okamura 2019-male (≤5h) 5.20 (5.17, 5.23) 2.31
Okamura 2019-female (5-6h) 5.20 (5.19, 5.21) 2.31
Okamura 2019-male (5-6h) 5.20 (5.18, 5.22) 2.31
Okamura 2019-female (6-7h) 5.20 (5.18, 5.22) 2.31
Okamura 2019-male (6-7h) 5.20 (5.18, 5.22) 2.31
Sun 2021 (4-6h) 5.30 (5.27, 5.33) 2.31
Kaneita 2008 (5-6h) 5.33 (5.24, 5.42) 2.28
Kadono 2007 (6-7h) 5.33 (5.30, 5.36) 2.31
Kadono 2007 (5-6h) 5.36 (5.32, 5.40) 2.31
Abe 2011 (6h) 5.40 (5.36, 5.44) 2.31
Kaneita 2008 (6-7h) 5.40 (5.33, 5.47) 2.29
Kaneita 2008 (<5h) 5.41 (5.18, 5.64) 2.10
Kadono 2007 (≤5h) 5.41 (5.31, 5.51) 2.27
Abe 2011 (≤5h) 5.50 (5.42, 5.58) 2.29
Suzuki 2018 (<5.5h) 5.60 (5.47, 5.73) 2.24
Suzuki 2018 (5.5-6.4h) 5.60 (5.51, 5.69) 2.28
Matsuo 2022-female (6-7h) 5.60 (5.58, 5.62) 2.31
Matsuo 2022-female (5-6h) 5.63 (5.61, 5.65) 2.31
Matsuo 2022-female (<5h) 5.70 (5.63, 5.77) 2.29
Zheng 2015 (<6h) 5.70 (5.67, 5.73) 2.31
Huang 2014 (<6h) 5.73 (5.68, 5.78) 2.31
Matsuo 2022-male (6-7h) 5.73 (5.71, 5.75) 2.31
Matsuo 2022-male (5-6h) 5.76 (5.72, 5.80) 2.31
Niijima 2016 (<6h) 5.90 (5.84, 5.96) 2.30
Ye 2020 (<6h) 5.90 (5.83, 5.97) 2.29
Brady 2018 (≤5.5h) 5.90 (5.81, 5.99) 2.28
Brady 2018 (6-6.5h) 5.90 (5.86, 5.94) 2.31
Ye 2019 (6-7h) 5.90 (5.88, 5.92) 2.31
Matsuo 2022-male (<5h) 5.91 (5.78, 6.04) 2.24
Im 2017 (≤5h) 6.00 (5.97, 6.03) 2.31
Jee 2017 (<5h) 7.40 (7.20, 7.60) 2.15
Akiyama 2023(6-7h) 7.40 (7.30, 7.50) 2.27
Ryu 2015 (≤6h) 7.56 (7.21, 7.91) 1.86
Tan 2019 (5-6.9h) 7.58 (7.46, 7.70) 2.25
Tan 2019 (<5h) 7.68 (7.39, 7.97) 1.99
Akiyama 2023(<6h) 7.70 (7.57, 7.83) 2.24
D+L Overall (I-squared = 99.8%, p = 0.000) 5.73 (5.62, 5.83) 100.00
I-V Overall 5.36 (5.35, 5.36)
NOTE: Weights are from random effects analysis
-7.97
0
7.97

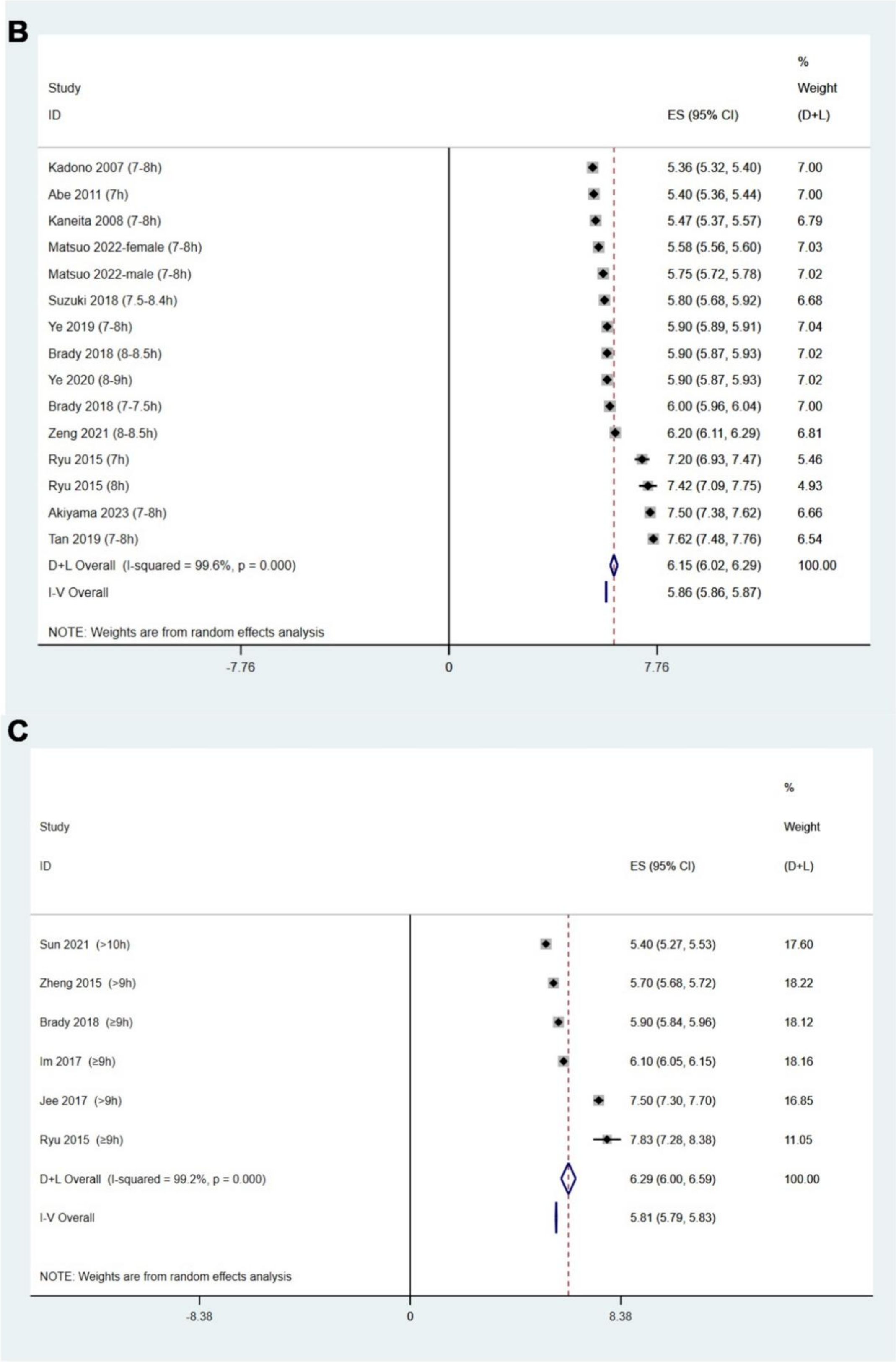

Supplemental Figure 7. Forest plots of glycosylated hemoglobin (HbA1c) of different sleep duration in the single-arm meta-analysis. (A) short, (B) normal, (C) long sleep duration.

A

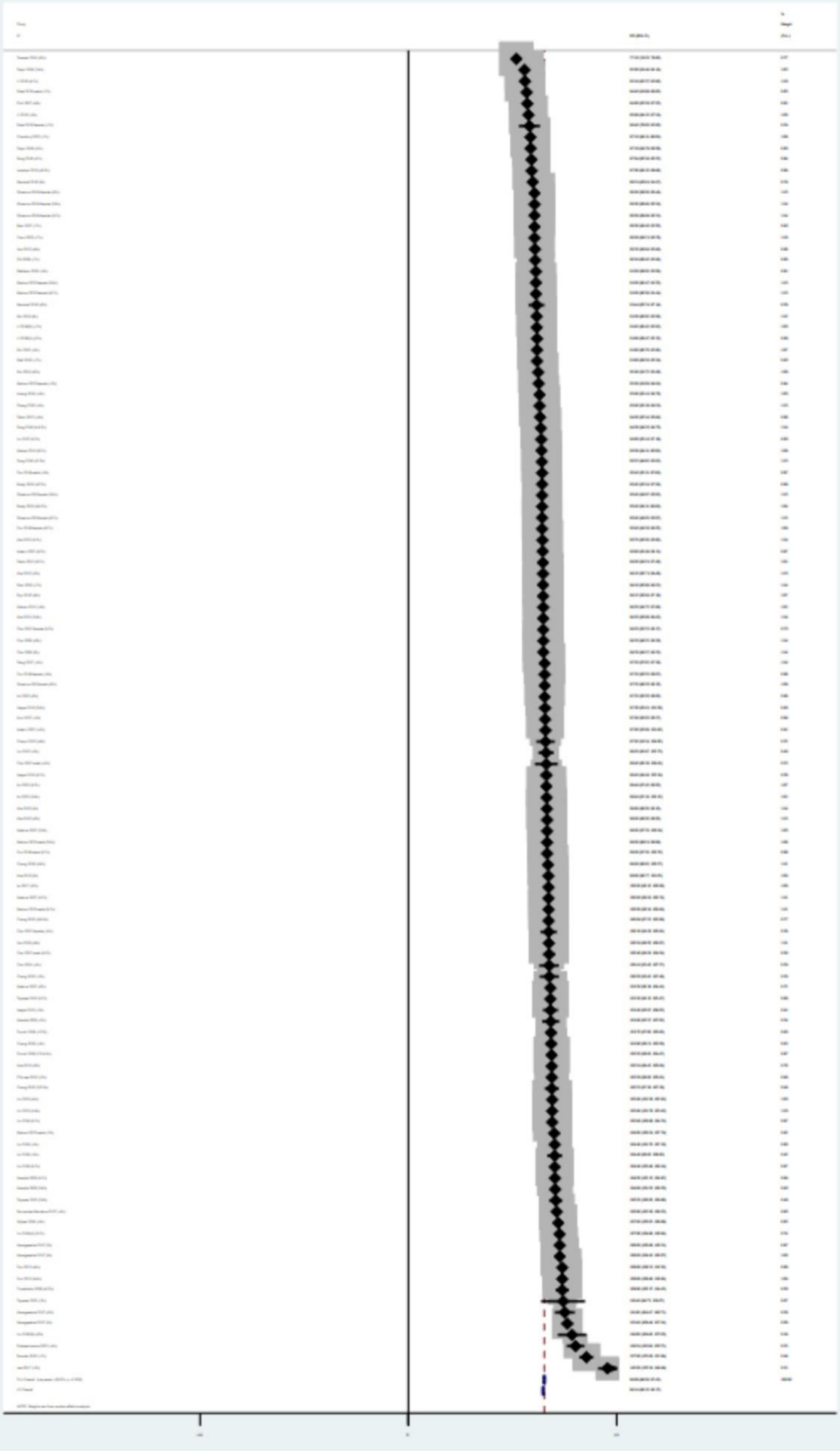

B

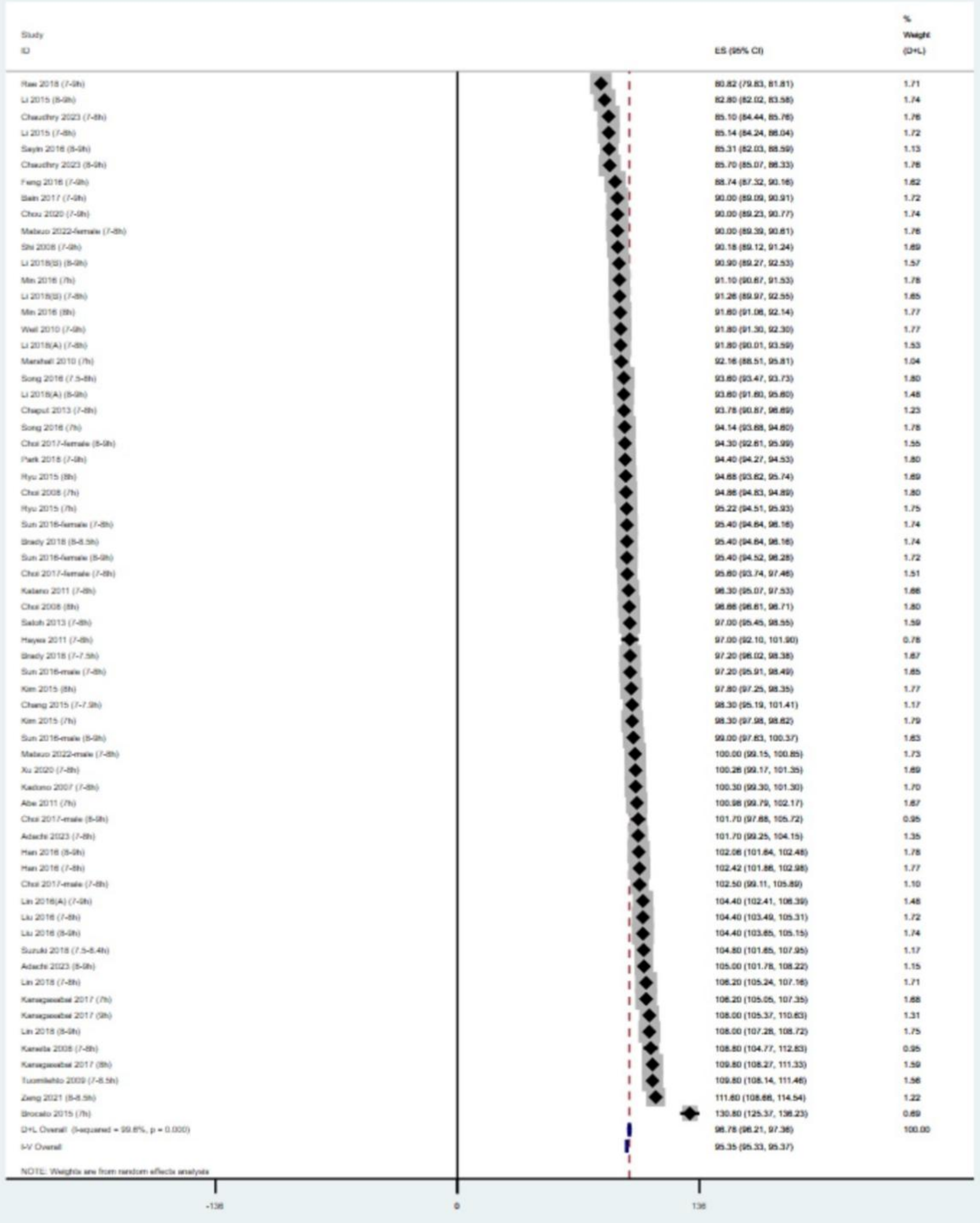
Study
ID
ES (95% CI)
%
Weight
(D+L)
NOTE: Weights are from random effects analysis
-136
0
136

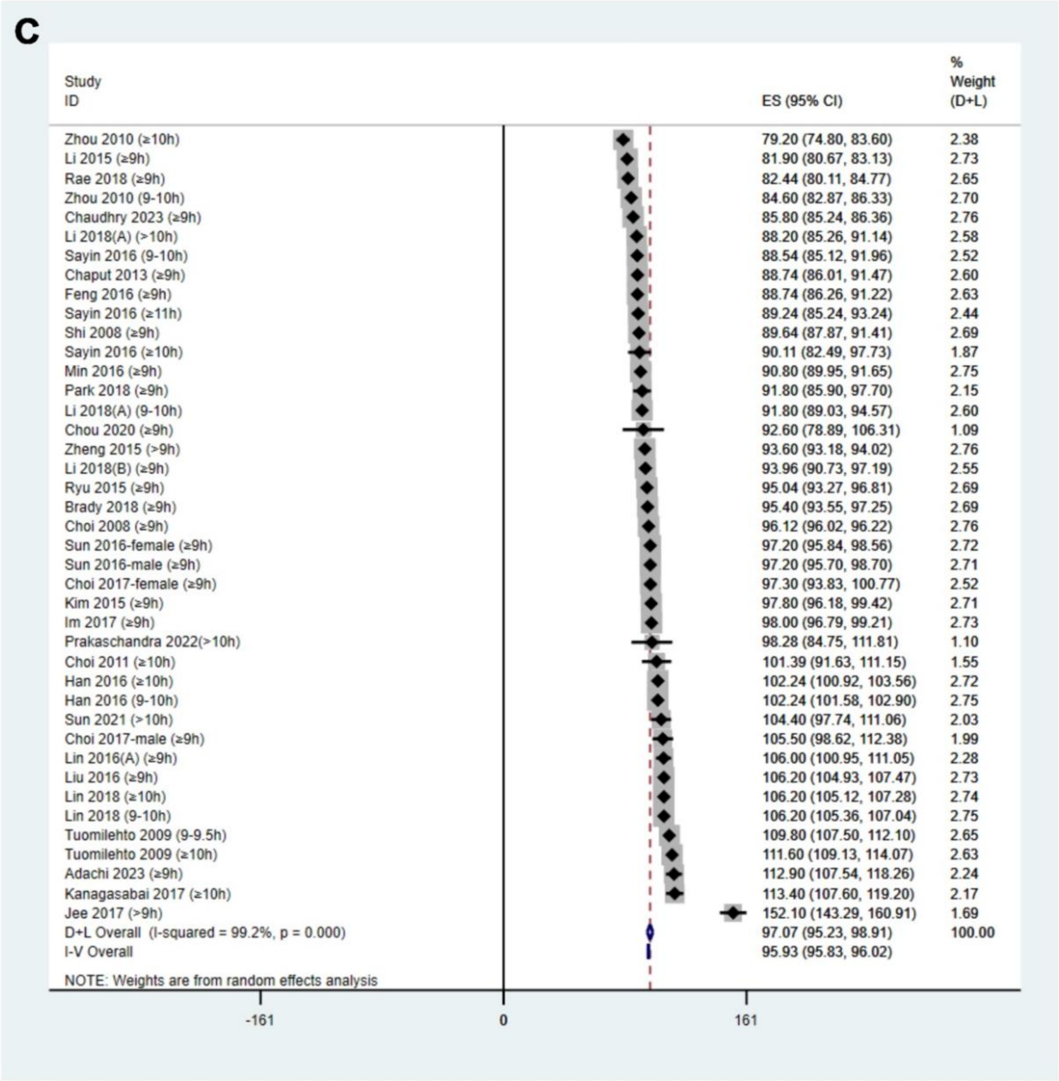

Supplemental Figure 8. Forest plots of fasting blood glucose (FBG) of different sleep duration in the single-arm meta-analysis. (A) short, (B) normal, (C) long sleep duration.

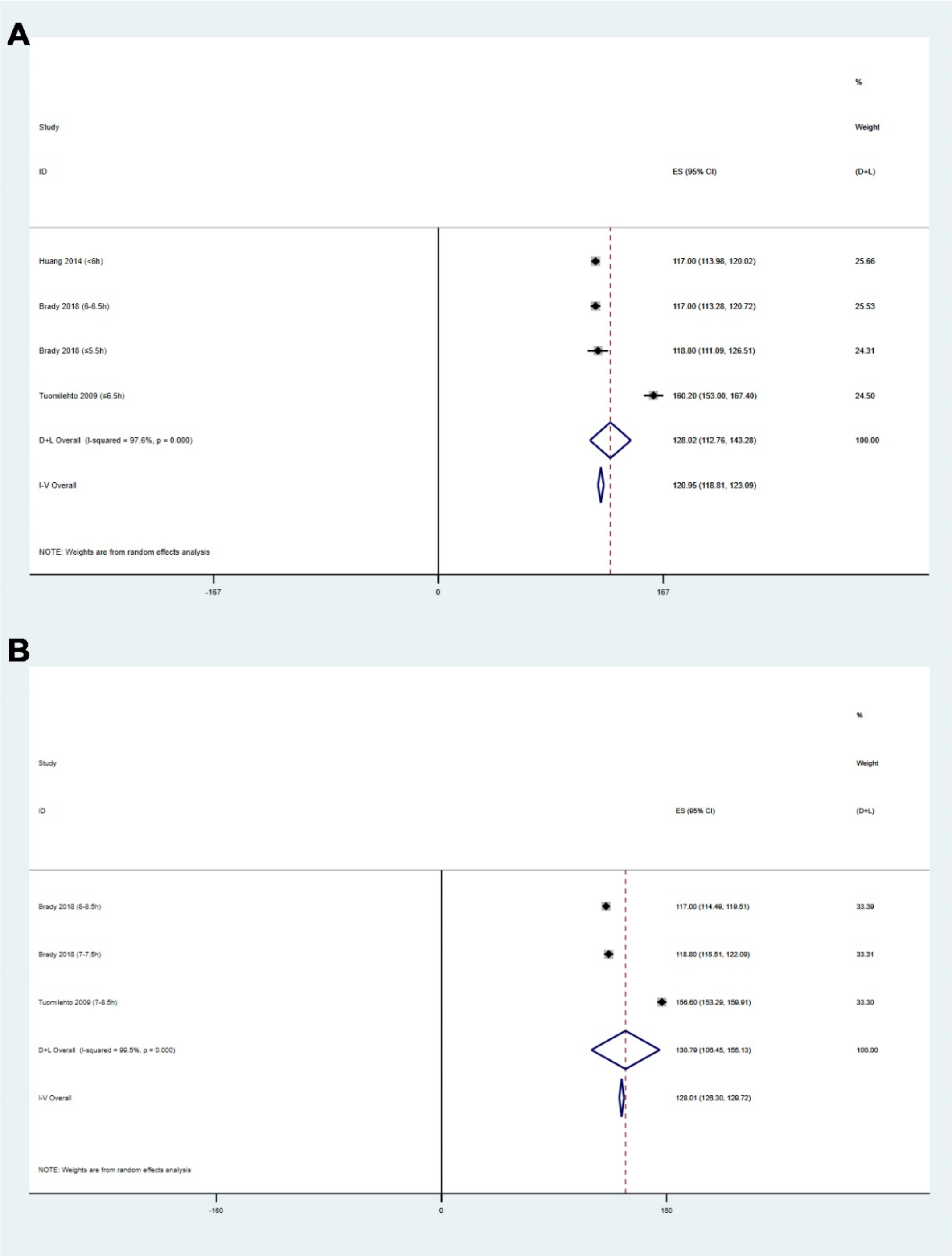
A
%
Study
Weight
ID
ES (95% CI)
(D+L)
Huang 2014 (<6h)
117.00 (113.98, 120.02)
25.66
Brady 2018 (6-6.5h)
117.00 (113.28, 120.72)
25.53
Brady 2018 (≤5.5h)
118.80 (111.09, 126.51)
24.31
Tuomilehto 2009 (≤6.5h)
160.20 (153.00, 167.40)
24.50
D+L Overall (I-squared = 97.6%, p = 0.000)
128.02 (112.76, 143.28)
100.00
I-V Overall
120.95 (118.81, 123.09)
NOTE: Weights are from random effects analysis
-167
0
167
B
%
Study
Weight
ID
ES (95% CI)
(D+L)
Brady 2018 (8-8.5h)
117.00 (114.49, 119.51)
33.39
Brady 2018 (7-7.5h)
118.80 (115.51, 122.09)
33.31
Tuomilehto 2009 (7-8.5h)
156.60 (153.29, 159.91)
33.30
D+L Overall (I-squared = 99.5%, p = 0.000)
130.79 (106.45, 155.13)
100.00
I-V Overall
128.01 (126.30, 129.72)
NOTE: Weights are from random effects analysis
-160
0
160

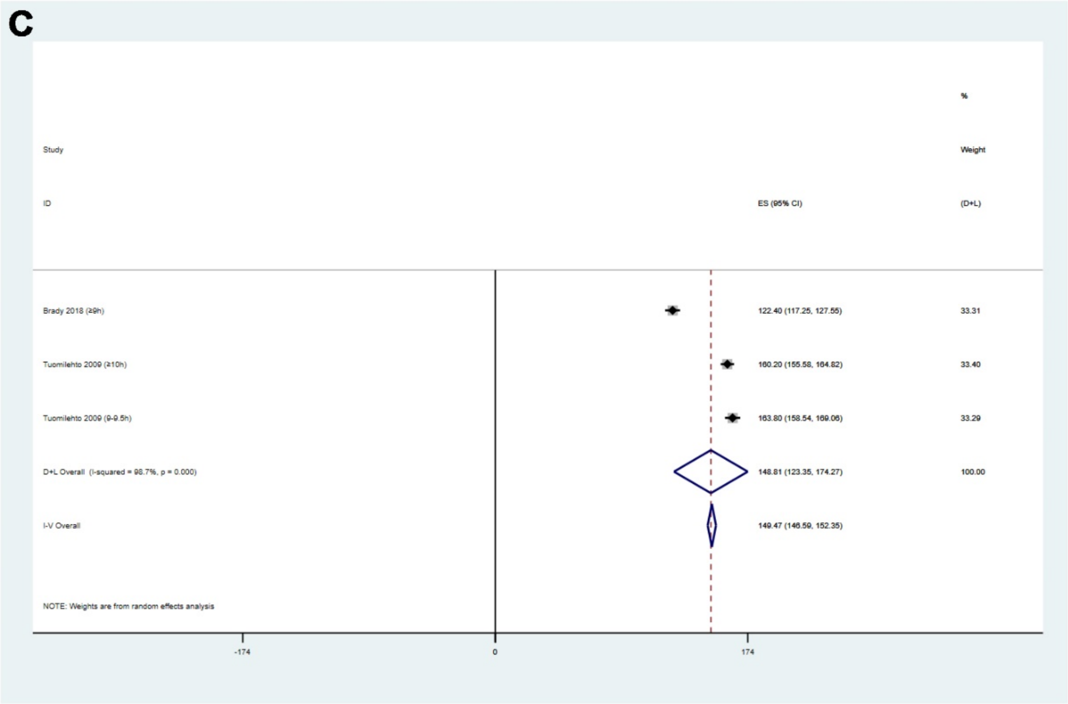

Supplemental Figure 9. Forest plots of 2h-glucose post-challenge (2hGlu) of different sleep duration in the single-arm meta-analysis. (A) short, (B) normal, (C) long sleep duration.

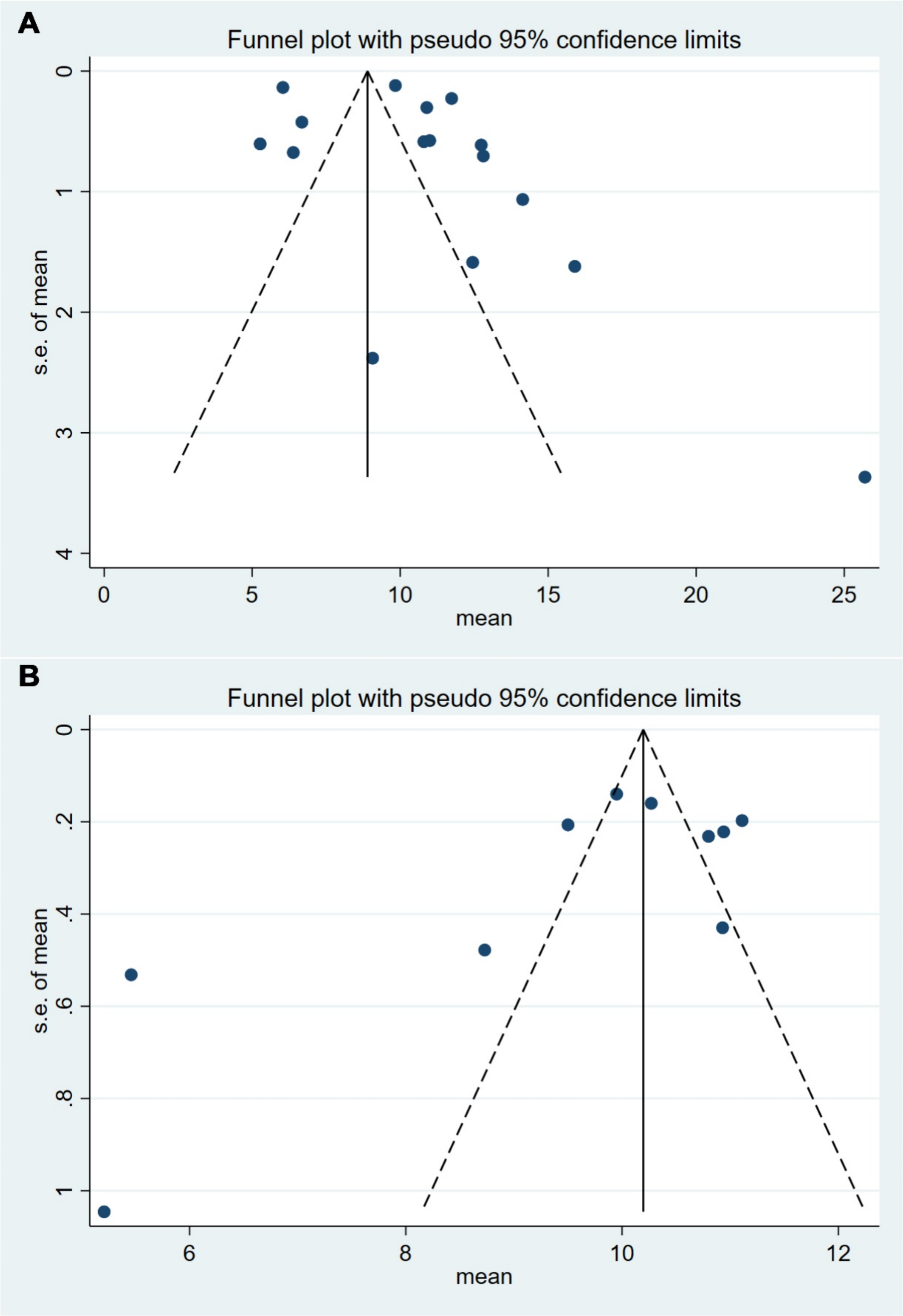
A
Funnel plot with pseudo 95% confidence limits
s.e. of mean
0
1
2
3
4
0
5
10
15
20
25
mean
B
Funnel plot with pseudo 95% confidence limits
s.e. of mean
0
.2
.4
.6
.8
1
6
8
10
12
mean

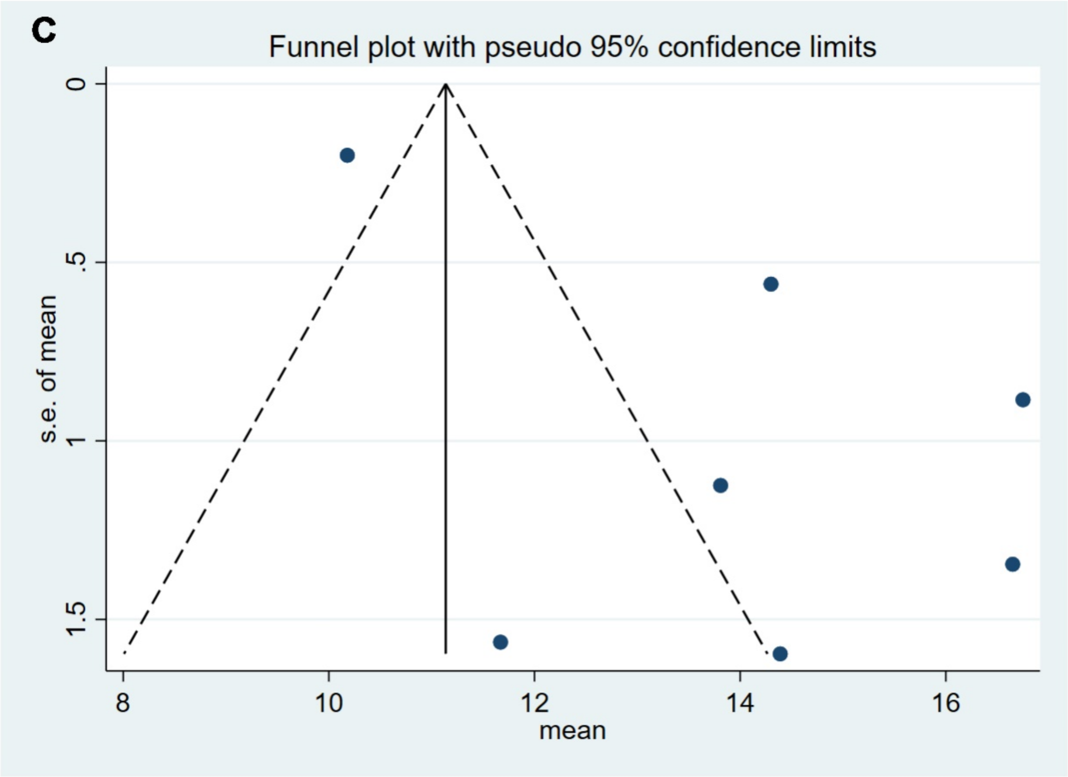

Supplemental Figure 10. Funnel plots of fasting insulin (FINS) of different sleep duration in the single-arm meta-analysis. (A) short, (B) normal, (C) long sleep duration.

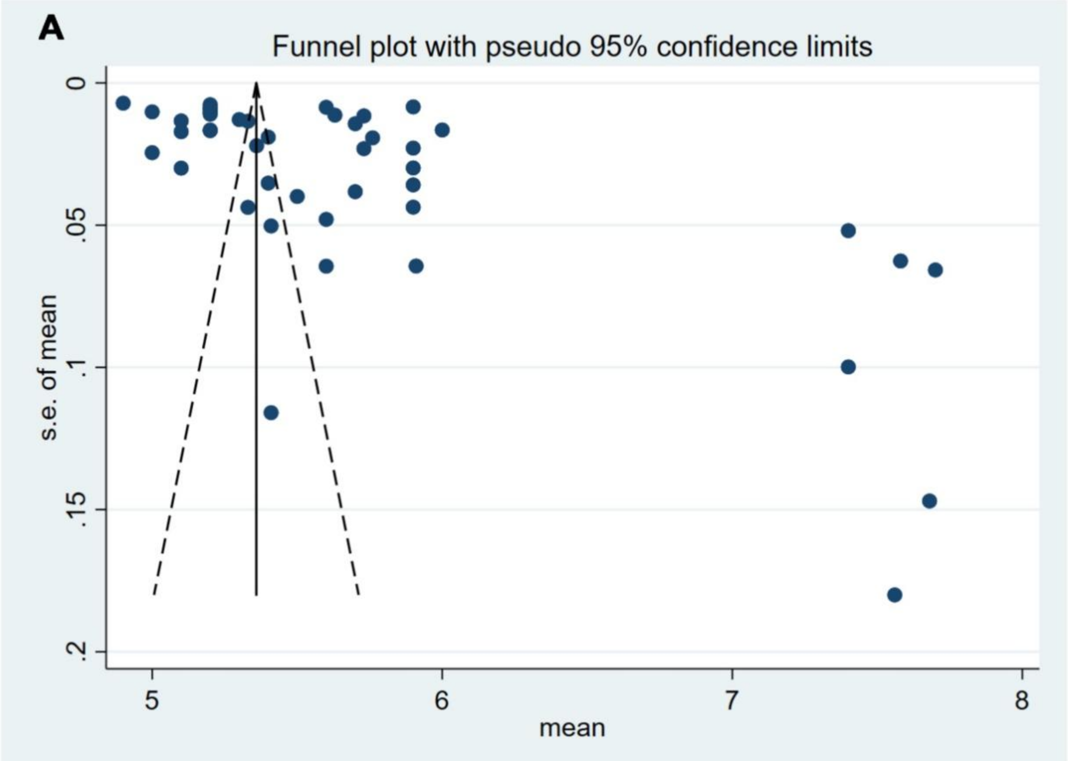
A
Funnel plot with pseudo 95% confidence limits
s.e. of mean
0
.05
.1
.15
.2
5
6
7
8
mean

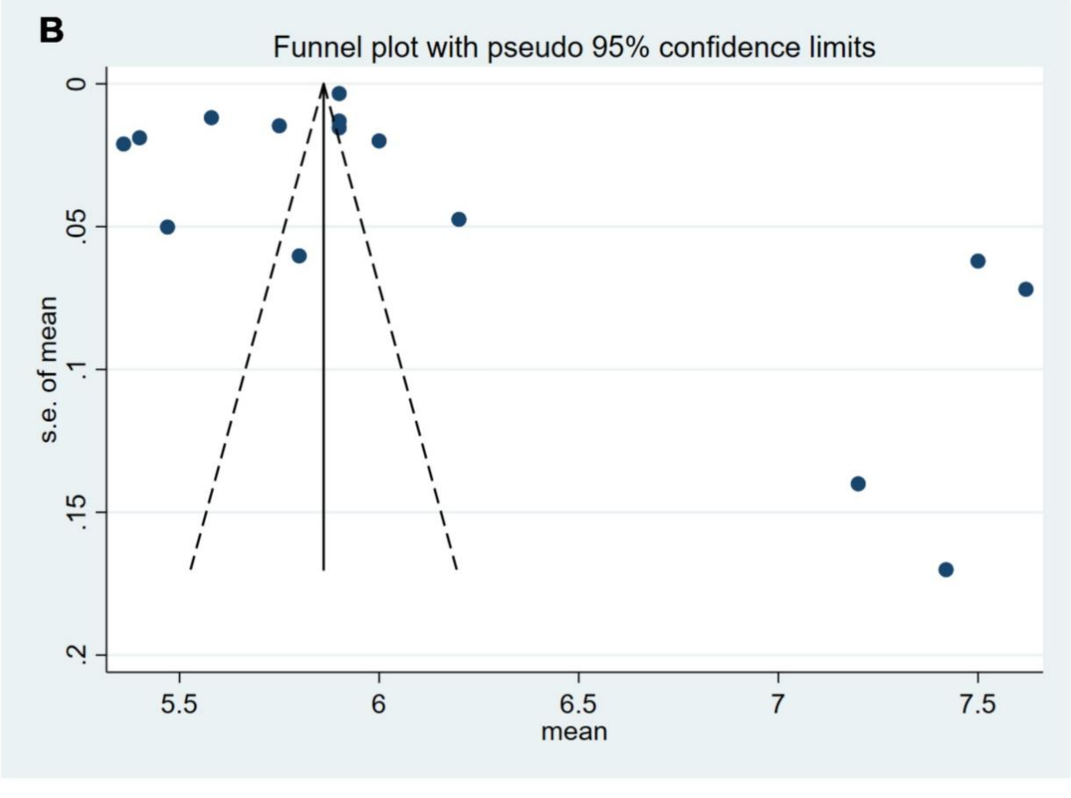
B
Funnel plot with pseudo 95% confidence limits
s.e. of mean
0
.05
.1
.15
.2
5.5
6
6.5
7
7.5
mean

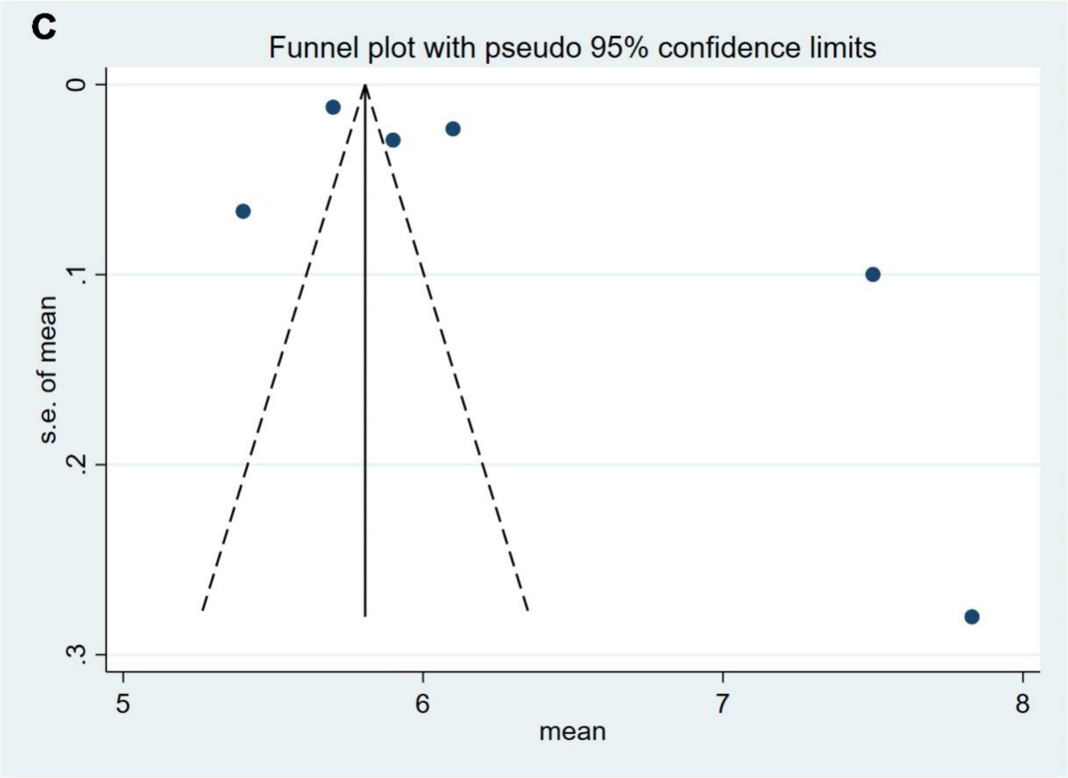

Supplemental Figure 11. Funnel plots of glycosylated hemoglobin (HbA1c) of different sleep duration in the single-arm meta-analysis. (A) short, (B) normal, (C) long sleep duration.

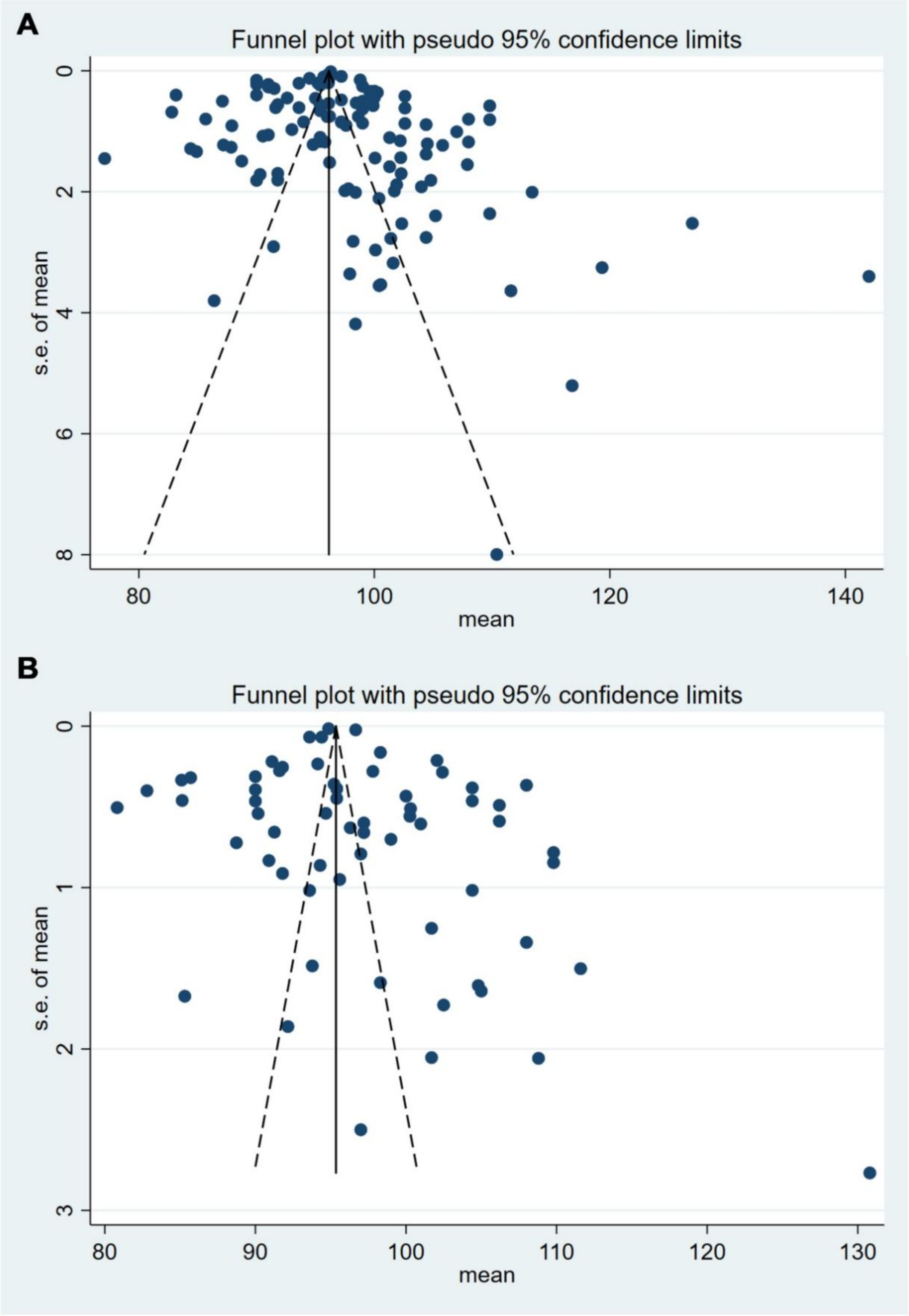

A
Funnel plot with pseudo 95% confidence limits
s.e. of mean
0
2
4
6
8
80
100
120
140
mean
B
Funnel plot with pseudo 95% confidence limits
s.e. of mean
0
1
2
3
80
90
100
110
120
130
mean

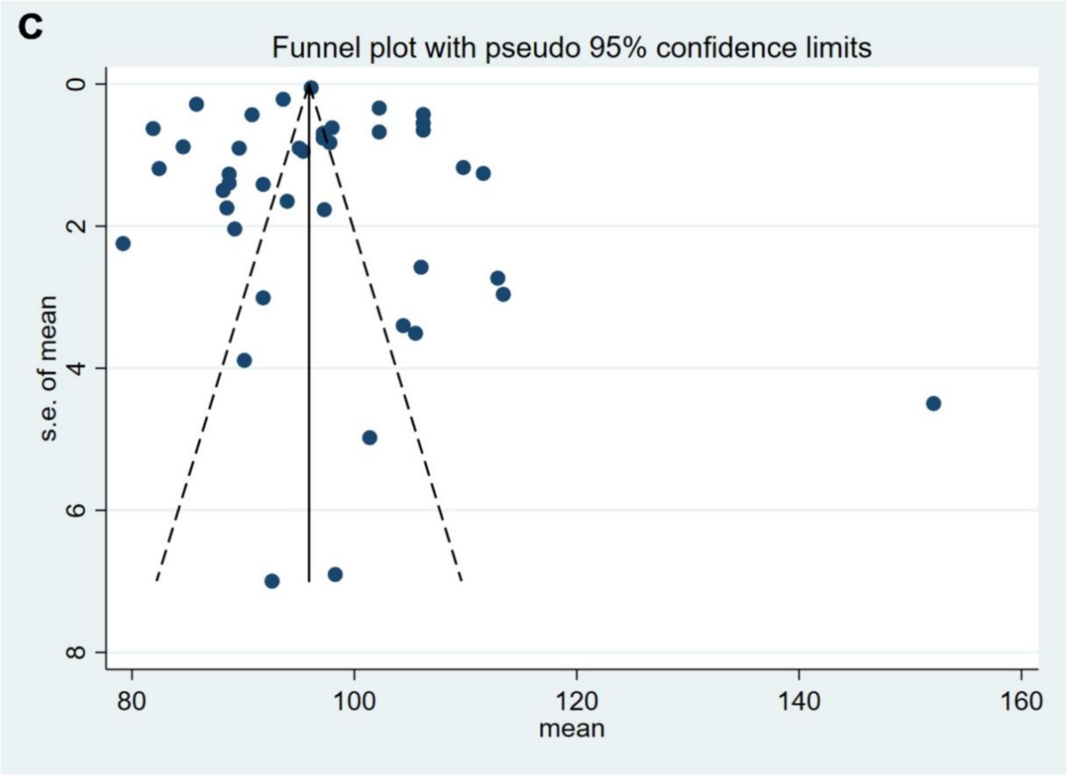

Supplemental Figure 12. Funnel plots of fasting blood glucose (FBG) of different sleep duration in the single-arm meta-analysis. (A) short, (B) normal, (C) long sleep duration.

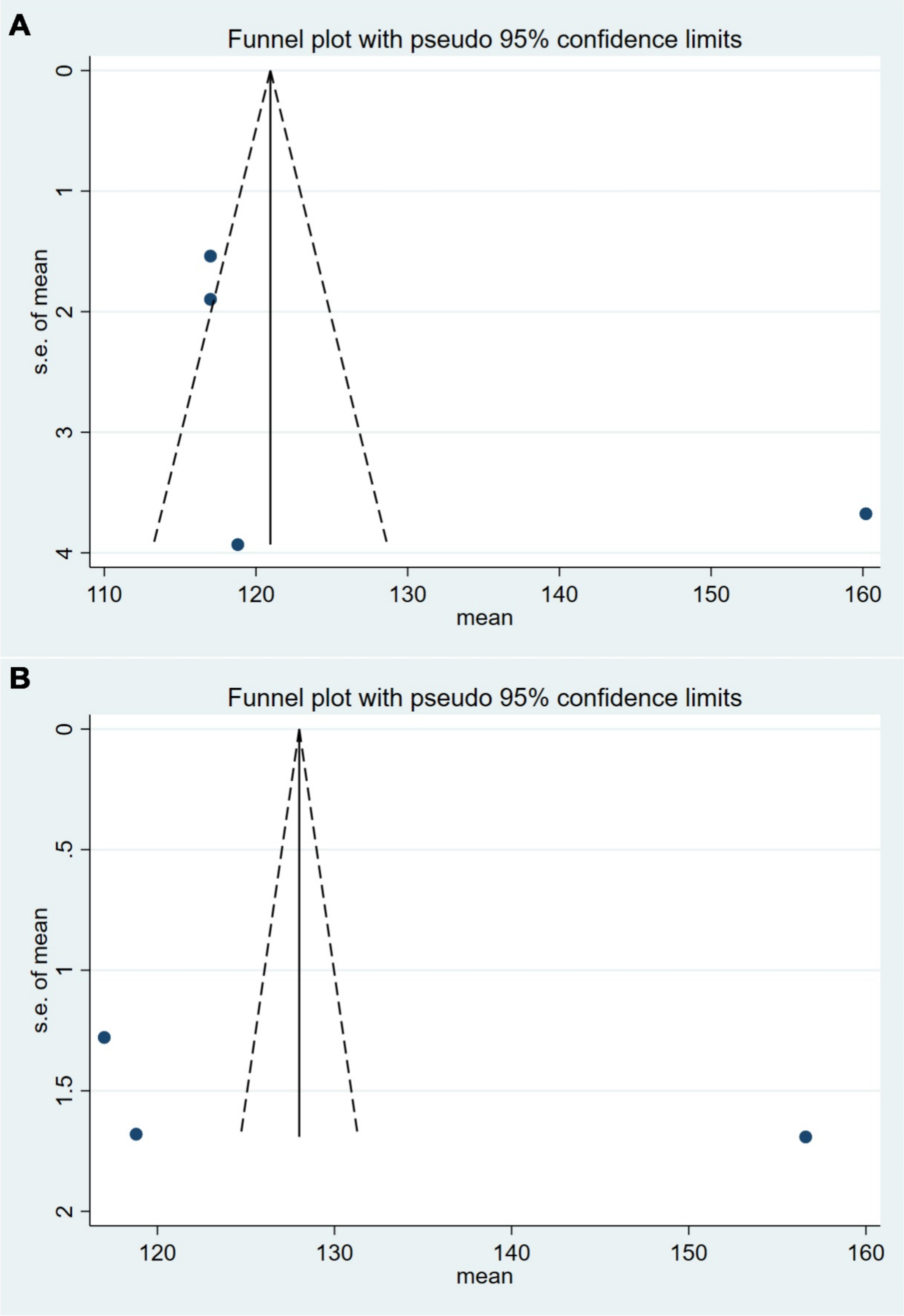
A
Funnel plot with pseudo 95% confidence limits
s.e. of mean
0
1
2
3
4
110
120
130
140
150
160
mean
B
Funnel plot with pseudo 95% confidence limits
s.e. of mean
0
.5
1
1.5
2
120
130
140
150
160
mean

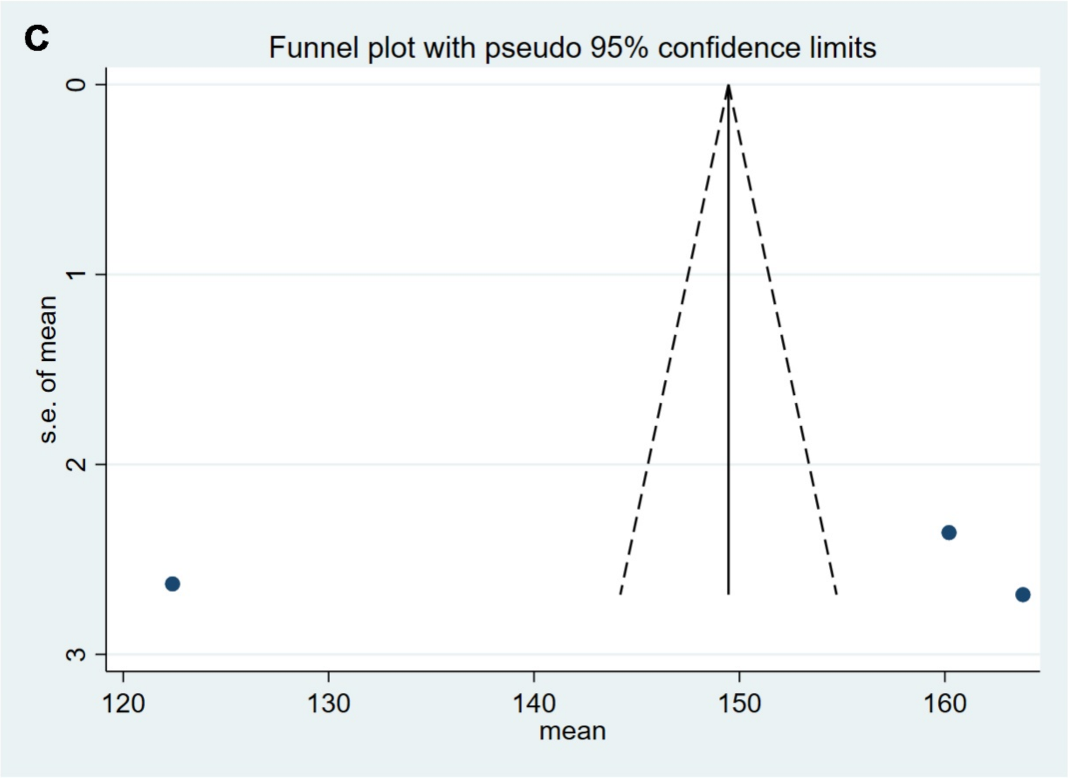

Supplemental Figure 13. Funnel plots of 2h-glucose post-challenge (2hGlu) of different sleep duration in the single-arm meta-analysis. (A) short, (B) normal, (C) long sleep duration.

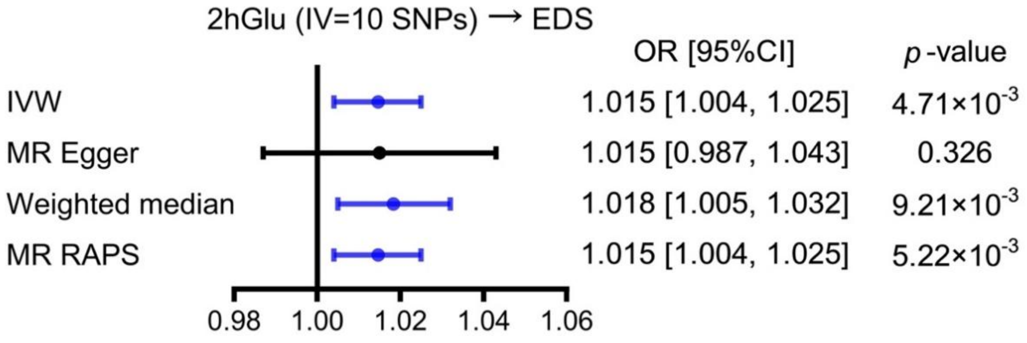

Supplemental Figure 14. Validation assessment of causal effects of 2hGlu levels on EDS. Outliers were identified by RadialMR and followed by removing before performing MR analysis of causality of 2hGlu levels on EDS again as sensitivity analysis. Abbreviation: 2hGlu: 2h-glucose post-challenge; IV: instrumental variable; SNP: single nucleotide polymorphism; OR: Odds ratios; CI: confidence intervals; IVW: Inverse variance weighting; RAPS: Robust Adjusted Profile Score.

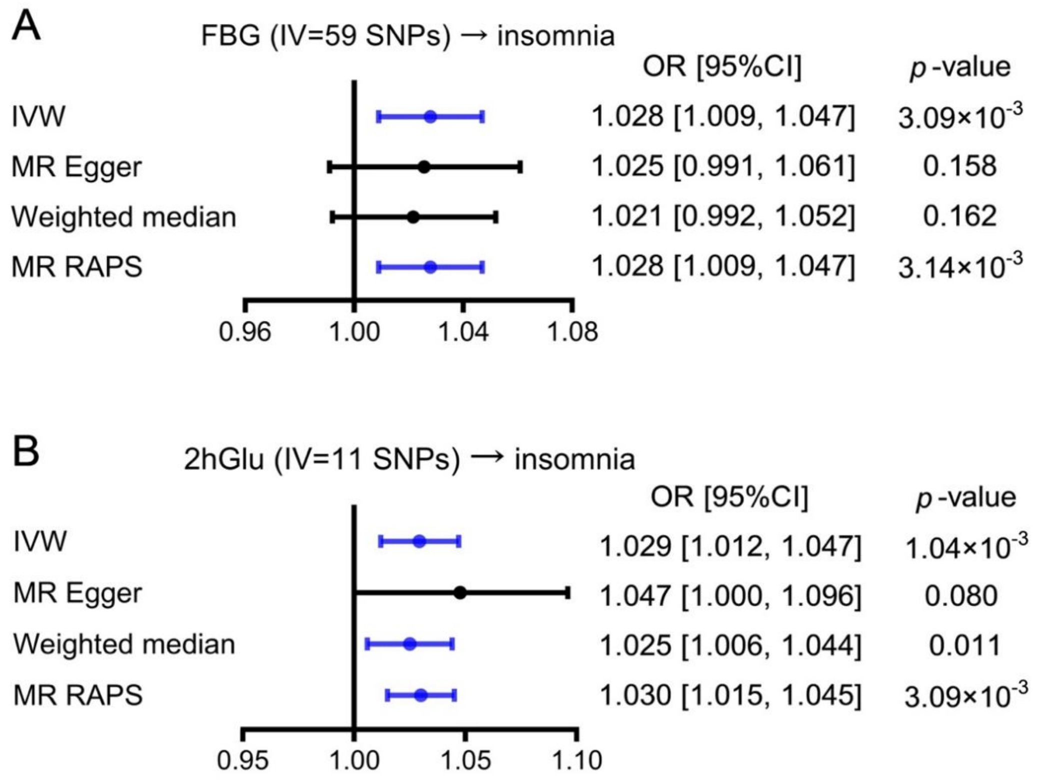

Supplemental Figure 15. Validation assessment of causal effects of FBG and 2hGlu levels on EDS. Outliers were identified by RadialMR and followed by removing before performing MR analysis of causality of FBG (A) and 2hGlu (B) levels on insomnia again as sensitivity analysis. Abbreviation: FBG: Fasting blood glucose; 2hGlu: 2h-glucose post-challenge; IV: instrumental variable; SNP: single nucleotide polymorphism; OR: Odds ratios; CI: confidence intervals; IVW: Inverse variance weighting; RAPS: Robust Adjusted Profile Score.

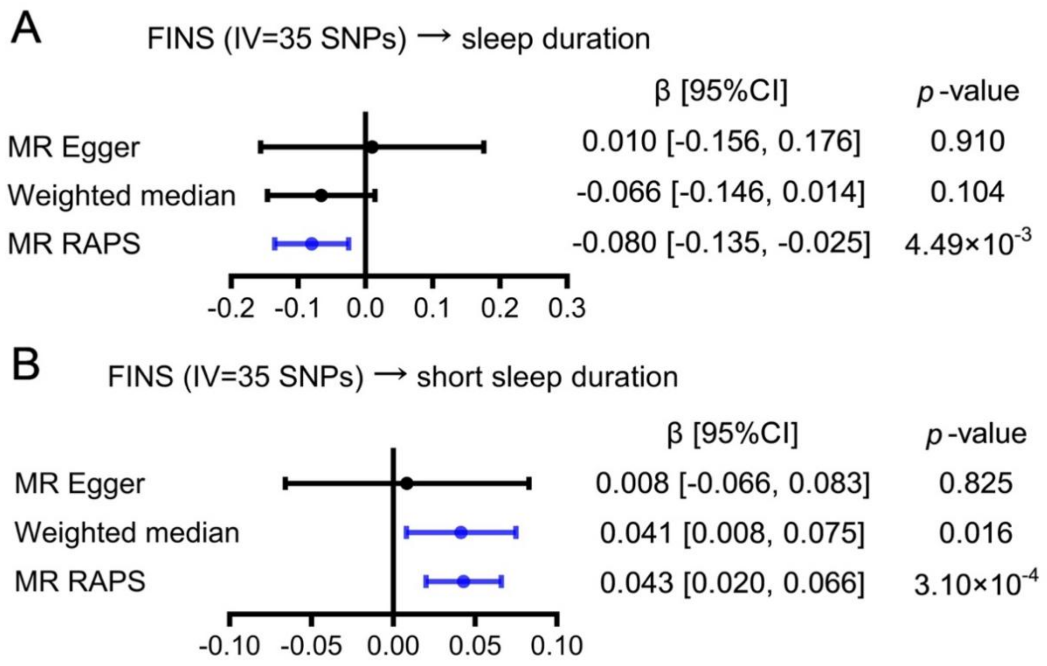

Supplemental Figure 16. Validation assessment of causal effects of FINS levels on sleep duration and short sleep duration. The causal effect of FINS levels on sleep duration and short sleep duration was estimated by sensitivity analysis. Abbreviation: FINS: Fasting insulin; IV: instrumental variable; SNP: single nucleotide polymorphism; β: Regression coefficient; CI: confidence intervals; RAPS: Robust Adjusted Profile Score.

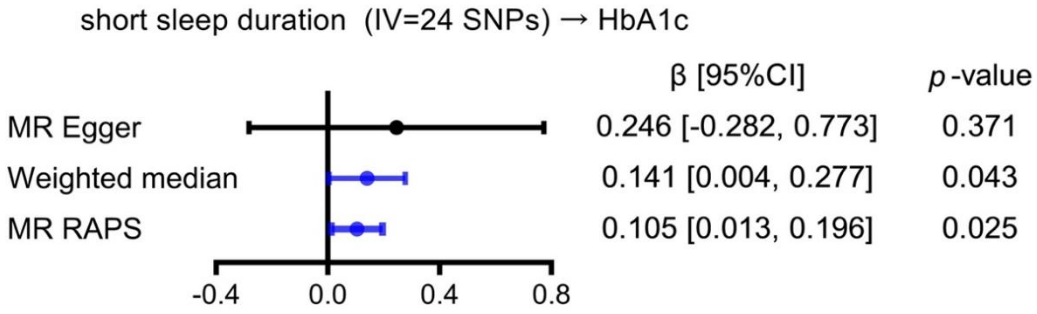

Supplemental Figure 17. Validation assessment of causal effects of short sleep duration on HBA1c levels. The causal effect of FINS levels on sleep duration and short sleep duration was estimated by sensitivity analysis. Abbreviation: HbA1c: Glycosylated hemoglobin; IV: instrumental variable; SNP: single nucleotide polymorphism; β: Regression coefficient; CI: confidence intervals; RAPS: Robust Adjusted Profile Score.

References

[1] X. Li, H. Huang, H. Xu, Y. Shi, Y. Qian, J. Zou, H. Yi, J. Guan, and S. Yin, Excessive daytime sleepiness, metabolic syndrome, and obstructive sleep apnea: two independent large cross-sectional studies and one interventional study. Respir Res 20 (2019) 276.

[2] J.F. Huang, L.D. Chen, Q.C. Lin, G.P. Chen, Y.H. Yu, J.C. Huang, and J.M. Zhao, The relationship between excessive daytime sleepiness and metabolic syndrome in severe obstructive sleep apnea syndrome. Clin Respir J 10 (2016) 714-721.

[3] D.K. Andaku, V. D'Almeida, G. Carneiro, S. Hix, S. Tufik, and S.M. Togeiro, Sleepiness, inflammation and oxidative stress markers in middle-aged males with obstructive sleep apnea without metabolic syndrome: a cross-sectional study. Respir Res 16 (2015) 3.

[4] J.H. Yu, J.H. Ahn, H.J. Yoo, J.A. Seo, S.G. Kim, K.M. Choi, S.H. Baik, D.S. Choi, C. Shin, and N.H. Kim, Obstructive sleep apnea with excessive daytime sleepiness is associated with non-alcoholic fatty liver disease regardless of visceral fat. Korean J Intern Med 30 (2015) 846-55.

[5] E.A. Pulixi, E. Tobaldini, P.M. Battezzati, P. D'Ingianna, V. Borroni, A.L. Fracanzani, M. Maggioni, S. Pelusi, M. Bulgheroni, M. Zuin, S. Fargion, N. Montano, and L. Valenti, Risk of obstructive sleep apnea with daytime sleepiness is associated with liver damage in non-morbidly obese patients with nonalcoholic fatty liver disease. PLoS One 9 (2014) e96349.

[6] M.R. Bonsignore, C. Esquinas, A. Barceló, M. Sanchez-de-la-Torre, A. Paternó, J. Duran-Cantolla, J.M. Marín, and F. Barbé, Metabolic syndrome, insulin

resistance and sleepiness in real-life obstructive sleep apnoea. Eur Respir J 39 (2012) 1136-43.

[7] E. Nena, P. Steiropoulos, N. Papanas, V. Tsara, C. Fitili, M.E. Froudarakis, E. Maltezos, and D. Bouros, Sleepiness as a marker of glucose deregulation in obstructive sleep apnea. Sleep Breath 16 (2012) 181-6.

[8] A. Barceló, F. Barbé, M. de la Peña, P. Martinez, J.B. Soriano, J. Piérola, and A.G. Agustí, Insulin resistance and daytime sleepiness in patients with sleep apnoea. Thorax 63 (2008) 946-50.

[9] C.K. O, B.W. Siu, V.W. Leung, Y.Y. Lin, C.Z. Ding, E.S. Lau, J. Cheung, A.O. Luk, E.Y. Chow, R.C. Ma, J.C. Chan, R.N. Chan, Y.K. Wing, and A.P. Kong, Association of insomnia with incident chronic cognitive impairment in older adults with type 2 diabetes mellitus: A prospective study of the Hong Kong Diabetes Register. J Diabetes Complications 37 (2023) 108598.

[10] W. Zhang, Q. Sun, B. Chen, M. Basta, C. Xu, and Y. Li, Insomnia symptoms are associated with metabolic syndrome in patients with severe psychiatric disorders. Sleep Med 83 (2021) 168-174.

[11] B. Xu, L. Ma, N. Zhang, W. Guo, L.M. Luo, C. Wang, Y. Jiang, and L.G. Liu, Increased microparticle levels in middle-aged and elderly patients with insomnia may be involved in the pathogenesis of arteriosclerosis. Int Angiol 39 (2020) 252-260.

[12] E.S. LeBlanc, N.X. Smith, G.A. Nichols, M.J. Allison, and G.N. Clarke, Insomnia is associated with an increased risk of type 2 diabetes in the clinical setting. BMJ Open Diabetes Res Care 6 (2018) e000604.

[13] J. Tschepp, C.J. Lauer, J. Wilde-Frenz, and T. Pollmächer, No Impaired Glucose Tolerance in Primary Insomnia Patients with Normal Results of Polysomnography. Front Neurol 8 (2017) 303.

[14] O.K. Ham, J. Kim, B.G. Lee, and E. Choi, Behavioral Characteristics and Cardiovascular Disease Risks Associated With Insomnia and Sleep Quality Among Middle-Aged Women in South Korea. Res Nurs Health 40 (2017) 206-217.

[15] A.J. Pyykkönen, B. Isomaa, A.K. Pesonen, J.G. Eriksson, L. Groop, T. Tuomi, and K. Räikkönen, Subjective sleep complaints are associated with insulin resistance in individuals without diabetes: The PPP-botnia study. Diabetes Care 35 (2012) 2271-2278.

[16] M. Keckeis, Z. Lattova, E. Maurovich-Horvat, P.A. Beitinger, S. Birkmann, C.J. Lauer, T.C. Wetter, J. Wilde-Frenz, and T. Pollmächer, Impaired glucose tolerance in sleep disorders. PLoS ONE 5 (2010).

[17] T. Abe, T. Aoki, S. Yata, and M. Okada, Sleep duration is significantly associated with carotid artery atherosclerosis incidence in a Japanese population. Atherosclerosis 217 (2011) 509-13.

[18] H. Adachi, A. Matsumura, M. Enomoto, A. Fukami, N. Morikawa, Y. Nohara, M. Yamamoto, H. Sato, T. Kakuma, and Y. Fukumoto, A J-shaped Relationship between Sleep Duration and the Risk of Insulin Resistance in a General Japanese Population. Internal Medicine 62 (2023) 511-517.

[19] M. Aziz, C.U. Osondu, A. Younus, R. Malik, M. Rouseff, S. Das, H. Guzman, W. Maziak, S. Virani, T. Feldman, A.S. Agatston, E. Veledar, E.C. Aneni, and K. Nasir, The Association of Sleep Duration and Morbid Obesity in a Working Population: The Baptist Health South Florida Employee Study. Metab Syndr Relat Disord 15 (2017) 59-62.

[20] A.R. Bain, B.R. Weil, K.J. Diehl, J.J. Greiner, B.L. Stauffer, and C.A. DeSouza, Insufficient sleep is associated with impaired nitric oxide-mediated endothelium-dependent vasodilation. Atherosclerosis 265 (2017) 41-46.

[21] E.M. Brady, D.H. Bodicoat, A.P. Hall, K. Khunti, T. Yates, C. Edwardson, and M.J. Davies, Sleep duration, obesity and insulin resistance in a multi-ethnic UK population at high risk of diabetes. Diabetes Res Clin Pract 139 (2018) 195-202.

[22] J. Brocato, F. Wu, Y. Chen, M. Shamy, M.A. Alghamdi, M.I. Khoder, A.A. Alkhatim, M.H. Abdou, and M. Costa, Association between sleeping hours and cardiometabolic risk factors for metabolic syndrome in a Saudi Arabian population. BMJ Open 5 (2015) e008590.

[23] J.H. Chang, P.T. Huang, Y.K. Lin, C.E. Lin, C.M. Lin, Y.H. Shieh, and Y.C. Lin, Association between sleep duration and sleep quality, and metabolic syndrome in Taiwanese police officers. Int J Occup Med Environ Health 28 (2015) 1011-23.

[24] Y.C. Chang, C.H. Chang, Y.T. Huang, Y.C. Yeh, M.L. Hsieh, W.S. Yang, and Y.K. Tu, Sleep Duration and Proteinuria Progression: A Population-Based Cohort Study. Am J Nephrol 49 (2019) 41-51.

[25] J.P. Chaput, J. McNeil, J.P. Després, C. Bouchard, and A. Tremblay, Short sleep duration as a risk factor for the development of the metabolic syndrome in adults. Prev Med 57 (2013) 872-7.

[26] B.A. Chaudhry, M.S. Brian, and J.S. Morrell, The Relationship between Sleep Duration and Metabolic Syndrome Severity Scores in Emerging Adults. Nutrients 15 (2023).

[27] K.M. Choi, J.S. Lee, H.S. Park, S.H. Baik, D.S. Choi, and S.M. Kim, Relationship between sleep duration and the metabolic syndrome: Korean National Health and Nutrition Survey 2001. Int J Obes (Lond) 32 (2008) 1091-7.

[28] J.K. Choi, M.Y. Kim, J.K. Kim, J.K. Park, S.S. Oh, S.B. Koh, and A. Eom, Association between short sleep duration and high incidence of metabolic syndrome in midlife women. Tohoku J Exp Med 225 (2011) 187-93.

[29] H. Choi, H.C. Kim, J.Y. Lee, J.M. Lee, D.P. Choi, and I. Suh, Sleep duration and chronic kidney disease: The Korean Genome and Epidemiology Study (KoGES)-Kangwha study. Korean J Intern Med 32 (2017) 323-334.

[30] Y.T. Chou, C.H. Li, W.C. Shen, Y.C. Yang, F.H. Lu, J.S. Wu, and C.J. Chang, Association of sleep quality and sleep duration with serum uric acid levels in adults. PLoS One 15 (2020) e0239185.

[31] C. D'Aurea, D. Poyares, R.D. Piovezan, G. Passos, S. Tufik, and M.T. Mello, Objective short sleep duration is associated with the activity of the

hypothalamic-pituitary-adrenal axis in insomnia. Arq Neuropsiquiatr 73 (2015) 516-9.

[32] H.B. Deng, T. Tam, B.C. Zee, R.Y. Chung, X. Su, L. Jin, T.C. Chan, L.Y. Chang, E.K. Yeoh, and X.Q. Lao, Short Sleep Duration Increases Metabolic Impact in Healthy Adults: A Population-Based Cohort Study. Sleep 40 (2017).

[33] D. Feng, J. Zhang, J. Fu, H. Wu, Y. Wang, L. Li, Y. Zhao, M. Li, and S. Gao, Association between sleep duration and cardiac structure in youths at risk for metabolic syndrome. Sci Rep 6 (2016) 39017.

[34] J. Fernandez-Mendoza, F. He, C. LaGrotte, A.N. Vgontzas, D. Liao, and E.O. Bixler, Impact of the Metabolic Syndrome on Mortality is Modified by Objective Short Sleep Duration. J Am Heart Assoc 6 (2017).

[35] J. Flint, S.V. Kothare, M. Zihlif, E. Suarez, R. Adams, A. Legido, and F. De Luca, Association between inadequate sleep and insulin resistance in obese children. J Pediatr 150 (2007) 364-9.

[36] A.L. Hayes, F. Xu, D. Babineau, and S.R. Patel, Sleep duration and circulating adipokine levels. Sleep 34 (2011) 147-52.

[37] H. Baoying, C. Hongjie, Q. Changsheng, W. Peijian, L. Qingfei, L. Yinghua, H. Huibin, L. Jixing, L. Liantao, C. Ling, T. Kaka, C. Zichun, L. Lixiang, L. Jieli, B. Yufang, N. Guang, Z. Penli, W. Junping, and C. Gang, Association of napping and night-time sleep with impaired glucose regulation, insulin resistance and glycated haemoglobin in Chinese middle-aged adults with no diabetes: a cross-sectional study. BMJ Open 4 (2014) e004419.

[38] E. Im, and G.S. Kim, Relationship between sleep duration and Framingham cardiovascular risk score and prevalence of cardiovascular disease in Koreans. Medicine (Baltimore) 96 (2017) e7744.

[39] S. Javaheri, A. Storfer-Isser, C.L. Rosen, and S. Redline, Association of short and long sleep durations with insulin sensitivity in adolescents. J Pediatr 158 (2011) 617-23.

[40] D. Jee, N. Keum, S. Kang, and J.G. Arroyo, Sleep and diabetic retinopathy. Acta Ophthalmol 95 (2017) 41-47.

[41] M. Kadono, G. Hasegawa, M. Shigeta, A. Nakazawa, M. Ueda, M. Fukui, T. Yoshikawa, and N. Nakamura, Joint effect of alcohol and usual sleep duration on the risk of dysglycemia. Sleep 30 (2007) 1341-7.

[42] T. Kanagasabai, and J.P. Chaput, Sleep duration and the associated cardiometabolic risk scores in adults. Sleep Health 3 (2017) 195-203.

[43] Y. Kaneita, M. Uchiyama, N. Yoshiike, and T. Ohida, Associations of usual sleep duration with serum lipid and lipoprotein levels. Sleep 31 (2008) 645-52.

[44] S. Katano, Y. Nakamura, A. Nakamura, Y. Murakami, T. Tanaka, T. Takebayashi, A. Okayama, K. Miura, T. Okamura, and H. Ueshima, Association of short sleep duration with impaired glucose tolerance or diabetes mellitus. J Diabetes Investig 2 (2011) 366-72.

[45] C.W. Kim, K.E. Yun, H.S. Jung, Y. Chang, E.S. Choi, M.J. Kwon, E.H. Lee, E.J. Woo, N.H. Kim, H. Shin, and S. Ryu, Sleep duration and quality in relation to

non-alcoholic fatty liver disease in middle-aged workers and their spouses. J Hepatol 59 (2013) 351-7.

[46] C.W. Kim, Y. Chang, D. Zhao, M. Cainzos-Achirica, S. Ryu, H.S. Jung, K.E. Yun, Y. Choi, J. Ahn, Y. Zhang, S. Rampal, Y. Baek, J.A. Lima, H. Shin, E. Guallar, J. Cho, and E. Sung, Sleep Duration, Sleep Quality, and Markers of Subclinical Arterial Disease in Healthy Men and Women. Arterioscler Thromb Vasc Biol 35 (2015) 2238-45.

[47] X. Li, L. Lin, L. Lv, X. Pang, S. Du, W. Zhang, G. Na, H. Ma, Q. Zhang, S. Jiang, H. Deng, T. Han, C. Sun, and Y. Li, U-shaped relationships between sleep duration and metabolic syndrome and metabolic syndrome components in males: a prospective cohort study. Sleep Med 16 (2015) 949-54.

[48] J.W.S. Li, C.T. Au, K.C.C. Chan, P. Chook, Y.K. Wing, and A.M. Li, Short Sleep Duration Is Weakly Associated with Carotid Intima-Media Thickness in Adolescents. J Pediatr 195 (2018) 80-84.

[49] X. Li, X. Pang, Z. Liu, Q. Zhang, C. Sun, J. Yang, and Y. Li, Joint effect of less than 1 h of daytime napping and seven to 8 h of night sleep on the risk of stroke. Sleep Med 52 (2018) 180-187.

[50] C.L. Lin, Y.H. Tsai, and M.C. Yeh, Associations between sleep duration and type 2 diabetes in Taiwanese adults: A population-based study. J Formos Med Assoc 115 (2016) 779-85.

[51] C.L. Lin, Y.H. Tsai, and M.C. Yeh, The relationship between insomnia with short sleep duration is associated with hypercholesterolemia: a cross-sectional study. J Adv Nurs 72 (2016) 339-47.

[52] P.M.D. Lin, K.T. Chang, Y.A. Lin, I.S. Tzeng, H.H. Chuang, and J.Y. Chen, Association between self-reported sleep duration and serum lipid profile in a middle-aged and elderly population in Taiwan: a community-based, cross-sectional study. BMJ Open 7 (2017) e015964.

[53] M. Lin, Q. Su, J. Wen, S. Wei, J. Yao, H. Huang, J. Liang, L. Li, W. Lin, L. Lin, J. Lu, Y. Bi, W. Wang, G. Ning, and G. Chen, Self-reported sleep duration and daytime napping are associated with renal hyperfiltration in general population. Sleep Breath 22 (2018) 223-232.

[54] L. Lin, C. Lu, W. Chen, and V.Y. Guo, Daytime Napping and Nighttime Sleep Duration with Incident Diabetes Mellitus: A Cohort Study in Chinese Older Adults. Int J Environ Res Public Health 18 (2021).

[55] C. Liu, R. Zhong, J. Lou, A. Pan, Y. Tang, J. Chang, J. Ke, J. Li, J. Yuan, Y. Wang, W. Chen, H. Guo, S. Wei, Y. Liang, X. Zhang, M. He, F.B. Hu, T. Wu, P. Yao, and X. Miao, Nighttime sleep duration and risk of nonalcoholic fatty liver disease: the Dongfeng-Tongji prospective study. Ann Med 48 (2016) 468-476.

[56] N.S. Marshall, R.R. Grunstein, M. Peltonen, K. Stenlof, J. Hedner, and L.V. Sjostrom, Changes in sleep duration and changes in weight in obese patients: The swedish obese subjects study. Sleep and Biological Rhythms 8 (2010) 63-71.

[57] R. Matsuo, S. Tani, N. Matsumoto, and Y. Okumura, Assessment of sex differences in associations between sleep duration and lipid/glucose

metabolism in urban Japan: a cross-sectional study. Heart Vessels 37 (2022) 1583-1595.

[58] H. Min, Y.J. Um, B.S. Jang, D. Shin, E. Choi, S.M. Park, and K. Lee, Association between Sleep Duration and Measurable Cardiometabolic Risk Factors in Healthy Korean Women: The Fourth and Fifth Korean National Health and Nutrition Examination Surveys (KNHANES IV and V). Int J Endocrinol 2016 (2016) 3784210.

[59] S. Niijima, M. Nagai, S. Hoshide, M. Takahashi, M. Shimpo, and K. Kario, Long sleep duration: a nonconventional indicator of arterial stiffness in Japanese at high risk of cardiovascular disease: the J-HOP study. J Am Soc Hypertens 10 (2016) 429-37.

[60] T. Okamura, Y. Hashimoto, M. Hamaguchi, A. Obora, T. Kojima, and M. Fukui, Short sleep duration is a risk of incident nonalcoholic fatty liver disease: a population-based longitudinal study. J Gastrointestin Liver Dis 28 (2019) 73-81.

[61] S.K. Park, J.Y. Jung, C.M. Oh, R.S. McIntyre, and J.H. Lee, Association Between Sleep Duration, Quality and Body Mass Index in the Korean Population. J Clin Sleep Med 14 (2018) 1353-1360.

[62] M.C. Patel, W.A. Shaikh, and S.K. Singh, Association of sleep duration with blood glucose level of Gujarati Indian adolescents. Indian J Physiol Pharmacol 56 (2012) 229-33.

[63] D.R. Prakaschandra, and D.P. Naidoo, The association of cardiometabolic disorders with sleep duration: a cross-sectional study. African Health Sciences 22 (2022) 273-283.

[64] L. Rafalson, R.P. Donahue, S. Stranges, M.J. Lamonte, J. Dmochowski, J. Dorn, and M. Trevisan, Short sleep duration is associated with the development of impaired fasting glucose: the Western New York Health Study. Ann Epidemiol 20 (2010) 883-9.

[65] J.Y. Ryu, J.S. Lee, H.C. Hong, H.Y. Choi, H.J. Yoo, J.A. Seo, S.G. Kim, N.H. Kim, S.H. Baik, D.S. Choi, and K.M. Choi, Association between body size phenotype and sleep duration: Korean National Health and Nutrition Examination Survey V (KNHANES V). Metabolism 64 (2015) 460-6.

[66] H. Satoh, J. Nishihira, T. Wada, S. Fujii, and H. Tsutui, The relation between habitual sleep duration and blood pressure values in Japanese male subjects. Environ Health Prev Med 18 (2013) 215-20.

[67] F.K. Sayin, and M. Buyukinan, Sleep Duration and Media Time Have a Major Impact on Insulin Resistance and Metabolic Risk Factors in Obese Children and Adolescents. Child Obes 12 (2016) 272-8.

[68] Z. Shi, M. McEvoy, J. Luu, and J. Attia, Dietary fat and sleep duration in Chinese men and women. Int J Obes (Lond) 32 (2008) 1835-40.

[69] Q. Song, X. Liu, W. Zhou, X. Wang, and S. Wu, Short-term changes in sleep duration and risk of type 2 diabetes: Kailuan prospective study. Medicine (Baltimore) 95 (2016) e5363.

[70] X.M. Sun, S. Yao, S.J. Hu, Z.Y. Liu, Y.J. Yang, Z.Y. Yuan, W.M. Ye, L. Jin, and X.F. Wang, Short sleep duration is associated with increased risk of pre-hypertension and hypertension in Chinese early middle-aged females. Sleep Breath 20 (2016) 1355-1362.

[71] H. Sun, K. Qin, C. Zou, H.H. Wang, C. Lu, W. Chen, and V.Y. Guo, The association of nighttime sleep duration and quality with chronic kidney disease in middle-aged and older Chinese: a cohort study. Sleep Med 86 (2021) 25-31.

[72] S. Suzuki, H. Arima, S. Miyazaki, A. Fujiyoshi, A. Kadota, N. Takashima, T. Hisamatsu, S. Kadowaki, M. Zaid, S. Torii, M. Horie, K. Murata, K. Miura, and H. Ueshima, Self-reported Sleep Duration and Subclinical Atherosclerosis in a General Population of Japanese Men. J Atheroscler Thromb 25 (2018) 186-198.

[73] E.M. Taveras, S.L. Rifas-Shiman, J.W. Rich-Edwards, E.P. Gunderson, A.M. Stuebe, and C.S. Mantzoros, Association of maternal short sleep duration with adiposity and cardiometabolic status at 3 years postpartum. Obesity (Silver Spring) 19 (2011) 171-8.

[74] Y. Toyama, K. Chin, Y. Chihara, M. Takegami, K.I. Takahashi, K. Sumi, T. Nakamura, Y. Nakayama-Ashida, I. Minami, S. Horita, Y. Oka, T. Wakamura, S.I. Fukuhara, M. Mishima, and H. Kadotani, Association between sleep apnea, sleep duration, and serum lipid profile in an urban, male, working population in Japan. Chest 143 (2013) 720-728.

[75] H. Tuomilehto, M. Peltonen, M. Partinen, G. Lavigne, J.G. Eriksson, C. Herder, S. Aunola, S. Keinänen-Kiukaanniemi, P. Ilanne-Parikka, M. Uusitupa, J. Tuomilehto, and J. Lindström, Sleep duration, lifestyle intervention, and incidence of type 2 diabetes in impaired glucose tolerance: The Finnish Diabetes Prevention Study. Diabetes Care 32 (2009) 1965-71.

[76] B.R. Weil, M.L. Mestek, C.M. Westby, G.P. Van Guilder, J.J. Greiner, B.L. Stauffer, and C.A. DeSouza, Short sleep duration is associated with enhanced endothelin-1 vasoconstrictor tone. Can J Physiol Pharmacol 88 (2010) 777-81.

[77] M.C. Wu, Y.C. Yang, J.S. Wu, R.H. Wang, F.H. Lu, and C.J. Chang, Short sleep duration associated with a higher prevalence of metabolic syndrome in an apparently healthy population. Prev Med 55 (2012) 305-309.

[78] H. Xu, C. Liang, J. Zou, H. Yi, J. Guan, M. Gu, Y. Feng, and S. Yin, Interaction between obstructive sleep apnea and short sleep duration on insulin resistance: a large-scale study : OSA, short sleep duration and insulin resistance. Respir Res 21 (2020) 151.

[79] M. Yan, Z. Fu, T. Qin, N. Wu, Y. Lv, Q. Wei, H. Jiang, and P. Yin, Associations of sleep duration and prediabetes prevalence in a middle-aged and elderly Chinese population with regard to age and hypertension: The China Health and Retirement Longitudinal Study baseline survey. J Diabetes 10 (2018) 847-856.

[80] H. Yoo, and W.D. Franke, Sleep habits, mental health, and the metabolic syndrome in law enforcement officers. J Occup Environ Med 55 (2013) 99-103.

[81] Y. Zheng, A. Wang, C. Pan, J. Lu, J. Dou, Z. Lu, J. Ba, B. Wang, and Y. Mu, Impact of night sleep duration on glycemic and triglyceride levels in Chinese with different glycemic status. J Diabetes 7 (2015) 24-30.

[82] D.E. Rae, P.R. Pienaar, R.H.P. Henst, L.C. Roden, and J.H. Goedecke, Associations between long self-reported sleep, obesity and insulin resistance in a cohort of premenopausal Black and White South African women. Sleep Health 4 (2018) 558-564.

[83] T. Akiyama, T. Yamakawa, K. Orime, J. Suzuki, R. Sakamoto, M. Matsuura-Shinoda, E. Shigematsu, K. Takahashi, M. Kaneshiro, T. Asakura, S. Tanaka, T. Kawata, Y. Yamada, T. Isozaki, A. Takahashi, U.N. Osada, K. Kadonosono, and Y. Terauchi, Sleep duration and food intake in people with type 2 diabetes mellitus and factors affecting confectionery intake. Journal of Diabetes Investigation 14 (2023) 716-724.

[84] N.Y.Q. Tan, J. Chan, C.Y. Cheng, T.Y. Wong, and C. Sabanayagam, Sleep Duration and Diabetic Kidney Disease. Front Endocrinol (Lausanne) 9 (2018) 808.

[85] R. Yamamoto, Y. Nagasawa, H. Iwatani, M. Shinzawa, Y. Obi, J. Teranishi, T. Ishigami, K. Yamauchi-Takihara, M. Nishida, H. Rakugi, Y. Isaka, and T. Moriyama, Self-reported sleep duration and prediction of proteinuria: a retrospective cohort study. Am J Kidney Dis 59 (2012) 343-55.

[86] Y. Ye, L. Zhang, W. Yan, A. Wang, W. Wang, Z. Gao, X. Tang, L. Yan, Q. Wan, Z. Luo, G. Qin, L. Chen, S. Wang, Y. Wang, and Y. Mu, Self-reported sleep duration and daytime napping are associated with renal hyperfiltration and microalbuminuria in an apparently healthy Chinese population. PLoS One 14 (2019) e0214776.

[87] Y. Ye, L. Zhang, A. Wang, Y. Wang, S. Wang, G. Ning, and Y. Mu, Association of sleep duration with stroke, myocardial infarction, and tumors in a Chinese population with metabolic syndrome: a retrospective study. Lipids Health Dis 19 (2020) 155.

[88] X. Han, B. Liu, J. Wang, A. Pan, Y. Li, H. Hu, X. Li, K. Yang, J. Yuan, P. Yao, X. Miao, S. Wei, Y. Wang, Y. Liang, X. Zhang, H. Guo, H. Yang, F.B. Hu, T. Wu, and M. He, Long sleep duration and afternoon napping are associated with higher risk of incident diabetes in middle-aged and older Chinese: the Dongfeng-Tongji cohort study. Ann Med 48 (2016) 216-23.

[89] W. Zeng, L. Zhang, B. Feng, H. Li, D. Wang, Z. Zheng, Y. Zhang, L. Jiang, and H. Ye, Association between sleep disturbance with motoric cognitive risk syndrome in Chinese older adults. Eur J Neurol 28 (2021) 1470-1478.

[90] F. Zhou, J.F. Fu, C.L. Wang, and L. Liang, [Relationship between sleep duration and metabolic syndrome in obese children]. Zhonghua Liu Xing Bing Xue Za Zhi 31 (2010) 455-8.
